# Highly Sensitive Suspension Immunoassay for Multiplex Detection, Differentiation, and Quantification of Eight *Staphylococcus aureus* Enterotoxins (SEA to SEI)

**DOI:** 10.3390/toxins17060265

**Published:** 2025-05-24

**Authors:** Paulin Dettmann, Martin Skiba, Daniel Stern, Jasmin Weisemann, Hans Werner Mages, Nadja Krez, Martin B. Dorner, Sara Schaarschmidt, Marc A. Avondet, Marcus Fulde, Andreas Rummel, Birgit Strommenger, Sven Maurischat, Brigitte G. Dorner

**Affiliations:** 1 Centre for Biological Threats and Special Pathogens, Biological Toxins (ZBS3), Robert Koch Institute, 13353 Berlin, Germany; dettmannp@rki.de (P.D.); skibam@rki.de (M.S.); sternd@rki.de (D.S.); magesh@rki.de (H.W.M.); dornerm@rki.de (M.B.D.); 2 Institute for Toxicology, Hannover Medical School, 30625 Hannover, Germany; weisemann.jasmin@mh-hannover.de (J.W.); krez.nadja@mh-hannover.de (N.K.); rummel.andreas@mh-hannover.de (A.R.); 3 Department Biological Safety, German Federal Institute for Risk Assessment, 12277 Berlin, Germany; sara.schaarschmidt@bfr.bund.de (S.S.); sven.maurischat@bfr.bund.de (S.M.); 4 Federal Office for Civil Protection, Spiez Laboratory, 3700 Spiez, Switzerland; marc.avondet@bluewin.ch; 5 Centre of Infection Medicine, Institute of Microbiology and Epizootics, Freie Universität Berlin, 14163 Berlin, Germany; marcus.fulde@fu-berlin.de; 6 Department of Infectious Diseases, National Reference Centre (NRC) for Staphylococci and Enterococci, Division of Nosocomial Pathogens and Antibiotic Resistances, Robert Koch Institute, 38855 Wernigerode, Germany; strommengerb@rki.de

**Keywords:** staphylococcal enterotoxin, food poisoning, food safety, monoclonal antibodies, multiplex, suspension immunoassay, sandwich ELISA

## Abstract

Staphylococcal enterotoxins (SEs) are major contributors to foodborne intoxications. Reliable detection methods for SEs are essential to maintain food safety and protect public health. Since the heat-stable toxins also exert their toxic effect in the absence of the bacterium, reliance on DNA detection alone can be misleading: it does not allow for determining which specific toxins encoded by a given strain are produced and epidemiologically linked with a given outbreak. Commercially available diagnostic assays for SE detection are so far limited in sensitivity and specificity as well as in the range of targeted toxins (SEA–SEE), thus non-targeted SEs linked to foodborne illness remain undetected at the protein level. This study aimed to develop a highly sensitive and specific multiplex suspension immunoassay (SIA) for SEA to SEI. To this end, high-affinity monoclonal antibodies (mAbs) for the specific detection of the individual SEs were generated. When implemented in sandwich ELISAs and multiplex SIA, these mAbs demonstrated exceptional sensitivity with detection limits in the low picogram per millilitre range. When applied for the analysis of SE production in liquid cultures of a panel of 145 whole-genome sequenced strains of *Staphylococcus* spp. and *Enterococcus faecalis*, the novel multiplex SIA detected and differentiated the eight SEs with assay accuracies of 86.9–100%. Notably, the multiplex SIA covered one to four sequence variants for each of the individual SEs. Validation confirmed high recovery rates and reliable performance in three representative complex food matrices. The implementation of the novel mAbs in a multiplex SIA enabled, for the first time, simultaneous detection, differentiation, and quantification of multiple SEs from minimal sample volumes using Luminex^®^ technology. As a result, the multiplex SIA will help strengthen food safety protocols and public health response capabilities.

## 1. Introduction

*Staphylococcus aureus* is a ubiquitous pathogen colonising the nasal microbiota of 30% of the global population [1]. It is most prominently known as a major contributor to nosocomial infections. The treatment of such infections is becoming increasingly challenging due to the emergence of methicillin-resistant *S. aureus* strains, which exhibit a broad antimicrobial resistance [2]. In addition to genes encoding for antibiotic resistance, *S. aureus* harbours a number of virulence factors, including enterotoxins [3,4]. Staphylococcal enterotoxins (SEs) are known for their potent role as superantigens (SAgs), which are responsible for the development of toxic shock syndrome, with an inhalational median lethal dose (LD_50_) of >29 µg SEB/kg body weight in humans [5,6]. The most intensively studied SAg is SEB, which was pursued as a biological weapon until 1969 [7]. Consequently, SE types A, B, C, D, and E have been explicitly identified as select agents by the Centers for Disease Control and Prevention [8]. The development, production, and stockpiling of toxins have been categorically prohibited by the Biological Weapons Convention, which resulted in the destruction of the SEB stockpile in 1972 [9].

In addition to these notable roles, SEs represent a significant causative agent of food poisoning caused by bacterial toxins in the EU [10,11]. The most recent EU One Health Zoonoses report refers to 207 outbreaks due to *S. aureus* toxins, ranking second in 903 foodborne outbreaks caused by bacterial toxins. With 113 hospitalisations, SEs were responsible for the highest number of hospitalisations caused by bacterial toxins in 2023. The majority of foods contaminated with SEs are meat, meat products, mixed foods, milk, and dairy products such as cheese [12]. Nevertheless, the precise magnitude of staphylococcal food poisoning (SFP) outbreaks remains unknown due to underreporting: as a usually self-limiting disease with a broad spectrum of case severity, food poisonings are not always comprehensively diagnosed [10,13]. The typical rapid onset of symptoms associated with SFP occurs following the ingestion of low amounts of toxins. The uptake of 6.1 ng of SEA has been shown to be sufficient to trigger SFP [14]. The initial symptoms typically manifest within 30 min to 8 h, and consist of nausea, abdominal cramps, and vomiting. In some cases, diarrhoea may also emerge. However, spontaneous recovery typically occurs within 24 h [15,16]. Furthermore, SEs exhibit high resilience to heat and most proteolytic enzymes, enabling them to remain emetically active even after exposure to cooking temperature and ingestion [17,18,19].

Structurally and functionally related toxin types constitute a large family of SEs. To date, 33 distinct SE types and SE-like proteins have been identified, exhibiting significant variability, with amino acid sequence identities ranging from 21% between SEC and SEI to 83% between SEA and SEE [12,20,21,22]. This has led to a classification of SEs into groups according to a high degree of structural similarity and with molecular weights of 22 to 28 kDa [23,24,25,26,27]. The International Nomenclature Committee for Staphylococcal Superantigens has provided a guideline for the alphabetically based nomenclature of SEs. The initially identified five SEs (SEA, SEB, SEC, SED, and SEE) are referred to as the classical SEs, whereas SE types that have been subsequently discovered are designated as newly described SEs. A new type is identified by a sequence divergence of greater than 10% from previously identified SEs [28]. Next to subtypes, especially known from SEC with SEC1, SEC2, and SEC3, numerous molecular variants, including truncated proteins, have been reported for various existing SEs sharing 91–99% identity. These molecular variants have the potential to negatively impact diagnostic methods through “escape mutations” [29,30,31]. The newly discovered superantigens that have not been proven to cause emesis and thus have an unclear role in SFP outbreaks are designated “staphylococcal enterotoxin-like” (SEl) SAgs [28].

SFP outbreaks are frequently attributed to the classical group of SEs, with SEA identified as the most prevalent type (approximately 80% of cases where SE types were identified) [19,32,33,34,35]. In recent years, the involvement of other enterotoxins, including SEG, SEH, and SEI, has also been identified as a potential cause of SFP [36,37,38,39,40,41,42,43,44]. Monovalent enterotoxigenic strains are uncommon, implying that multiple SEs may be present in SFP samples and thus necessitating their detection, if possible simultaneously [29,45]. Molecular biology techniques, including PCR and whole genome sequencing (WGS), facilitate the accurate detection of *se* genes encoded in strains. DNA-based methods, however, usually require the isolation of staphylococcal strains from food samples, and only indicate the theoretical SE production potential of a strain, rather than the actual toxin production that is causing the symptoms. A plethora of diagnostic techniques have been devised to target SEs on the protein level. The most prevalent and utilised methods encompass bioassays, mass spectrometry, and immunoassays [46,47]. Traditional in vivo bioassays, foundational for detection methods of SFP outbreaks, are now considered inadequate due to their low sensitivity and specificity, impracticality, and ethical concerns. Mass spectrometry-based methods represent a valuable addition to the toolkit for SE detection, offering the ability to identify at least 24 SEs [48,49,50]. However, the advantages are also accompanied by high analysis costs, limited throughput, expert staffing requirements, and extensive sample preparation to avoid interference in complex samples. Therefore, the most commonly employed diagnostic approach for direct SE protein detection in food is based on the use of polyclonal or monoclonal antibodies applied in immunoassays, such as latex agglutination, immunoblotting, or immunochromatography.

In addition to reverse passive latex agglutination (RPLA) [51], the commonly used enzyme-linked fluorescence assay (ELFA) or enzyme-linked immunosorbent assay (ELISA)-based commercial kits are available. The VIDAS SET2 (bioMérieux, Marcy l’étoile, France) [52] and the RIDASCREEN^®^ SET Total and SET A, B, C, D, E (both R-Biopharm, Darmstadt, Germany) [53] are available for the detection of SEA to SEE in different sample matrices, with only the RIDASCREEN^®^ SET A, B, C, D, E kit being able to differentiate the classical SE types [52,54]. Both the VIDAS^®^ SET2 and the RIDASCREEN^®^ SET Total are the screening and confirmatory assays currently routinely used in food safety laboratories for the detection of SE in food and are considered to fulfil the performance criteria specified in the EN ISO 19020 standard [55]. Commercial assays that detect SEs beyond the classical group are limited but necessary, as shown by recent SFP outbreaks involving non-classical SEs [36,37,38,39,40,41,42,43,44]. One of these is the VIDAS^®^ SET3 (bioMérieux, Craponne, France) [54], which detects SEG to SEI without differentiation but has not yet been available on the market. Research-type lateral flow assays (LFAs) and ELISAs have been developed that can detect SEA to SEI but are not commercially available to date [56,57,58,59]. While these methods are a diagnostic advancement, they are hampered by the need for high sample volumes and the inability to simultaneously detect and differentiate more than three SE types without cross-reactivity. To date, there is a gap and a need for a routine immunological method that can simultaneously and specifically detect and quantify SEA to SEI in minimal sample volumes.

To address this issue, this work is based on the Luminex^®^ technology. As described by Pauly et al. [60], the primary antibodies are bound to individual sets of fluorescently coded microbeads, which are spectrally unique by embedding of precise ratios of red and infrared dyes and which can be discriminated by flow cytometry. The different antibody-coated fluorescent microbeads are then added to 50 µL of the sample and simultaneously incubated. After washing, a mixture of biotinylated detection antibodies specific for the different antigens in the multiplex suspension immunoassay (multiplex SIA) is added, and the binding of antigens present in the sample to individual bead sets is detected by streptavidin–phycoerythrin as the reporter. With a two-laser system, the beads are excited by one which allows for the identification of the bead set corresponding to a given antigen, while the second laser determines the reporter signal phycoerythrin associated with the bead sets, allowing for the interpretation of the analyte’s quantity in the sample.

The objective of this study was to develop a sensitive and specific multiplex SIA capable of recognising a wider range of SEs (not only the classical ones), including their variants. The simultaneous detection of multiple SEs in a single assay reduces the time, resources, and sample volume required compared to performing assays for each toxin separately. In this study, new mAbs were generated and characterised against SEA to SEI. These mAbs were integrated into a conventional single-toxin sandwich ELISA for the detection of SE and subsequently evaluated for sensitivity and specificity in a validation study. Suitable mAbs against SEA to SEI were implemented in a second step in a multiplex SIA using the Luminex^®^ platform, which was validated for the matrices buffer, selected food matrices, and bacterial culture supernatants. The multiplex SIA achieved high sensitivity with detection limits of 5 to 15 pg/mL and high specificity, ensuring accurate identification of all SE types tested in the panel, namely SEA to SEI. Unlike conventional methods, this multiplex SIA provided individual quantitative data for each analyte, which is crucial for understanding the contamination levels in different samples. Overall, the novel SE multiplex SIA turned out to be a robust and efficient approach for comprehensive monitoring and study of SEs, enhancing the ability to detect and respond to potential health threats posed by these toxins.

## 2. Results

### 2.1. Generation and Characterisation of High Affinity Monoclonal Antibodies (mAbs)

In order to generate high-affinity and specific mAbs against SEs, mice were immunised separately with SEs (SEA, SEB, SEC–SEC1, SEC2, and SEC3–, rSED, SEE, rSEG, rSEH, or rSEI) and nine hybridoma fusions were performed, followed by a comprehensive screening using native and/or recombinant SEs. The identity and purity of the native and recombinant SEs used as antigens were verified by SDS-PAGE (Figure S1), and their amino acid sequence coverage was analysed by tryptic digest and tandem mass spectrometry analysis (LC-MS/MS) (Figure S2). The screening resulted in the selection of 20 new antibodies, which were selected based on their superior performance: SEA165, SEA388, and SEA2353 (anti-SEA); S419, S1001, and S1851 (anti-SEB); SEC290 and SEC371 (anti-SEC); SED9, SED333, and SED1280 (anti-SED); SEE33 and SEE1524 (anti-SEE); SEG5 and SEG158 (anti-SEG); SEH449 and SEH1236 (anti-SEH); and SEI92, SEI242, and SEI467 (anti-SEI). Seven of these, S419, SED9, SED333, SED1280, SEE33, SEH449, and SEI92, were cloned and expressed as recombinant antibodies since the original hybridoma lines showed low or diminishing mAb production. All mAbs were characterised in detail for specificity against SEA to SEI by indirect ELISA, and binding affinities were determined by surface plasmon resonance (SPR) spectrometry (Table 1).

In the indirect ELISA format, all 20 mAbs were tested for their reactivity to SEA, SEB, SEC1, SEC2, SEC3, SED, SEE, rSEG, rSEH, and rSEI to evaluate their specificity by clearly distinguishing their target SE type from others. All mAbs exhibited specific reactivity to their respective antigen, and the mAbs SEC290 and SEC371 were able to detect SEC1, SEC2, and SEC3. Eight mAbs, SEA165, SEA2353, S419, S1001, S1851, SEE33, SEG5, and SEH1236, also recognised one or two additional SEs with lower reactivity compared to their primary target enterotoxin. The highest cross-reactivity to other SEs was observed for the highly similar SE types sharing more than 65% identity at the amino acid level: SEA and SEE (mAb SEA165 and mAb SEA2353 additionally detected SEE), and SEB and SEC (mAb S1001 detected SEC1) (Table 1, Figure S3). All mAbs exhibited high affinities to their target SE, with *K*_D_ values ranging from 10^−12^ to 10^−9^ M. Although the *k*_a_ presented a narrow range (7.8 × 10^4^ to 9.2 × 10^5^ M^−1^s^−1^), the observed variability in the *k*_d_ (3.6 × 10^−3^ to 4.1 × 10^−6^ s^−1^) resulted in the different affinity constants (Table 1, Figure S4). For each antigen, two to three mAbs were selected in the screening that were compatible in a sandwich format and thus bound distinct epitopes as determined in an epitope binning approach (Table 1). As a result of the analysis, SEA165, SEA388, and SEA2353 bound three different epitopes of SEA; S419, S1001, and S1851 bound three different epitopes of SEB; SEC290 and SEC371 targeted SEC1 with two different epitopes; SED9, SED333, and SED1280 bound three different SED epitopes; SEE33 and SEE1524 were able to bind two different epitopes on SEE; SEG5 and SEG158 bound two epitopes on SEG; SEH449 and SEH1236 were compatible and thus bound two different epitopes of SEH; and SEI92 detected another epitope on SEI compared to SEI242 and SEI467.

### 2.2. Establishment and Validation of Eight Sandwich ELISAs for Highly Sensitive and Specific Detection of SEA to SEI

**Figure 1:**
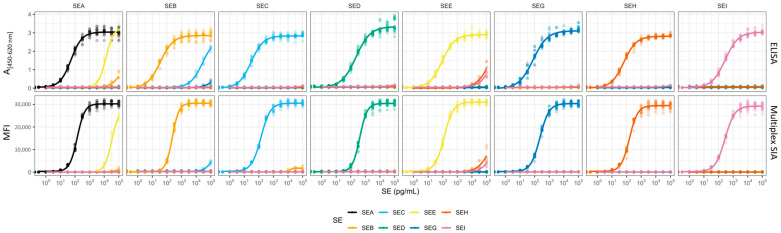
Detection of SEs by sandwich ELISAs and multiplex SIA. The sandwich ELISAs (top panel) were set up using specific combinations of immobilised capture mAbs, SEA388 + SEA2353 (for SEA), S1001 (SEB), SEC371 (SEC), SED1280 (SED), SEE33 (SEE), SEG5 (SEG), SEH1236 (SEH), and SEI467 (SEI). Each of these capture mAbs was paired with a corresponding detection mAb, SEA165 (SEA), S419 (SEB), SEC290 (SEC), SED333 (SED), SEE1524 (SEE), SEG158 (SEG), SEH449 (SEH), and SEI92 (SEI). The assays were performed in 96-well microtiter plates. For the multiplex SIA, the assay was adapted to a bead-based format (bottom panel). In this format, paramagnetic beads were coated with mAbs: SEA388 + SEA2353, S1851, SEC371, SED1280, SEE1524, SEG5, SEH449, and SEI242 (from left to right, specific for SEA to SEI) on different bead sets. Detection was achieved using a mixture of biotinylated detection mAbs: SEA165, S419, SEC290, SED9, SEE33, SEG158, SEH1236, and SEI92 (left to right, specific for SEA to SEI), allowing for simultaneous detection of multiple SE targets. Detection was performed after incubation of capture mAbs with serial dilutions of SE antigens—SEA (black), SEB (orange), SEC1 (light blue), SED (green), SEE (yellow), SEG (dark blue), SEH (brown), and SEI (purple)—in both assay formats. The ELISAs used single biotinylated mAbs in combination with streptavidin–horseradish peroxidase for detection, while the multiplex SIA used a mixture of biotinylated mAbs and streptavidin–phycoerythrin. The results were recorded as A_[450–620nm]_, corresponding to the absorbance measured at 450 nm minus the one at 620 nm, for the individual ELISAs and as the MFI (median fluorescent intensity) for the multiplex SIA. The results were modelled using a semi-logarithmic four-parameter fit with log-transformed concentration values (*x*-axis). Data for the specific target antigens in their respective ELISA or on their specific beads were collected from five independent experiments, each measured in duplicate (*n* = 5). In addition, to assess potential cross-reactivity, each specific SE antigen was tested on the other respective seven ELISAs or bead sets in three independent experiments (*n* = 3).

As a starting point, the sandwich ELISA technique was developed because of its widespread use in routine laboratories. Suitable mAbs were combined to establish sandwich ELISAs for the highly sensitive and specific detection of individual toxins. In one case (SEA ELISA), two capture mAbs were used to obtain optimal results; in all other cases, single capture mAbs were combined with single detection mAbs. Individual dilution series of each SE type, from SEA to SEI, were tested in all SE-specific sandwich ELISAs to highlight specific signals and potential cross-reactivities (CR) (Figure 1). With regard to specificity, six of the eight ELISAs targeting SEC, SED, SEE, SEG, SEH, or SEI showed excellent specificities for their respective SE only (CR with respect to the specific target SE < 0.1%). Slight CR of the SEA-specific ELISA with SEE (CR = 1.7 ± 0.8%) and of the SEB-specific ELISA with SEC1 (CR = 0.2 ± 0.01%) were observed, which could be explained by the high sequence identity between SEA and SEE (83.3%) and between SEB and SEC (68.9%), respectively, which became already visible on the level of the corresponding mAbs (Table 2). The ELISAs were validated for the detection of toxins in buffer according to an international consensus protocol for ELISA validation [62]. Based on the blanks measured in buffer without antigen, the theoretical limit of detection (LoD_th_) for each ELISA was estimated to be between 0.4 and 6.1 pg/mL. The LoDs were confirmed experimentally (LoD_exp_) by spiking at 5 pg/mL for the detection of SEA, SEB, SEC1, SEE, rSEG, or rSEH, and at 10 pg/mL for the detection of SED or rSEI into buffer (Table 3, Figure S5). The half-maximal effective concentration (EC_50_) values were determined from the resulting sigmoidal ELISA dose–response curves and used to assess the sensitivity of these mAb combinations, as shown in Table 3. The quantifiable range varied from 6 to 200 pg/mL for SEA detection and up to 30 to 800 pg/mL for SEI detection (Table 3). The repeatability of the assays was tested by analysing the variability of four biological replicates of two values around the EC_50_ on the same day (CV_intra_) and five replicates of five toxin concentrations on five separate days (CV_inter_). The mean inter- and intra-assay precision values for all analytes showed low variability between 4.1–9.8% and 4.9–9.0%, respectively (Table 3), which is within the acceptable range of 20 to 25% [63,64].

### 2.3. Establishment and Validation of Bead-Based Multiplex SIA for the Highly Sensitive, Specific, and Simultaneous Detection of SEA to SEI

Compared to the traditional sandwich ELISA, the multiplex SIA enables the simultaneous detection of multiple analytes in a single sample. Following the establishment of highly sensitive sandwich ELISAs, the aim was to combine them into a bead-based multiplex SIA. The construction of a multiplex SIA requires highly specific mAbs to avoid or minimise cross-reactions among the multiple mAbs and multiple analytes in the immunodetection approach, which do not play a role in sandwich ELISAs with their limited number of mAbs and analytes present in one reaction (Figure S6). While setting up a multiplex SIA, potential additional cross-reactivities have to be considered and minimised, namely cross-reactivities of all antigens with all antibodies present in the mixture and cross-reactivities among all antibodies in the approach. Cross-reactivity was evaluated using dilution series of individual SEs, while maintaining the general multiplex SIA set up (“single-toxin detection mode”, Figure S6). This included a mixture of eight mAb-coated bead regions and a mixture of eight biotinylated detection mAbs, but the use of only one particular SE. Compared to the sandwich ELISAs, the antibody selection for the multiplex SIA (Table 4) had to be modified for individual SEs, either by switching the orientation of the capture and detection antibodies (for SEE and SEH) or by employing less cross-reactive mAb combinations (as for SEB, SED, and SEI by replacing S1001, SED333, and SEI467 with S1851, SED9, and SEI242, respectively) to increase specificity. The cross-reactivity of SEA beads with the highly homologous SEE was slightly reduced in the multiplex SIA (CR = 0.6%). No further cross reactivity (CR > 0.1%) was observed (Figure 1, Table 2).

The validation of the multiplex SIA followed the principles of ELISA validation. The estimated LoD_th_ per target was based on blank (buffer without antigen) measurements and ranged from less than 1 to 4.1 pg/mL. The LoD_exp_ were verified experimentally by spiking a toxin mixture with 5 pg/mL each of SEA, SEC1, SED, SEE, rSEG, rSEH, and rSEI and 15 pg/mL of SEB (“multi-toxin detection mode”, Figure S6 and Table 4). In addition, the LoD_exp_ was further tested on single toxins (“single-toxin detection mode”) and was confirmed for all the above SEs in the mixture except SED. The LoD_exp_ for single SED was confirmed at 10 pg/mL compared to 5 pg/mL in the multi-toxin detection mode (Figure S7). The EC_50_ values, also determined from the sigmoidal dose–response curves, provided sensitivity benchmarks for each target antigen detection as summarised in Table 4. The quantifiable ranges spanned from 75 to 300 pg/mL for SEA, 40 to 600 pg/mL for SEB, 6 to 400 pg/mL for SEC1, 200 to 900 for SED, 40 to 250 pg/mL for SEE, 110 to 650 pg/mL for SEG, 15 to 420 pg/mL for SEH, and 170 to 950 pg/mL for SEI detection. The multiplex SIA achieved intra- and inter-assay coefficients of variability ranging from 4.9 to 15.8%, which is well below the 20–25% acceptable limit considered for immunoassays (Table 4) [63,64].

### 2.4. Confirmation of Broad and Specific Recognition of SEA to SEI Produced by a Wide Range of Whole-Genome Sequenced S. aureus Strains in Liquid Culture

**Figure 2:**
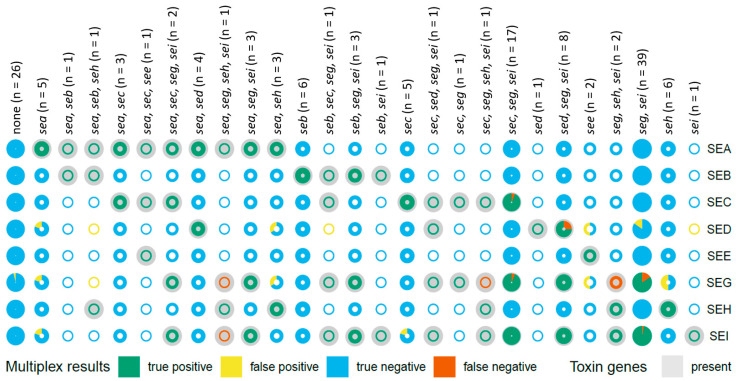
Multiplex SIA results for the detection of clustered native toxins produced by 145 bacterial strains. After immobilising capture mAbs on individual magnetic microbeads for the specific detection of SEA to SEI (mAbs SEA388 + SEA2353, S1851, SEC371, SED1280, SEE1524, SEG5, SEH449, and SEI242), a total of 145 culture supernatants were incubated as 1:10 diluted samples with the antibody-coated microbeads, washed, and detected by a mixture of biotinylated detection mAbs (SEA165, S419, SEC290, SED9, SEE33, SEG158, SEH1236, and SEI92) followed by streptavidin–phycoerythrin. The results based on ROC analysis (pROC package, version 1.18.0) using R (version 4.1.3) were compared to filtered *sea*- to *sei*-genic strains based on whole genome sequencing (WGS) data. The cluster size (number of strains) is given as n. The presence of toxins in the genomic sequence data is indicated by fields with a grey background. The colour of the circles denotes the correctness of the multiplex results in relation to the WGS data. Correctly identified SE protein results are shown as either green (true positive) or blue (true negative), while incorrect results are shown as either yellow (false positive) or orange (false negative). The thickness of the circle lines correlates positively with the cluster size.

A major challenge was the fact that many strains produce a range of different SEs, which placed considerable demands on the assay’s ability to discriminate between these SEs. As a result, multiplexing was a critical aspect of the methodology, allowing the simultaneous detection of multiple SE types. The aim was to assess the performance of the assay, in particular the sensitivity and specificity of SE detection in supernatants of *S. aureus* liquid cultures. To this end, 145 WGS bacterial strains were analysed (Figure 2, Table S1). Of these, 123 *S. aureus* strains encoded at least one SE (containing one of the following genes: *sea-see*, *seg-selz*, or *tst*) while 22 non-enterotoxigenic bacterial strains, including *S. aureus*, *S. epidermidis*, *S. haemolyticus,* and *Enterococcus faecalis*, were tested as controls. Four enterotoxigenic strains did not contain *sea* to *sei* and were additional controls for the SEA to SEI detection; thus, the cultural supernatants of 26 strains did not contain SEA to SEI (Figure 2). The few monovalent enterotoxigenic strains (containing one of the following genes: *sea-see*, *seg-selz*, or *tst*) harboured genes for either *sea* (*n* = 4), *seb* (*n* = 2), *seh* (*n* = 3), *sep* (*n* = 3), or *tst* (*n* = 1). When only the eight target SE types for SEA to SEI were considered, *sea* (*n* = 5), *seb* (*n* = 6), *sec* (*n* = 5), *sed* (*n* = 1), *see* (*n* = 2), *seh* (*n* = 6), and *sei* (*n* = 1) were encoded without the other SE types targeted by the ELISAs and by the multiplex SIA and these numbers are indicated in Figure 2.

The multivalent strains were first analysed to examine the frequently co-encoded *se* genes (Figure S8). This analysis revealed a high correlation between the presence of *sei*, *seg*, *sem*, *sen*, *seo*, and *selx.* The first five mentioned are part of the enterotoxin gene cluster (*egc*), of which only SEI and SEG were addressed in the current study. Furthermore, 26 clusters of strains encoding at least one of the *sea* to *sei* genes were identified (Figure 2).

The detection of native toxins produced by enterotoxigenic strains is crucial but challenging since molecular variants of individual SEs exist [29]. Based on the information published by Merda et al., sequence variants for SEA to SEI with amino acid differences of up to 9% have been described. They pose the risk of “escape mutants” that may evade detection by the diagnostic techniques applied. Indeed, among the strains analysed in this study, two protein variants of SEA with 98.1%, four SEB variants with 96.3 to 99.3%, four SEC variants—including SEC1, SEC2, and SEC3—with 93.3 to 98.1%, one SED variant, one SEE variant, three SEG variants with 96.9 to 98.8%, two SEH variants with 98.8%, and three SEI variants with 93.0 to 96.7% identity at amino acid level were observed (Table S2). Two and three strains were found to contain truncated *sed* and *seg* genes, respectively. These truncated genes have been previously observed in *S. aureus* strains [30,41].

To evaluate the performance of the multiplex SIA in terms of sensitivity (true positive rate) and specificity (true negative rate), the WGS data of the strains were compared with direct protein detection in liquid culture supernatants. To this end, the supernatants were tested at a 1:10 dilution by multiplex SIA for the detection of SEA to SEI. A receiver operating characteristic (ROC) analysis was performed to determine threshold values (Figure S9). In the presence of the gene encoding for the specific SE type, the result was classified as an event. Conversely, in the absence of the gene, the result was designated as a control (*n* = 26) (Figure 2, Table S1).

**Figure 3:**
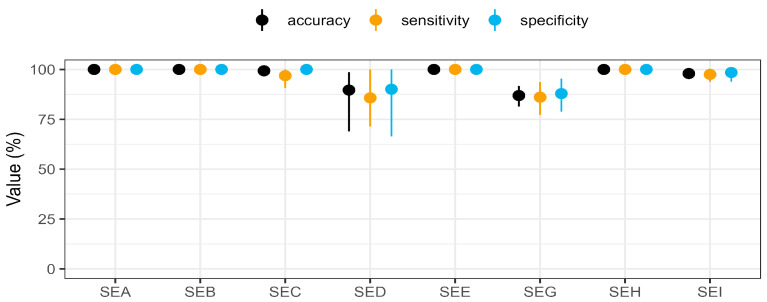
Performance parameters of the multiplex SIA for the detection of native toxins in bacterial culture supernatants. After immobilisation of the capture mAbs on paramagnetic microbeads, a total of 145 culture supernatants were diluted in a ratio of 1:10, incubated, and detected with a mixture of the detection mAbs directed against SEA to SEI (see Figure S6) and streptavidin–phycoerythrin. The results from three independent experiments (*n* = 3), analysed by ROC analysis (pROC package, version 1.18.0) using R (version 4.1.3), were compared with whole genome sequencing data of the strains and calculated as performance metrics. The data are presented with median values (dots) and confidence intervals (bars) for each SE detected in the multiplex SIA, using three colours to represent the performance metrics: accuracy (black), sensitivity (orange), and specificity (blue).

**Figure 4:**
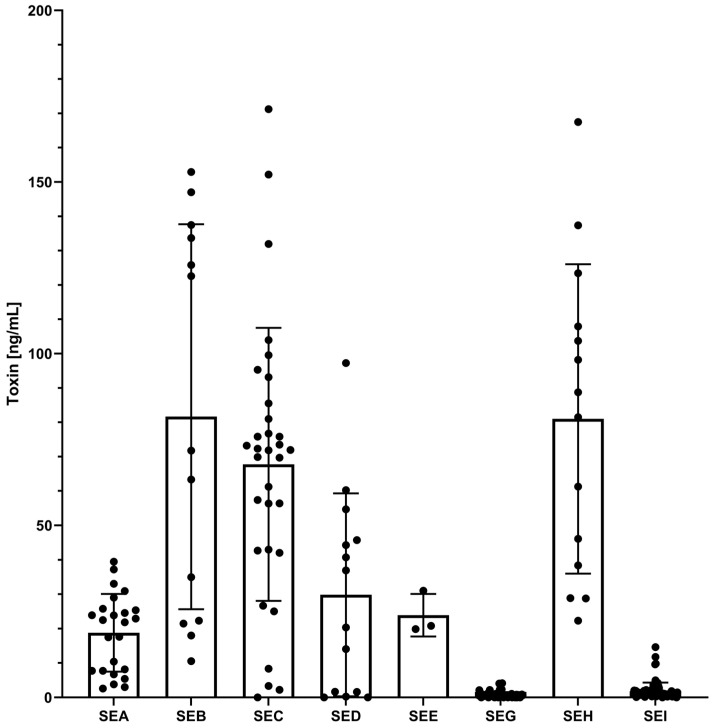
Overview of the estimated toxin concentration in all supernatants obtained from *sea-* to *sei*-genic strains measured by multiplex SIA. After immobilisation of the capture mAbs on paramagnetic microbeads, a set of 145 culture supernatants was incubated as samples. SE detection was performed using a mixture of biotinylated detection mAbs, followed by the addition of streptavidin–phycoerythrin. In this experiment (*n* = 2), the results were extrapolated to the toxin dilution curve, with each concentration per target and supernatant from the target-encoding strain represented as a black dot. The number of black dots reflects the number of enterotoxigenic strains for each specific target SE; thus, only true positives and false negatives are shown. The mean concentration values across all target *se*-genic strains are depicted as a bar, with the standard deviation included. For an overview of the estimated toxin concentration in the 145 strains depicted over the different *se* variants, please see Figure S10.

The results of the multiplex SIA are shown in Figure 2, Figure 3 and Figure 4, Figure S10 and Table S1. With the assessed thresholds by ROC analysis, median fluorescent intensity (MFI) signals above 6030 MFI (54.9 ± 3 pg/mL) for SEA, 16,979 MFI (268.3 ± 20 pg/mL) for SEB, 8854 MFI (66.7 ± 2 pg/mL) for SEC, 2121 MFI (87.5 ± 17 pg/mL) for SED, 16,886 MFI (147.1 ± 12 pg/mL) for SEE, 1290 MFI (19.7 ± 2 pg/mL) for SEG, 15,680 MFI (177.6 ± 20 pg/mL) for SEH, and 794 MFI (12.4 ± 1 pg/mL) for SEI detection, the multiplex SIA was able to detect the eight targets in the culture supernatants with high specificity and sensitivity. A total of 100% sensitivity and specificity were achieved in all clusters for the detection of SEA (*n* = 24 strains), SEB (*n* = 13 strains), SEE (*n* = 3 strains), and SEH (*n* = 14 strains). After an estimation of toxin concentrations in the supernatants, SEA, SEB, SEE, and SEH were expressed at least above 2.5 ± 1.8, 10.5 ± 5.7, 19.9 ± 6.2, and 22.3 ± 6.3 ng/mL, respectively, under the culture conditions used (Figure 4, Figure S10). The measured concentrations of each SE type variant were tested for normal and lognormal distribution by the Shapiro–Wilk test. The two SEA variants showed lognormal distribution and a significant difference (*p*-value < 0.0001) between their mean concentrations by *t*-test (Figure S10). The difference between the mean concentrations of the variants of SEB (four variants, lognormal distribution) and SEH (two variants, lognormal distribution) was not statistically significant (*p*-value > 0.05) in a one-way ANOVA test and *t*-test. SED and SEE had only one variant each.

SEC was successfully detected for 31 of 32 *sec*-genic strains, without any false positive results (Figure 2, Table S1). With no statistically relevant difference in mean concentration levels between the four SEC variants (lognormal distribution), SEC was expressed at a minimum of 2.2 ± 0.1 ng/mL (Figure 4 and Figure S10). Only in the culture supernatant of strain 20-01064, harbouring *sec*, *seg*, *sei*, *sel*, *sem*, *sen*, *seo*, and *selx*, and thus representing one of the 17 strains in the cluster encoding *sec*, *seg*, and *sei* simultaneously, did SEC remain undetectable (Figure 2, Table S1). This resulted in 100% specificity and 96.9% sensitivity for SEC detection by multiplex SIA (Figure 3).

A sensitivity of 85.7% was achieved for the detection of SED, with 12 correct results for the 14 *sed*-genic strains. The lack of SED detection in culture supernatants of the strains 16-00175 and 16-00532 can be explained by the absence of promoter regions in their sequences which prohibits SED expression [30] (Table S1). Truncated SED was detected at 0.3 ± 0.1 and 1.7 ± 0.1 ng/mL in the culture supernatants from strains 18-ST00095 and 21-01535, respectively. The two false negative results among the 14 *sed*-genic strains were observed in one cluster of strains co-encoding *sed*, *seg*, and *sei* (*n* = 8 strains) (Figure 2). Additionally, 12 strains in seven different *se* clusters of the 133 strains that did not harbour *sed* were falsely identified as SED producers, resulting in a specificity of 90.8%.

A total of 81 strains contained the *egc* operon, with 78 strains co-encoding *seg* and *sei*. While 68 of 79 *seg*-genic strains were correctly identified (Table S1) and contained at least 0.13 ± 0.01 ng/mL SEG in their culture supernatants, 11 remained undetected, resulting in a sensitivity of 86.1% (Figure 3). One of the three culture supernatants from truncated *seg*-genic strains (14-00594) was found to contain SEG, demonstrating the assay’s principal ability to detect this truncated SEG (Table S1). With 12 *seg* encoding clusters, SEG results were false negatives in four of these clusters. With eight false positives, the specificity of SEG detection was 87.9% (Figure 3). The only false positive result for strains encoding no *sea* to *sei* genes was seen on SEG beads in the culture supernatant of strain 15-ST00398 encoding *sep*, with further false positive results in seven additional *se*-encoding clusters. The median concentration of SEG showed a statistically relevant difference between their variants (*p* < 0.05) in a Kruskal–Wallis test, with SEG providing the lowest overall protein expression level of 0.9 ± 0.8 ng/mL among the eight SEs analysed in this study.

Culture supernatants from 80 *sei*-genic strains were measured, and 78 were correctly detected (Figure 3, Table S1), providing a sensitivity of 97.5%. The concentrations of the SEI variants did not show a statistically relevant difference (*p*-value > 0.05) by the Kruskal–Wallis test. Two false negatives were observed in two clusters. Two false positives in two different clusters resulted in a specificity of 98.5%. The false negative results for SEG and SEI were caused once by the same strain (13-ST00641), suggesting a possible non-production or production below the limit of detection of the multiplex SIA.

Figure 2 summarises the comprehensive data set provided in Table S1 and Figure 3 on the number of true positive, true negative, false positive, and false negative multiplex SIA results obtained for the 145 strains evaluated, depicted against the different *se* cluster types. The colour of the circles denotes the correctness of the multiplex results in relation to the WGS data, which were taken as “truth”. The overview shows the number of true positive and true negative results in green and blue, while the incorrect results in yellow (false positive) and orange (false negative) are visible as well. The grey background indicates the presence of *se* genes in the strains (and is also provided at the top of each column), but it is completely covered in highly frequent *se* gene combinations such as *sec*, *seg*, and *sei* (*n* = 17).

In order to compare the performance of the multiplex SIA with conventional ELISA, all bacterial culture supernatants were additionally tested and evaluated with the eight individual sandwich ELISAs and their thresholds were calculated as described for the multiplex SIA (Table S3, Figures S11 and S12). Here, signals were counted as positive if they exceeded 8.5 ± 1 pg/mL for SEA, 18.6 ± 2 pg/mL for SEB, 18.8 ± 2 pg/mL for SEC, 26.0 ± 2 pg/mL for SED, 53.9 ± 9 pg/mL for SEE, 12.8 ± 1 pg/mL for SEG, 29.6 ± 2 pg/mL for SEH, and 45.2 ± 5 pg/mL for SEI (Table S3).

All sandwich ELISAs were able to detect the targeted SE types and their molecular variants (Table S3). SEB, SEE, and SEH were detected with 100% sensitivity and specificity. The SEA sandwich ELISA achieved 100% sensitivity, but two false positives resulted in a specificity of 98.3%. The two false positive results were probably caused by SEE (being 83.3% identical on amino acid level to SEA) in the culture supernatants of strains 08S00574 and 09S00575, which both harbour *see* and *seq* genes. The specificity and sensitivity of SEC detection were as described for the multiplex SIA (specificity = 100%; sensitivity = 96.9%), with the same strain 20-01064 remaining undetectable also by ELISA. While the SED ELISA correctly identified the same 12 strains as the multiplex SIA, the SED ELISA gave only two false positives, and thus resulted in 98.5% specificity. A total of 8 out of 79 strains harbouring *seg* were false negatives in the SEG ELISA, resulting in a sensitivity of 89.9%. With no false positives, the SEG ELISA achieved 100% specificity. With a sensitivity of 93.8% and a specificity of 95.4%, the SEI ELISA detected 75 out of 80 *sei*-genic strains and had three false positives (Table S3, Figure S12). Overall, each molecular variant of SEA to SEI was principally detectable in the supernatants both by the multiplex SIA and the sandwich ELISA.

To confirm the binding of the mAbs to their respective SE in both immunoassays, at least one bacterial culture supernatant per SE type was selected for immunoaffinity enrichment using a mixture of mAbs against the SE types SEA to SEI. This was followed by tryptic digest and tandem mass spectrometry analysis (LC-MS/MS). The reference amino acid sequences were obtained through whole genome sequencing of the corresponding strains. With a protein sequence coverage ranging from 73 to 98%, the following SE types were identified using the mAbs described in this work: SEA, SEB, SEC1, SEC3, SED, SEE, SEG, SEH, and SEI (Figure S13). For this analysis, no bacterial liquid culture supernatants produced by a strain encoding sec.v4 (SEC2) were analysed.

### 2.5. Detection of SEA to SEI in Spiked Food Matrix Extracts by Using the Multiplex SIA

SFP outbreaks are typically associated with the ingestion of contaminated food, thus necessitating the final validation of the newly established multiplex SIA for the detection of SEs in food matrices. The selected matrices, namely raw milk, raw milk cheese, and smoked pork pâté, represent common foods associated with foodborne outbreaks. To evaluate the potential background signals caused by non-specific interactions with the assay components, extracts of the selected food matrices were tested prior to spiking with different SEs. The extracts were analysed in their undiluted state and at 1:10 and 1:100 dilutions in 0.1% BSA/PBS using the bead-based multiplex SIA (Figure S14). The highest background signals were observed in the undiluted extract of pork pâté, with values ranging from 189 MFI on SEB beads to 284 MFI on SEA beads. Nevertheless, these values remained below the LoD_exp_ for each target antigen, indicating a low risk of false positives. Once it was confirmed that the background signals did not exceed the LoD_exp_, the undiluted extracts of the food matrices were spiked with SE toxin mixtures (SEA to SEI together measured in the “multi-toxin detection mode”) in accordance with the consensus protocol outlined by Worbs et al. [62]. The concentration employed was equivalent to the EC_50_ values of the multiplex SIA and additionally ten times their EC_50_ values (see Table 4). The measured values of the target antigens were converted into toxin concentrations recovered from the food extracts. Initially, the theoretical concentrations of the spiked toxins were compared to the detectable concentrations of the target antigens in buffer. Since the difference between these values did not exceed 20%, the recovery rate was subsequently calculated by comparing the detectable toxin concentration in the food matrix extracts to that in the buffer.

**Figure 5:**
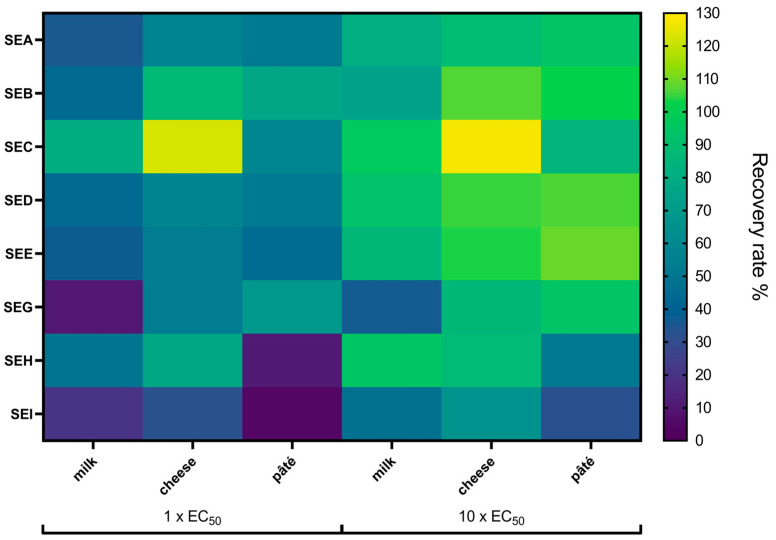
The recovery of SEA to SEI in undiluted food extracts by multiplex SIA. Magnetic microspheres were coated with capture mAbs directed against SEA to SEI on different bead sets. Undiluted food extracts were spiked with lower (1 × EC_50_) or higher (10 × EC_50_) concentrations of a toxin mixture containing SEA to SEI. Sample detection was conducted using a combination of SEA- to SEI-specific biotinylated detection mAbs, followed by the application of streptavidin–phycoerythrin. The measured values of the target antigens in the extracts and in buffer were converted into toxin concentrations. The efficiency with which a target antigen was detected in an extract, measured in five biological replicates compared to its detection in buffer, is shown here as recovery rate in per cent (for details see Table S4), indicated with a scale from yellow for high recovery and dark blue for low recovery.

The recovery rates exhibited variability based on the food matrix, the target antigen, and the toxin concentration (Figure 5, Table S4). A comparison of recovery rates across different matrices revealed that raw milk cheese consistently demonstrated the highest recovery levels, with an average of 67.6 ± 5.2% for low toxin concentrations and 96.3 ± 7.8% for high concentrations. In contrast, the raw milk extract yielded the lowest recovery rates, with an average of 39.6 ± 2.2% for low concentrations and 75.4 ± 2.3% for high concentrations. Across the three matrices and two concentrations tested, SEI yielded the lowest average recovery rates (33.5 ± 1.8%), while SEC1 demonstrated the highest average recovery rate (94.8 ± 5.3%). For one-time EC_50_ concentrations, the recovery rates ranged from 4.5 ± 0.5% for SEI in pork pâté extract to 122.7 ± 7.7% for SEC1 in raw milk cheese extract. At the higher toxin concentrations of ten times EC_50_, the recovery rates spanned from 31.7 ± 1.4% for SEI in pork pâté extract to 127.3 ± 8.7% for SEC1 in raw milk cheese extract. Overall, the multiplex SIA for the detection of SEA to SEI was able to recover the spiked toxins within the three- to four-digit pg/mL range tested in the three undiluted food extracts.

## 3. Discussion

In this study, a multiplex SIA for the simultaneous, sensitive, and specific detection and quantification of eight SEs, namely SEA, SEB, SEC, SED, SEE, SEG, SEH, and SEI, was developed and validated, covering multiple variants of the targeted SEs. This approach was designed to reduce the necessity of relying on separate assays and to enhance diagnostic efficiency. Starting from mAb generation and characterisation to establish individual sandwich ELISAs, the method was successfully adapted into a multiplex SIA format and evaluated for its performance on a panel of 145 relevant bacterial strains and on SE-spiked extracts of exemplarily selected food matrices, addressing several critical challenges in SE detection.

While DNA-based methods have been employed for *se* detection, their utility is limited when heat-sensitive bacteria are destroyed, yet the heat-stable toxins remain active [19]. DNA-based methods, such as multiplex PCR and WGS, were originally developed for the simultaneous detection of multiple *se* genes [29,65]. The most commonly used protein-based diagnostic techniques for SEs are reverse passive latex agglutination (RPLA), lateral flow assay (LFA), enzyme-linked fluorescent assay (ELFA), sandwich ELISA, and LC-MS/MS.

Given the large number of SEs and the rarity of monovalent *S. aureus* strains, the simultaneous detection of multiple SEs even from limited sample volumes is crucial in the context of preventing and investigating SFP outbreaks. In addition, detection methods should be highly sensitive with LoDs below 0.06 ng SE/g food, considering their very low effective dose [14,55]. LFAs are a user-friendly diagnostic tool that primarily detects single SEs, including SEA, SEB, SEG, SEH, and SEI [56,57,58]. Commercial multiplex assays, such as RIDASCREEN^®^ SET Total [53] and VIDAS^®^ SET [52], are capable of detecting multiple SE types (SEA–SEE) but lack the ability to differentiate between them and quantify them. In buffer, their sensitivity was described as 31 pg/mL to 500 pg/mL [52,53]. According to the manufacturer, the respective LoDs of the RIDASCREEN^®^ SET Total kit are for 50 pg per millilitre or gram food samples when including a dialytic concentration step, 250 pg/mL (liquid food samples) or 375 pg/mg (solid food samples) without dialysis, and 250 pg/mL for bacterial culture supernatants (without dialysis).

ELISA detection can cover single toxin detection of SEA to SEI with LoDs in the low pg/mL range in buffer, and was also tested for food and bacterial cultures [59,66]. The eight conventional ELISAs presented here, which are specific for SEA to SEI and served as a starting point for the development of the multiplex SIA, are in a comparable sensitivity range with LoDs between 5 and 10 pg/mL.

Other methods, such as multiplex MS, RPLA, and RIDASCREEN^®^ SE A, B, C, D, and E assays [54], permit SE differentiation but are constrained by cross reactivity (CR) of up to 20%, particularly in LFA, RIDASCREEN^®^ sandwich ELISA, and RPLA, which affects accuracy. While the RPLA has an LoD of 1000 pg/mL, LFAs are more sensitive, with LoDs between 6 to 300 pg/mL [56,57,58]. To the best of our knowledge, so far only one multiplex SIA based on the Luminex technology addressing SEs was published, covering SE types A and B, and TSST-1. In this approach, LoDs of 10 pg/mL SEA and 100 pg/mL SEB in buffer were achieved, while LoDs of 10 pg/mL SEA, 1000 pg/mL SEB, and 5 pg/mL TSST-1 in bacterial culture supernatants were obtained [67,68]. Generally, multiplex SIAs allow for the simultaneous detection of multiple targets from minimal sample volumes, thereby enhancing throughput and specificity. In our approach, a 50 µL sample volume was sufficient to detect, differentiate, and quantify eight SEs simultaneously, as compared to 400 µL needed for eight parallel ELISAs.

Compared to these commercial approaches, the here-presented multiplex SIA for the detection of SEA to SEI delivers LoDs between 5 and 15 pg/mL in buffer and reaches LoDs of research-grade ELISAs [59,66] but additionally features the advantage of multiplexing and SE differentiation. Spiking experiments showed that the multiplex SIA was able to analyse undiluted food extracts with high recovery rates for the SEs at concentrations between 144 and 420 pg/mL. In bacterial culture supernatants, the threshold for the multiplex assay ranged between 12.4 and 268 pg/mL, depending on the SE type and variant. The choice of technique depends on the specific requirements of the investigation. While sandwich ELISAs remain a well-established method for the sensitive detection of individual analytes, multiplex SIA offers new possibilities for the comprehensive analysis of complex samples.

The differentiation of SE types in immunoassays, however, represents a significant challenge, largely due to the high degree of sequence homology observed among them. For example, SEA and SEE exhibit approximately 83% amino acid identity, while SEB and SEC demonstrate 69% amino acid identity (Table S2), thereby increasing the probability of CR [12,20,21,22]. To address this issue, highly specific mAbs were generated in this work, exhibiting minimal to no CR to other SE types and remaining compatible with a sandwich format. Of the 20 selected newly generated mAbs, targeting SEA to SEI, 8 demonstrated reactivity beyond their specific targets (Table 1, Figure S3). While the high similarity of SEs is challenging in sandwich ELISA and multiplex SIA [69], it becomes readily apparent in the latter approach, as more than one signal is obtained. Therefore, in the course of assay set up, it was crucial to confirm that the target antigens were specifically bound to the corresponding bead-bound capture mAbs for identification purposes. The introduction of a mAb detection mix, which permits quantification but does not necessarily facilitate differentiation, could potentially impair the interpretation of the results.

The different choices of mAbs for use in the sandwich ELISAs on the one hand and multiplex SIA on the other hand demonstrated considerable compromises between the desired levels of specificity and sensitivity. With regard to the detection of SEA, mAb SEA388 exhibited no CR to SEE, thus establishing it as a promising candidate for use as a capture antibody, while the other two mAbs, SEA2353 and SEA165, showed a CR to the highly related SEE. In light of the epidemiological significance of SEA as a leading cause of SFP outbreaks, the focus was to achieve comprehensive detection of both presented SEA variants. Given the infrequency with which SEE is implicated in SFP outbreaks, the specific detection of this antigen was decided to be of lesser importance. During assay set up, it was found that only the combination of capture mAbs SEA388 plus SEA2353 showed the capacity to detect both SEA variants in combination with biotinylated SEA165. The CR with SEE was observed to be notably low. Nevertheless, a high-level SEE expression could be misidentified as SEA in an SFP outbreak. As the multiplex SIA simultaneously detects several targets, the plausibility of the results reduces the risk of inaccurate conclusions and could be further investigated, for example, by LC-MS/MS.

Another CR to be considered was that of mAb S1001 targeting SEB, which displays a high degree of CR to SEC1. This CR presented a significant challenge, as the resulting signals could not be reliably differentiated between SEB and SEC when using SEC-specific detection antibodies in the multiplex SIA’s detection mixture. Although the usage of this mAb theoretically permitted a lower LoD in the multiplex SIA, as demonstrated in the sandwich ELISA, the inability to interpret the results accurately due to CR rendered it unsuitable for use in the multiplex SIA. In multiplex assays, such CR would compromise both the accuracy of the detection process and the ability to interpret the results, particularly when multiple toxins are present in the sample. Therefore, specificity was given priority, and the mAb S1851, which displays lower CR towards SEC1, was coated onto the magnetic microspheres. This resulted in a slightly higher, but still satisfactory LoD, which ensured the specificity and interpretability of the results.

The sensitivity of the multiplex SIA and sandwich ELISAs was found to be dependent on the selection and affinity of the mAbs employed in their respective configurations. The use of high-affinity mAbs was prioritised during the development process in order to achieve the lowest possible LoDs and EC_50_ values, which are critical for the sensitive detection and quantification of SEs. The multiplex SIA demonstrated an advantage in maintaining comparatively low background signals, which contributed to lower theoretical and experimental LoDs for the majority of targets compared to the sandwich ELISAs (compare Table 3 and Table 4). The detection of SEB represented an exception, due to the utilisation of the capture mAb S1851, which exhibits a lower affinity for SEB in comparison to S1001. The EC_50_ values for SEE and SEI in the multiplex SIA were almost identical to those observed in the sandwich ELISA (compare Table 3 and Table 4), indicating that no loss of sensitivity was incurred for these specific targets. The lowest EC_50_ values in the multiplex SIA were obtained in the detection of SEA, SEC, SEE and SEH, due to the use of high-affinity mAbs. Here, SEH449 was used as the capture mAb for SEH, but in the ELISA as the detection mAb for SEH. For SEA and SEC, the high-affinity combinations applied in the ELISAs were retained, while for SEE, a switch to a higher-affinity capture mAb improved the sensitivity in the multiplex SIA. These findings emphasise the critical role of mAb selection in determining assay performance, striking a balance between sensitivity and specificity to ensure robust detection in both single-analyte and multiplex formats. Especially, the multiplex SIA established in this study addresses several limitations: it enables simultaneous detection and quantification of eight target SEs (including different variants) and exhibits excellent experimentally confirmed LoDs, representing a significant improvement in comparison to commercial assays and MS methods, and is on par with some of the most sensitive sandwich ELISAs and LFAs for SE detection.

The overall performance of the ELISA and multiplex SIA assays and the low coefficients of variation, which are below the acceptable limit of 25% [63], are regarded as being of particular significance with respect to the reliability of the assays. Methods that are sequencing their targets on the DNA level have been able to differentiate different molecular variants of the different SE types, which were recently addressed [29,30]. Due to the variability in *se* sequences, designing primers that bind to all allelic variants and generating antibodies that retain their epitope on the different molecular variants represent a challenge [30]. The detection of protein variants of SEs is a relatively new topic, with only a limited number of studies addressing this issue [59,70]. Along this line, a very recent approach combined immuno-enrichment with an optimised top-down LC-MS/MS procedure to identify several SEA variants on the protein level [70]. While the here-presented multiplex SIA was—due to the pure immunodetection approach—not able to differentiate individual variants of a given SE, it was successfully optimised to detect all different variants present in the panel of strains analysed.

For a better understanding of the role of individual SEs in SFP outbreaks, a correlation analysis between genetic potential and protein expression is required [71]. This was addressed in this study by using whole-genome sequenced bacterial strains in comparison to the detection of SEA to SEI expressed natively in liquid culture. In the current study, the comparison demonstrated that the genetic profile of *sea*, *seb*, *see*, and *seh* perfectly aligned with the protein detection in the multiplex SIA, resulting in 100% accuracy. For *sec*, *sed*, *seg,* and *sei*, the accuracy obtained was 99.3%, 90.3%, 86.9%, and 97.9%, respectively (Table S1). False positive results in the assay could be caused by low protein expression and a signal below the threshold, resulting in the incorrect identification of enterotoxigenic strains. Alternatively, the protein sequence of a given target SE variant may be mutated, resulting in a modification or absence of the mAbs epitope on the specific antigen. Another possibility is that the toxin is not produced at all, making it inaccessible for detection in the immunoassay. In this latter case, a negative multiplex SIA (and ELISA) result would provide the “true answer” as opposed to the genetic *se* profile that highlights the theoretical SE production potential.

In cases where genetic and protein profiles diverged from one another in this study, a detailed examination of the genetic data suggested that the majority of instances involved a diminished concentration of the expressed toxin or, in the case of SED, a lack of production, due to the absence of a promoter [30]. The occurrence of truncated variants of SEs, such as SED and SEG, introduced an additional set of challenges. For example, truncated SED has been observed in *S. aureus*, resulting from the deletion of adenine and the subsequent generation of a premature stop codon [30]. The expression of truncated protein variants of SED is limited, and this could be assumed to be the case for truncated SEG as well, although further intensified research is required to confirm this [30]. Furthermore, it is unclear at the moment if these truncated toxin variants are still biologically active and can play a role in SFP outbreaks at all. The detection of these truncated proteins can be challenging using conventional methods, but the multiplex SIA has proven to be generally successful for truncated SED and SEG. Given that all expressed protein variants of an SE are principally detected with uniform intensity by the applied assay, it can be speculated that individual non-detection is most likely caused by low expression of the toxin, below the LoD of the immunoassay used. For SEG and SEI, which share the same promoter regions, all of the toxins of the *egc* operon were detected at relatively low concentration levels in the current study. These findings are consistent with previous studies that have indicated that *egc* enterotoxins are expressed at lower levels than classical SEs [41,59,72,73].

The potential for non-expressed toxins, as a consequence of a number of factors influencing expression, introduces a degree of complexity when attempting to compare with genetic data. The prediction of expressed proteotoxins with direct protein detection is hindered by the fact that the genetic data cannot be considered a reliable gold standard. In order to verify the non-production of an SE by enterotoxigenic strains, reverse transcriptase PCR would be required as a highly sensitive method [74], and could be used in the future to resolve open questions.

The decreased specificities for SED (90.8%), SEG (87.9%), and SEI (98.5%) in the multiplex SIA compared to the sandwich ELISAs were caused by false positive signals that exceeded the ROC-based threshold, which resulted in the erroneous categorisation as “toxin containing” culture supernatant from strains that are non-enterotoxigenic or negative for a specific SE type according to WGS data. Such false positive results may, on the one hand, be attributed to CR to other SE types. The simultaneous elevation of signals for SEA and SEE or signals for SEB and SEC can be caused by CR based on the mAbs’ specificity, which presents a challenge for interpretation. This is particularly the case given that *sea* and *see* or *seb* and *sec* can be co-encoded. This phenomenon was observed in two instances in the panel of 145 strains analysed here, whereby two *see*-genic strains lacking *sea* yielded positive results in the SEA ELISA. In contrast, this phenomenon was not observed in the multiplex SIA, which is consistent with the reduced CR observed in this assay. On the other hand, false positive results might be caused by background signals originating from non-specific binding of other components in the bacterial culture supernatants, such as protein A, to the capture mAbs, resulting in elevated signals across multiple assays. The impact of non-specific binding was only partially accounted for in the ROC analysis, which was specifically designed to differentiate SE-positive from SE-negative supernatants on a per-target basis, without considering different bead sets corresponding to different SEs.

Other orthogonal assays can offer complementary advantages over multiplex SIA, particularly in terms of detection scope and methodology. For instance, techniques such as LC-MS/MS offer extensive detection capabilities without the need for specific antibodies, making them appropriate for identifying a diverse array of toxins as well as unexpected targets [50]. The sensitivity of LC-MS/MS-based approaches is still somewhat limited compared to pure immunoassays, but recent technical developments, such as timsTOF-MS approaches, are promising and raise the expectation that the current sensitivity gap can be closed in the near future. Similarly, methods such as multiplex PCR can detect genetic markers in lieu of depending on protein-level detection, which can be advantageous in certain contexts, but again highlight only the theoretical potential for SE expression [65]. In any case, multiplex SIA presents certain advantages in contexts where simplicity and high throughput are critical. In contrast to LC-MS/MS, which necessitates intricate sample preparation and a high degree of specialised expertise and instrumentation, multiplex SIA is straightforward to perform and interpret in routine laboratories. Furthermore, its markedly higher throughput renders it optimal for scenarios that require the concurrent analysis of numerous samples or targets, such as outbreak investigations or large-scale screening efforts. The equilibrium of specificity, sensitivity, and operational efficacy positions the multiplex SIA as a highly practical tool for routine and demanding applications, particularly in instances where antibody-based detection is preferred due to its direct correlation with protein expression.

The majority of existing detection assays were primarily tested with spiked matrices, a standard approach that was also employed in this study to assess performance in a monitored setting. The utilisation of spiked matrices permits the precise evaluation of detection capabilities, recovery rates, and sensitivity across disparate food matrices through the introduction of known concentrations of toxins, thereby facilitating the assessment of the assay’s performance under controlled conditions. To evaluate the robustness of the established multiplex SIA for the detection of SEA to SEI in complex matrices, food samples were processed in accordance with the existing ISO standard [55]. However, in the current study, food extracts were spiked (instead of food samples) to evaluate the immunological assay, instead of the entire SE detection method that also covers standardised and already validated food sample extraction and dialysis.

In this context, the multiplex SIA, with its low detection limits and high specificity, is in principle capable of directly handling complex food matrices without dialysis concentration [55]. Assays with reduced sensitivity may benefit from dialysis to concentrate the target and to reduce background noise, but the multiplex SIA’s robust design minimises such interferences—at least for the panel of food matrices tested so far. For these reasons, the final food extracts were spiked, with the aim of assessing the assay’s recovery rather than the entire process efficiency, which includes effects of both food sample preparation and SE detection assay. This spiking approach still demonstrates the potential for matrix effects on toxin detection and availability in complex matrices, as the spiked extracts were incubated for one hour to allow for possible reactions of SEs with the matrix compounds, in accordance with existing recommendations [62].

As anticipated, the higher spiking concentrations (10 times EC_50_) were recovered at a higher rate than the lower toxin concentrations (one-time EC_50_). This was due to the higher availability of the toxins and the reduced impact of matrix interactions. The presence of a high fat concentration, a heterogeneous matrix, a variety of proteins, and other components is likely to present a significant challenge to the successful detection of SEs. Different pH values can impact protein folding, which in turn affects epitope availability and binding of antibodies. The dilution of samples is a common method for reducing the impact of matrix effects. However, this can be challenging to implement in real-world scenarios, as low concentrations of analytes might be diluted below the LoD of the assay. In the case of the multiplex SIA, the spiked extracts of the three selected food matrices could be analysed as undiluted samples and with the individual SEs detectable simultaneously in a single well, requiring only a minimal amount of sample material. The recovery rates, however, exhibited considerable variation, depending on the antigen, its concentration, and the tested matrix, which was anticipated due to the utilisation of undiluted extracts. Overall, the results indicate that the observed recovery rates may be sufficient in SFP outbreaks. The test with the three selected food matrices provides proof of concept. However, due to the vast range of potentially involved foods in SFP, the results of this study are not suitable for general assumptions about the assay’s performance in other food matrices. Therefore, in the future, a more extensive validation study addressing a larger set of relevant foods has to be implemented since each matrix differs and requires special evaluation.

Future research should also focus on enhancing the affinity of mAbs for challenging targets, such as SED, SEG, and SEI, and investigating new mAb combinations to further improve sensitivity. This approach will facilitate the resolution of sensitivity issues while maintaining the reliability of the multiplex SIA across a range of SE targets. In addition, further work should be directed towards the expansion of the range of mAbs, with the objective of targeting additional types of SEs and their integration into the modular multiplex SIA. This would enhance the assay’s versatility and expand its applicability to an even broader range of SEs. Additional validation is necessary for real case scenarios involving the detection of native SEs in authentic samples originating from SFP outbreaks. This will further ensure the robustness and reliability of the assay in the transfer to routine testing and SFP outbreak investigation.

## 4. Conclusions

This study successfully established and validated a multiplex SIA that provides a significant advancement in SE detection on the protein level. By overcoming the challenges associated with cross reactivity, sensitivity, and matrix effects, the multiplex SIA offers a reliable, sensitive, and specific tool for comprehensive SE detection covering the five classical SEs (SEA to SEE), plus additionally SEG, SEH, and SEI. Its ability to simultaneously detect, differentiate, and even quantify multiple toxins in complex matrices from minimal sample volumes highlights its potential for broad applications in food safety assessment, outbreak investigation, and biological threat assessment.

## 5. Materials and Methods

### 5.1. Toxins

Staphylococcal enterotoxins SEA, SEC1, SEC2, SEC3, SED, SEE (Toxin Technology, Sarasota, FL, USA), and SEB (Sigma-Aldrich, Seelze, Germany) were isolated from *S. aureus* culture supernatant, whereas rSED, rSEG, rSEH, and rSEI were recombinantly expressed in *E. coli* under biosafety level 2 containment (Project Number GAA A/Z 40654/3/123/7) and subsequently isolated by a multistep chromatography process, yielding highly pure proteins of 25–29 kDa (rSEs commercially available from toxologics GmbH, Hannover, Germany).

### 5.2. Animal Experiments

The animal experiment for the generation of mAbs using mice was approved and overseen by the State Office for Health and Social Affairs in Berlin (LAGeSo, Berlin, Germany) under the registration numbers H109/03 (date of approval 6 June 2003) and H129/19 (date of approval 3 July 2019). Additional BALB/c mice were approved as donor animals for thymus cells under the registration number T0060/08. Two mouse breeds were used for the generation of monoclonal antibodies: outbred NMRI and inbred BALB/c; both were acquired from Charles River Laboratories (Sulzfeld, Germany). Handling of all animals used in this experiment complied with legal requirements of the German Animal Welfare Act and European legislation for the protection of animals used for scientific purposes (Directive 2010/63/EU). At any point, ad libitum feeding and watering were offered. Mice were used after an acclimatisation period of 10 days.

### 5.3. Generation of Monoclonal Antibodies

Female BALB/c and NMRI mice were immunised at the age of at least 8 weeks. Two to four mice each were initially immunised subcutaneously (s.c.) with SEA (25 µg); SEC (SEC1, SEC2, and SEC3, 10 µg each); rSED (10 µg); SEE (30 µg); rSEG (10 µg); rSEH (10 µg); and rSEI (7 µg) in PBS and TiterMax Gold Adjuvants (Sigma-Aldrich, Munich, Germany) or Gerbu Adjuvants (Gerbu Biotechnik, Heidelberg, Germany). The same amount of antigen was applied at least two times s.c. in four-week time intervals. One week post immunisation, blood samples were taken and tested for whole serum antigen-specific antibody titres in indirect ELISA. Mice with the highest titre against their respective antigen were selected and received the same antigen amount in PBS in daily booster-immunisations intraperitoneally three, two, and one day(s) before fusion according to an established hybridoma protocol [60]. In brief, antibody-producing hybridoma clones were generated by fusing spleen cells with myeloma cells P3-X63-Ag8.653 (American Type Culture Collection, Manassas, VA, USA) at a ratio of 1:2 in polyethylene glycol 1500 (PEG, Roche Diagnostics, Basel, Switzerland). Hybridoma clones were screened for their supernatants containing antibodies, which are able to detect SEs by indirect ELISA and SPR-based methods (screening). Hybridoma clones with supernatants, which were reactive towards their target antigen in the screening, were selected and subcloned at least twice. Clonality was confirmed in flow cytometry measurement by intracellular staining with Cy5-labelled anti-mouse IgG antibodies (Dianova, Hamburg, Germany). Immunoglobulins (IgGs) from hybridoma supernatants were purified by affinity chromatography with a HiTrap MabSelect SuRe column using an ÄKTAexplorer 100 or ÄKTAavant 25 chromatography system (both Cytiva, Freiburg, Germany). Isotypes of mAbs were analysed with an IgG isotyping kit (Mouse Immunoglobulin Panel, SouthernBiotech, Birmingham, AL, USA) according to the manufacturer’s instructions.

### 5.4. Generation of Recombinant Antibodies

Generation of the recombinant mAbs S419, SED333, SED1280, SEE33, SEH449, SEI92, and SEI242 was conducted according to Stern et al. [75]. Briefly, the DNA sequence of named mAbs was determined by using V-region-specific primer libraries and RT-PCR. DNA molecules for antibody light and heavy chains were synthesised by GeneArt (ThermoFisher Scientific, Waltham, MA, USA) and transferred separately into the mammalian expression vector pTT5^®^ (used under licence from the National Research Council of Canada, Ottawa, ON, Canada). All antibodies were expressed as the IgG1 subtype. HEK 293-6E cells (National Research Council of Canada, Ottawa, ON, Canada) were transiently transfected with pTT5^®^ plasmids (1:1 ratio of light and heavy chains) and polyethyleneimine (Polysciences, Warrington, PA, USA). After incubation for 5–7 days at 37 °C and 5% CO_2_ in FreeStyle^TM^ F17 expression medium (Thermo Fisher Scientific, Waltham, MA, USA), the secreted antibodies were purified from the culture supernatants by affinity chromatography with 5 mL HiTrap Protein G HP columns using an ÄKTAexplorer 100 or ÄKTAavant 25 chromatography system (both Cytiva, Freiburg, Germany).

### 5.5. Generation of Liquid Culture Supernatants of Bacterial Strains

Whole-genome sequenced strains of *S. aureus*, other *Staphylococcus* spp., and *Enterococcus faecalis* (Tables S1 and S3; for method description see Section 5.18) were streaked onto Columbia agar containing sheep blood (Thermo Fisher Scientific, Waltham, MA, USA) and cultivated overnight at 37 °C. One colony was picked for each strain and incubated in 5 mL Tryptone Soy Broth (TSB, made in-house) at 37 °C in culture tubes with shaking at 80 rpm in an incubator WNB22 equipped with a shaking device SV1422 (both Memmert, Schwabach, Germany). When the culture reached an optical density at 540 nm (OD_540nm_) of 1–3, the inoculated broth was serially diluted up to 10^−9^ and incubated as described before. After overnight incubation, the lowest growing concentration was photometrically measured and adjusted to an OD_540nm_ of 0.05 in 10 mL of TSB. After incubation for 30 h, the liquid culture was centrifuged for 5 min at 8000× *g* at 4 °C in Megafuge 8R (Thermo Fisher Scientific, Waltham, MA, USA). The supernatant was further filtered through 0.2 µm syringe filters with PVDF membrane (Merck KGaA, Darmstadt, Germany), and the resulting filtrate was used for downstream analysis.

### 5.6. Indirect ELISA

As previously described [76], indirect ELISA was employed to investigate potential cross-reactivity between the utilised mAbs and various SEs. Briefly, the SEs given in Section 5.1, TSST-1 (Toxin Technology, Sarasota, FL, USA), and BSA were individually immobilised at a concentration of 500 ng/mL on MaxiSorb microtiter plates (Thermo Fisher Scientific, Waltham, MA, USA) in PBS with 1 μg/mL BSA overnight at 4 °C. After blocking non-specific binding with 2% skimmed milk powder (Merck KGaA, Darmstadt, Germany) in PBS-T (PBS with 0.1% (*v*/*v*) Tween 20), the mAbs were incubated at a concentration of 10 μg/mL. Detection was performed using a horseradish peroxidase (HRP)-labelled goat anti-mouse IgG (Fcγ) specific antibody (diluted 1:2500; Dianova, Hamburg, Germany) and 3,3′,5,5′-tetramethylbenzidine (TMB, SeramunBlau slow, Seramun Diagnostica GmbH, Heidesee, Germany). After stopping the enzymatic reaction with H_2_SO_4_ (0.25 M), the absorbance was measured at 420 nm referenced to 620 nm by an ELISA reader (Tecan Infinite M Nano and Infinite M200; both Group Ltd., Männedorf, Switzerland).

### 5.7. Surface Plasmon Resonance (SPR) Spectrometry

The Biacore T200 Surface Plasmon Resonance (SPR) device (Cytiva, Freiburg, Germany) was used for measurement of binding kinetics in combination with Sensor Chip CM5 and HBS-EP+ (10 mM HEPES, pH 7.4, 150 nm NaCl, 3 mM EDTA, 0.05% (*v*/*v*) Tween 20) as a running buffer at 25 °C. For measurement of binding kinetics between mAbs with their respective antigen, antibodies were immobilised with coating densities of 300 to 500 resonance units (RUs) onto the sensor chip’s flow cells, using a mouse antibody capture kit (Cytiva, Freiburg, Germany). Flow cell 1 with no bound ligand was used as a reference control, as the analyte was injected into all flow cells. Subsequent injection of 1:3 dilutions of analyte concentrations up to 375 nM in single-cycle format was performed, starting at 4.6 nM for the respective SE. Injection of the analyte endured for 120 s at a flow rate of 30 µL/min. The running buffer was injected for 1800 s. All flow cells were regenerated between cycles by 10 mM glycine (pH 1.7) for 120 s at a flow rate of 10 µL/min. Binding curves were double referenced [77] and fitted to 1:1 Langmuir interaction models using Biacore Evaluation Software v3.2 (Cytiva, Freiburg, Germany). Association rate constant *k*_a_ and dissociation rate constant *k*_d_ were determined, and their ratio calculated as the equilibrium dissociation constant *K*_D_. The control flow cell without mouse antibody capture was used as a reference for all binding curves.

To determine the epitope coverage of the individual anti-SE mAbs, antibodies were immobilised on the flow cells, as described before, and blocked by 100 µg/mL mouse IgG (Jackson ImmunoResearch Europe, Ely, UK) for 120 s and a flow rate of 10 µL/min to avoid unspecific binding on all flow cells. Injection of the respective SE at a concentration of 375 nM on all flow cells for 60 s at a flow rate of 10 µL/min followed. After a dissociation time of 60 s, the second antibody was injected into all flow cells at a concentration of 10 µg/mL and at a flow rate of 10 µL/min. The control flow cell without mouse antibody capture was used as a reference for all binding curves. Recognition of the distinct epitopes on the antigen by two distinct antibodies is indicated as an increased signal after injection of the second antibody.

### 5.8. SDS-PAGE

An amount of 1 µg per antigen was mixed with 3 × Laemmli loading buffer (150 mM Tris-HCl, pH 6.8, 6% (*w*/*v*) SDS, 30% (*v*/*v*) glycerol, 0.25% (*w*/*v*) bromophenol blue) and heated for 10 min at 95 °C. The loaded samples were electrophoretically separated on 10% SDS polyacrylamide gels in accordance with the established standard procedures [78] and stained with Quick Coomassie stain (Protein Ark, Rotherham, UK). The gels were documented with a ChemiDoc imaging system (Bio-Rad Laboratories, Hercules, CA, USA).

### 5.9. Biotinylation of Detection MAbs

Before usage in sandwich ELISA and multiplex SIA (see below), biotinylated mAbs were generated by reacting the mAbs with biotin-N-hydroxysuccinimide (NHS) ester (Sigma-Aldrich, St. Louis, MO, USA) in a molar ratio of 20:1 (mAb:biotin). The biotinylated mAbs were then stored in PBS supplemented with 0.2% (*w*/*v*) bovine serum albumin (BSA; Serva, Heidelberg, Germany) and 0.05% (*w*/*v*) NaN_3_ (Carl Roth, Karlsruhe, Germany).

### 5.10. Antibody-Based Sandwich ELISA

Sandwich ELISAs were conducted as described by Worbs et al., 2015 [79]. MAbs SEA388 and SEA2353 (10 µg/mL each), S1001 (2.5 µg/mL), SEC371 (5 µg/mL), SED1280 (10 µg/mL), SEE33 (5 µg/mL), SEG5 (5 µg/mL), SEH1236 (10 µg/mL), and SEI467 (10 µg/mL) in PBS (50 µL/well) were coated in optimised concentrations on MaxiSorp microtitre plates overnight at 4 °C. After blocking with 200 µL/well casein buffer (0.625% (*w*/*v*) casein, 0.05 M Tris, 0.005% (*w*/*v*) Bronidox, and 0.025% (*v*/*v*) Tween 20 in ddH_2_O) for 1 h at room temperature, the procedure was followed by washing (4 × 300 µL) and by addition of 50 µL of antigen diluted in 0.1% (*w*/*v*) BSA in PBS and incubated for 2 h at room temperature. After a second washing step (4 × 300 µL), 50 µL biotin-labelled detection antibodies diluted in casein buffer were added, and after incubation of 1 h at room temperature, the plate was washed again (4 × 300 µL). After 30 min of incubation with 50 µL of streptavidin-labelled poly HRP (Senova, Weimar, Germany), final washing (8 × 300 µL), development, and measurement were carried out as described for indirect ELISA (see Section 5.6). The antigen standard curve, diluted from 0.3 pg/mL to 100 ng/mL, was analysed using a semi-logarithmic four-parameter model with 1/y^2^ weighting and log-transformed concentration values (GraphPad Prism version 9.1.0 for Windows, GraphPad Software, San Diego, CA, USA).

### 5.11. Coupling of MAbs on MagPlex Beads

The coupling of the mAbs SEA388 and SEA2353 (each 9 µg), S1851 (18 µg), SEC371 (9 µg), SED1280 (18 µg), SEE (18 µg), SEG5 (18 µg), SEH449 (18 µg), or SEI242 (18 µg) was conducted using 1.5 × 10^6^ paramagnetic and fluorescence-coded polystyrene beads (Diasorin, Saluggia, Italy; bead regions 020, 055, 033, 076, 026, 067, 082, and 007, in this order for the individual targets) according to manufacturer’s instructions (Bio-Plex Amine Coupling Kit, Bio-Rad Laboratories, Munich, Germany). The procedure entailed the activation of the carboxyl groups on the beads with EDC/NHS solution, followed by the addition of mAbs to facilitate covalent binding through amine groups. The coupled beads were adjusted to a bead number of 1000 beads/µL in PBS-TBN (0.1% (*w*/*v*) BSA, 0.02% (*w*/*v*) Tween 20, and 0.05% (*v*/*v*) NaN_3_ in PBS).

### 5.12. Multiplex SIA Based on Luminex^®^ Technology

All incubation periods were conducted in conditions that were shielded from light, at room temperature, and on a plate shaker (MTS 2/4 digital microtiter shaker, IKA-Werke, Staufen, Germany) at 600 rpm. A total of 5000 paramagnetic beads per target in 1% (*w*/*v*) BSA in PBS were added per well of a 96-well microplate (Greiner Bio-One, Kremsmünster, Austria) and washed (3 × 300 µL) with PBS-T in a magnet washer (Tecan, Crailsheim, Germany). Subsequently, 50 µL of antigen diluted in 0.1% (*w*/*v*) BSA in PBS was added, and the mixture was incubated for two hours. The antigen standard curve was diluted serially from 0.3 pg/mL to 100 ng/mL. Following the washing procedure (3 × 300 µL), 50 µL of the biotinylated detection mAbs mixture in 1% (*w*/*v*) BSA in PBS was added to each well and incubated for an additional hour, after which the wells were washed (3 × 300 µL). Following a 30 min incubation period with phycoerythrin-coupled streptavidin (SA-PE; Agilent, Santa Clara, CA, USA) in 1% (*w*/*v*) BSA in PBS, the plate was washed (3 × 300 µL) and the beads were resuspended in 125 µL PBS with 0.05% (*w*/*v*) NaN_3_. The fluorescence of the beads and the PE signal were read using a Bio-Plex 200 device with the Bio-Plex Manager software version 6.2 (Bio-Rad Laboratories, Munich, Germany), utilising the high calibration settings. The DD-gates were set at 8,000 and 22,000, with 50 beads per region being measured. The data were fitted to a semi-logarithmic four-parameter model with 1/y weighting (GraphPad Prism version 9.1.0 for Windows, GraphPad Software, San Diego, CA, USA), and the concentration values were transformed using a log function with a base of 10. When establishing the multiplex SIA for the detection of SEA to SEI, the mAb combinations from the single sandwich ELISAs were initially adopted. To accommodate for a high specificity and sensitivity covering all the different SE variants tested, the initial mAb combinations were then optimised to the final set of mAbs used as indicated in Table 4.

### 5.13. Validation of Immunoassays for Buffer

The specificity of both methods—sandwich ELISA and multiplex SIA—was evaluated by examining the response to single toxins in a dilution series (“single-toxin detection mode”, see Figure S6). Reactivity and cross reactivity (CR) per cross-reactive antigen and analyte assay (c_analyte_) were then calculated as the arithmetic mean of 31 and 100 ng/mL (two fixed concentrations in the upper plateau of the standard toxin dilution curves) per antigen (c_cross_) as follows [60]:

$$CR=\frac{c_{analyte}}{c_{cross}}\times 100$$

All single-toxin sandwich ELISAs were validated in accordance with the international consensus protocol for the validation of sandwich ELISAs for protein toxins, published in the recommended operation procedures for CWC-related analysis [62]. The protocol recommends conducting the validation per assay and target on five consecutive days by one operator with the same batch of reagents. It defines the LoD_th_, as the mean of 56 blank (=buffer) measurements plus three times their standard deviation (SD), which are interpolated into a concentration with the standard curves. The LoD_exp_ value was rounded appropriately to subsequent fives for experimental evaluation. For that, ten samples of the LoD_exp_, each analysed in quadruplicate, were analysed. A total of 95% of their measured absorption values had to be above the mean blank plus three times the SD, and 95% of the corresponding concentrations had to be above the LoD_th_. Performance criteria were established using five samples and standard curve dilutions, which were prepared daily in technical duplicates. The inter-assay (CV%_inter_) and intra-assay repeatability (CV%_intra_) were calculated with the means of the daily measurements (mean_inter_) and technical duplicates of one day (mean_intra_), respectively, and with their SD_inter_ and SD_intra_, respectively.

$${CV\%}_{inter}=\frac{{SD}_{inter}}{{mean}_{inter}}\times 100$$

$${CV\%}_{intra}=\frac{{SD}_{intra}}{{mean}_{intra}}\times 100$$

The samples serve as quality controls with predefined toxin concentrations: LoD_th_, lower limit of quantification (LLoQ), two values around the EC_50_ value, and the upper limit of quantification (ULoQ). For accurate quantification, the CV is required to be ≤20%. The overall CV%_inter_(assay) is the mean of all quality controls’ CV%_inter_.

For the validation of the multiplex SIA, a slightly modified protocol was employed, with the following deviations: each assay consisted of all eight bead sets and all eight detection mAbs; all eight SEs were diluted in the standard dilution curve; and four quality control values (LoD_th_, LLoQ, one concentration around the EC_50_ value, and ULoQ) containing either a single SE toxin (“single-toxin detection mode”, Figure S6) or a mixture of SEA to SEI (“multi-toxin detection mode”, Figure S6) were used for the assessment of the CV%. The LoD_th_ was calculated in a manner analogous to that employed in the sandwich ELISA but based on 80 blank measurements. With the same requirements described for ELISA, the LoD_exp_ was evaluated with single toxin detection (two biological replicates, each in 10 technical replicates) in comparison with detection of a mixture of SEA to SEI (three biological replicates, each in 10 technical replicates). Calculation of the CV%_inter_ (assay) included all CV% of the quality controls ≤ 25%.

Recovery of SEs in supernatants was tested in both assays using 145 culture supernatants, 1:10 diluted in 0.1% (*w*/*v*) BSA/PBS, pH 7. The results were further analysed with R (R version 4.1.3). Using the receiver operating curve analysis (pROC package, version 1.18.0 [80]), the optimal thresholds were extracted based on maximising sensitivity and specificity (Youden’s index). The performance parameters, accuracy, sensitivity, and specificity in multiplex SIA and sandwich ELISA per analyte were determined using WGS data as the gold standard. Sensitivity is defined as the proportion of true positive results, specificity as the proportion of correct negative signals, and accuracy as the proportion of correctly detected signals.

$$Sensitivity\%=\frac{n_{true\;positive}}{n_{true\;positive}+n_{false\;negative}}\times 100$$

$$Specificity\%=\frac{n_{true\;negative}}{n_{true\;negative}+n_{false\;positive}}\times 100$$

$$Accuracy\%=\frac{n_{true\;positive}+n_{true\;negative}}{n_{true\;positive}+n_{false\;negative}+n_{true\;negative}+n_{false\;positive}}\times 100$$

### 5.14. First Validation of the Multiplex SIA for Food Matrices

The recovery of SEs by the new multiplex SIA in food sample extracts was investigated using three different typical and exemplarily selected foodstuffs for SFP: raw milk, raw milk cheese, and pork pâté. Prior to spiking, the three food matrices were processed according to the ISO 19020:2017 standard for immunoenzymatic detection of staphylococcal enterotoxins in foodstuffs to obtain representative food extracts [55]. In brief, solid food samples were homogenised with ddH_2_O using a T 18 digital Ultra-Turrax^®^ (KIA, Staufen, Germany). All samples, including the liquid food sample (milk), were then incubated by shaking at room temperature. After acidification (to pH 3.5–4), each sample was centrifuged, and the resulting supernatant neutralised and centrifuged. The neutralised aqueous phase was concentrated by dialysis against PEG solution using a dialysis membrane with a molecular weight cut-off of 6–8 kDa (Spectra/Por^®^ 1, Spectrum Laboratories, Rancho Dominguez, CA, USA). Each concentrated extract was recovered to one-fifth of the weight of the original sample (5 g concentrated extract per 25 g food sample) using PBS (for milk and cheese samples) or ddH_2_O (for pork pâté sample).

The food extracts were spiked with a toxin mixture of SEA to SEI at concentrations of the EC_50_ and ten times the EC_50_ values of each assay, and then applied in five biological replicates. After incubation at 4 °C for 30 min—to allow for possible reactions of SEs with matrix compounds in accordance with existing recommendations [59]—and centrifugation at 12,000× *g* for 5 min in a Megafuge 8R (Thermo Fisher Scientific, Darmstadt, Germany), the spiked samples were measured in the multiplex SIA.

The SE recovery was calculated back in relation to the spiked buffer (0.1% (*w*/*v*) BSA in PBS) sample (lacking food matrix compounds), for which mean concentrations were used:

$$Recovery\%=\frac{mean\;c_{sample}}{mean\;c_{buffer\;control}}\times 100$$

Since the buffer control samples display the optimal situation without matrix interferences, the percentage of the concentration of the buffer control sample compared to the theoretical spiking concentration was set to 100 ± 20%.

### 5.15. Immunoaffinity Enrichment of SEs from Bacterial Culture Supernatants for LC-MS/MS

Monoclonal antibodies (mAbs) directed against SEA, SEB, SEC, SED, SEE, SEG, SEH, and SEI (namely SEA388, S419, SEC371, SED1280, SEE1524, SEG5, SEH449, and SEI242) were immobilised on M-280 tosyl-activated magnetic Dynabeads^®^ (Life Technologies, Oslo Norway) as described by Kull et al. 2010 [61]. Briefly, 250 µL of resuspended Dynabeads^®^ were washed twice with 800 μL of buffer A (0.1 M sodium phosphate buffer, pH 7.4). Each mAb (150 µg) was adjusted in 250 µL of PBS, added separately to the beads and incubated at 37 °C overnight under rotation. The beads were washed twice with 800 μL of buffer B (0.1% (*w*/*v*) BSA in PBS) for 5 min each at 4 °C, resuspended in 800 μL of buffer C (0.2 M Tris containing 0.1% (*w*/*v*) BSA, pH 8.5) and incubated at 37 °C for 4 h. Beads were washed with 800 μL of buffer B and stored in 500 μL of buffer B at 4 °C.

The immuno-enrichment of the different SEs was carried out according to the protocol described at Worbs et al. 2021 with slight modifications [81]. An antibody–bead mix containing 8 μL of each mAb–Dynabeads^®^ was added to 900 μL of SE-containing supernatant of *S. aureus* culture supernatant in a KingFisher^TM^ deep well plate (Thermo Fisher Scientific, Bremen, Germany). The sample was diluted with 100 µL of 10 × buffered saline with 0.5% (*v*/*v*) Tween 20 (10 × PBST). The deep well plate was placed in a KingFisher flex purification system (Thermo Fisher Scientific, Bremen, Germany) for automated bead shaking (2 h) and washing, which included two washes with 1 mL each of PBST (buffered saline with 0.05% (*v*/*v*) Tween 20) followed by one wash with 1 mL of PBS. Beads were eluted into 1 mL of water, removed from the KingFisher flex system, and then separated manually on a DynaMag-2 magnet (Life Technologies, Oslo, Norway). Supernatants were discarded, and the toxin was eluted with 25 μL of 0.1% (*v*/*v*) trifluoroacetic acid (TFA, Merck, Darmstadt, Germany) in Ultra LC-MS-grade water (Carl Roth, Karlsruhe, Germany) for 10 min. Supernatants were transferred to a fresh LoBind Eppendorf tube (Hamburg, Germany) and neutralised with 7 μL of 400 mM NH_4_HCO_3_. The samples were further processed by LC-MS/MS analysis.

### 5.16. Sample Preparation of Immunoaffinity Enriched and Purified/Recombinant SEs for LC-MS/MS

Commercially purchased purified native or recombinant SEs were diluted to 1 µg in 25 µL of 50 mM ammonium bicarbonate buffer with 9% (*v*/*v*) acetonitrile. Purified toxins as well as immunoaffinity enriched SEs were reduced by adding 1.5 µL of 400 mM dithiothreitol (DTT, Sigma-Aldrich, Munich, Germany) for 10 min at 95 °C. Alkylation was carried out by adding 3 μL of 500 mM iodoacetamide (IAA, Sigma-Aldrich, Munich, Germany) and incubating for 30 min at 37 °C. Tryptic digest was performed by adding 5 μL of 0.02 µg/µL proteomics-grade trypsin solution (Sigma-Aldrich, Munich, Germany) and incubating overnight at 37 °C. Trypsin reaction was stopped by adding 4 μL of 10% (*v*/*v*) trifluoroacetic acid (TFA, Merck, Darmstadt, Germany). The obtained peptides of commercially purchased SEs and recombinant SEs were desalted and purified by C18 ZipTip (Merck, Darmstadt, Germany) according to the manufacturer’s protocol. ZipTip eluates were dried in a speedvac concentrator and resuspended in 15 μL of 0.1% (*v*/*v*) formic acid (Thermo Scientific, Bremen, Germany). The concentration of resulting peptides was determined by absorbance measurement at 280 nm in a NanoPhotometer^®^ NP80 (Implen, Munich, Germany). The samples were further processed by LC-MS/MS analysis.

### 5.17. Mass Spectrometry

Peptides were analysed on an Evosep One liquid chromatography system (Odense, Denmark) coupled online via CaptiveSpray source to a timsTOF mass spectrometer (timsTOF HT, Bruker Daltonics, Bremen, Germany). Peptide solution (50 ng) was loaded on an Evotip pure (Evosep) according to the manufacturer’s protocol. Peptides were separated on a performance column (ReproSil Saphir C18, 8 cm × 150 µM, 1.5 µm, Evosep, Odense, Denmark) with an Evosep 100 sample per day gradient method (100 SPD). The temperature of the LC column was set to 40 °C with a column toaster (Bruker Daltonics). Peptides were ionised by electrospray with a CaptiveSpray emitter (20 µm i.d., Bruker Daltonics) at a capillary voltage of 1450 V. The mass spectrometer was operated in a data-dependent acquisition mode using parallel accumulation–serial fragmentation (ddaPASEF^®^) technology and the following settings were applied: *m*/*z* range of 100–1700; ion mobility (IM) range of 0.65–1.35 Vs/cm^2^; precursors were selected for fragmentation above an intensity threshold of 1000 arbitrary units (a.u.); and the target intensity was set to 20,000 a.u. Fragmented precursors were actively excluded for 0.20 min. The collision energy was decreased as a function of the IM from 59 eV at 1/K_0_ = 1.6 Vs/cm to 20 eV at 1/K_0_ = 0.6 Vs/cm. One cycle consisted of four PASEF ramps. Mass data were processed by MSConvert 3.0 [82] and MASCOT server 2.4 software (Matrix Science Ltd., London, UK). The database search was conducted with carbamidomethyl (C) as fixed and oxidation (M) as variable modifications. Protein mass was unrestricted, the peptide mass tolerance was set to ± 10 ppm, and the fragment mass tolerance was set to ±0.02 Da.

### 5.18. DNA Sequencing and Sequence Analysis

*Staphylococcus* spp. strains were inoculated in 5 mL brain–heart-infusion broth and aerobically incubated at 37 °C for 24 h. DNA of 1 mL culture was extracted using the Qiagen DNeasy Blood and Tissue Kit (Qiagen, Hilden, Germany) according to the manufacturer’s protocol, which was modified by adding 10 µL lysostaphin (0.1 mg/mL; Sigma Aldrich, Taufkirchen, Germany) to the lysis buffer.

The DNA library was prepared using an Illumina Nextera XT DNA library preparation or Nextera DNA Flex Library Prep kits (Illumina Inc., San Diego, CA, USA), and the 150 bp paired-end sequencing run was performed using, for RKI strains, either an Illumina MiSeq or a HiSeq instrument with the 2 × 300 MiSeq v3 or the 2 × 250 HiSeq Rapid SBS v2 reagent kit (Illumina Inc., San Diego, CA, USA), respectively, and for BfR strains, a NextSeq 500 instrument with 2 × 150 NextSeq 500/550 Mid Output v2.5 kit (Illumina Inc., San Diego, CA, USA).

Trimming of raw Illumina reads of the RKI strains was carried out with Trimmomatic followed by de novo assembling using SPAdes within SeqSphere+ v.7.04 (Ridom, Münster, Germany). Quality control of raw reads was conducted using FastQC. For BfR strains, raw Illumina reads were trimmed and *de novo* assembled using a SPAdes algorithm with the in-house-developed AQUAMIS pipeline [83].

Bacterial characterisation was conducted with the in-house-developed Bakcharak pipeline (https://gitlab.com/bfr_bioinformatics/bakcharak, accessed on 8 February 2023) using the VirulenceFinder 2.0 (Center for Genomic Epidemiology, database version: 2022-12-02, https://cge.food.dtu.dk/services/VirulenceFinder/, accessed on 8 February 2023) [84,85] for determination of staphylococcal enterotoxin genes and variants [86]. Reference sequences of the *se* gene variants of the VirulenceFinder database considered in this study can be accessed with the following accession IDs (the numbering of the gene variants can differ from the numbering in the database): *sea.v1* (AP009324.1), *sea.v2* (CP010526.1), *seb.v1* (CP007539.1), *seb.v2* (AB716349.1), *seb.v3* (AB716351.1), *seb.v4* (AB716352.1), *sec.v1* (AB084256.1), *sec.v2* (KF386012.1), *sec.v3* (KF729631.1), *sec.v4* (M28364.1), *sed* (M28521.1), *see* (M21319.1), *seg.v1* (CP001844.2), *seg.v2* (CP002388.1), *seg.v3* (AJ938182.1), *seh.v1* (BX571857.1), *seh.v2* (AY345144.1), *sei.v1* (BA000018.3), *sei.v2* (AJ938182.1), and *sei.v3* (CP002388.1). In regard to the subtypes of SEC, SEC1 is encoded by *sec.v2* and *sec.v3*, SEC2 by *sec.v4*, and SEC3 by *sec.v1*.

### 5.19. Graphics and Illustrations

For graphics and illustrations, where indicated, BioRender was used under the license indicated under each figure. No GenAI were used for this purpose.

## Figures and Tables

**Table 1:** Specificity and affinity for different SE types of in-house mAbs generated and implemented in this study.

Antigen	Epitope ^c^	Clone	Indirect ELISA ^a^										SPR Measurements ^b^		
			SEA	SEB	SEC1	SEC2	SEC3	SED	SEE	SEG	SEH	SEI	*k*_a_ (M^−1^ s^−1^)	*k*_d_ (s^−1^)	Affinity *K*_D_ (M)
SEA	1	SEA165	++++	−	−	−	−	+	++	−	−	−	3.9 ± 0.0 × 10^5^	4.0 ± 0.0 × 10^−4^	1.0 ± 0.0 × 10^−9^
	2	SEA388	+++	−	−	−	−	−	−	−	−	−	8.3 ± 0.0 × 10^5^	7.8 ± 0.0 × 10^−4^	9.4 ± 0.0 × 10^−10^
	3	SEA2353	++++	−	−	−	−	−	+++	−	−	−	8.7 ± 0.1 × 10^4^	6.0 ± 0.0 × 10^−4^	6.9 ± 0.1 × 10^−9^
SEB	1	S419 [60,61]	−	++++	+	−	−	−	−	−	−	−	4.3 ± 0.0 × 10^5^	1.9 ± 0.0 × 10^−5^	4.6 ± 0.0 × 10^−11^
	2	S1001 [60,61]	−	++++	++++	−	−	−	−	−	−	−	7.3 ± 0.0 × 10^5^	4.1 ± 0.3 × 10^−6^	5.6 ± 0.4 × 10^−12^
	3	S1851 [61]	−	++++	+	−	−	−	−	−	−	−	6.3 ± 0.0 × 10^5^	1.4 ± 0.0 × 10^−4^	2.2 ± 0.0 × 10^−10^
SEC	1	SEC290	−	−	++++	++++	++++	−	−	−	−	−	9.2 ± 0.1 × 10^5^	1.1 ± 0.8 × 10^−5^	1.2 ± 0.8 × 10^−11^
	2	SEC371	−	−	++++	+++	++++	−	−	−	−	−	5.0 ± 0.0 × 10^5^	7.5 ± 0.1 × 10^−5^	1.5 ± 0.0 × 10^−10^
SED	1	SED9	−	−	−	−	−	+	−	−	−	−	7.3 ± 0.0 × 10^5^	3.6 ± 0.0 × 10^−3^	4.9 ± 0.0 × 10^−9^
	2	SED333	−	−	−	−	−	+	−	−	−	−	1.2 ± 0.0 × 10^5^	6.0 ± 0.1 × 10^−4^	4.8 ± 0.0 × 10^−9^
	3	SED1280	−	−	−	−	−	++++	−	−	−	−	2.2 ± 0.0 × 10^5^	9.9 ± 0.1 × 10^−5^	4.5 ± 0.0 × 10^−10^
SEE	1	SEE33	−	−	−	−	−	−	++++	−	−	+	7.8 ± 0.0 × 10^4^	1.6 ± 0.0 × 10^−4^	2.1 ±0.0 × 10^−9^
	2	SEE1524	−	−	−	−	−	−	++++	−	−	−	4.1 ± 0.0 × 10^5^	4.0 ± 0.0 × 10^−5^	9.7 ± 0.0 × 10^−11^
SEG	1	SEG5	−	−	−	−	−	−	−	++++	+	−	3.9 ± 0.0 × 10^5^	1.9 ± 0.0 × 10^−4^	4.8 ± 0.0 × 10^−10^
	2	SEG158	−	−	−	−	−	−	−	++++	−	−	3.3 ± 0.0 × 10^5^	2.6 ± 0.1 × 10^−4^	7.9 ± 0.1 × 10^−10^
SEH	1	SEH449	−	−	−	−	−	−	−	−	++++	−	7.2 ± 0.0 × 10^5^	9.4 ± 0.9 × 10^−6^	1.3 ± 0.1 × 10^−11^
	2	SEH1236	−	+	−	−	−	−	−	−	++++	−	1.5 ± 0.0 × 10^5^	3.1 ± 0.0 × 10^−4^	2.0 ± 0.0 × 10^−9^
SEI	1	SEI92	−	−	−	−	−	−	−	−	−	++++	2.0 ± 0.0 × 10^5^	1.7 ± 0.0 × 10^−4^	8.2 ± 0.0 × 10^−10^
	2	SEI242	−	−	−	−	−	−	−	−	−	++++	2.9 ± 0.0 × 10^5^	5.7 ± 0.0 × 10^−5^	2.0 ± 0.0 × 10^−10^
	2	SEI467	−	−	−	−	−	−	−	−	−	++++	1.2 ± 0.0 × 10^5^	2.2 ± 0.0 × 10^−4^	1.8 ± 0.0 × 10^−9^

^a^ The signal strengths were compared to the antibody that produced the highest signal for the given toxin and are presented on a relative scale: (−) no signal; (+) less than 25%; (++) 25–50%; (+++) 50–75%; and (++++) greater than 75% relative signal intensity (Figure S3). ^b^ Binding parameters (association rate constant *k*_a_, dissociation rate constant *k*_d_, and equilibrium dissociation constant *K*_D_ indicating the affinity) were determined by surface plasmon resonance (SPR) measurements. For binding curves, see Figure S4. ^c^ According to an epitope binding approach using SPR (see Section 5.7).

**Table 2:** Reactivities and cross-reactivities (CRs) in sandwich ELISA and multiplex SIA in % (given, if ≥0.1%).

		Target of ELISA ^1^/Multiplex SIA ^2^							
Toxin tested		SEA	SEB ^3^	SEC	SED ^3^	SEE ^3^	SEG	SEH ^3^	SEI ^3^
	SEA	100/100	-	-	-	-	-	-	-
	SEB	-	100/100	-	-	-	-	-	-
	SEC1	-	0.2/0.0	100/100	-	-	-	-	-
	SED	-	-	-	100/100	-	-	-	-
	SEE	1.7/0.6	-	-	-	100/100	-	-	-
	SEG	-	-	-	-	-	100/100	-	-
	SEH	-	-	-	-	-	-	100/100	-
	SEI	-	-	-	-	-	-	-	100/100

^1^ ELISA results are shown without an underscore. ^2^ Multiplex SIA results are underscored. ^3^ Changes in mAb combination between ELISA and multiplex SIA. Reactivities and CRs are expressed as a percentage of the apparent analyte concentration relative to the concentration of the cross-reactive antigen.

**Table 3:** Assay performance for sandwich ELISAs specific for SEA to SEI to determine the limits of detection (LoD) in buffer, the lower and upper limits of quantification (LLoQ, ULoQ), as well as intra- and inter-coefficients of variation (CV%intra, CV%inter) determined at half-maximal effective concentrations (EC_50_).

Antigen	Capture mAb	Detection mAb	LoD_th_ ^a^ [pg/mL]	LoD_exp_ ^b^ [pg/mL]	LLoQ [pg/mL]	EC_50_ [pg/mL]	ULoQ [pg/mL]	CV%_intra_	CV%_inter_
SEA	SEA388 + SEA2353	SEA165	1.2	5	6	54 ± 6	200	7.7	8.0
SEB	S1001	S419	0.4	5	15	35 ± 1	100	5.9	4.1
SEC1	SEC371	SEC290	1	5	15	29 ± 4	90	7.8	9.5
SED	SED1280	SED333	6	10	50	222 ± 27	700	5.2	6.6
SEE	SEE33	SEE1524	1.8	5	7	85 ± 22	300	7.9	8.2
rSEG	SEG5	SEG158	2.4	5	10	81 ± 4	190	9.0	9.0
rSEH	SEH1236	SEH449	1	5	10	61 ± 5	240	6.3	7.2
rSEI	SEI467	SEI92	5	10	30	278 ± 41	800	4.9	9.8

^a^ LoD_th_ = theoretically calculated detection limit. ^b^ LoD_exp_ = experimentally confirmed detection limit. The data are derived from *n* = 5 experiments and the standard curves depicted in Figure 1.

**Table 4:** Assay performance for multiplex SIA specific for SEA to SEI to determine limits of detection (LoD), lower and upper limits of quantification (LLoQ, ULoQ), as well as intra- and inter-coefficients of variability (CV%_intra,_ CV%_inter_) determined at half-maximal effective concentrations (EC_50_).

Antigen	Capture mAb	Detection mAb	LoD_th_ ^a^ [pg/mL]	LoD_exp_ ^b^ [pg/mL]	LLoQ [pg/mL]	EC_50_ [pg/mL]	ULoQ [pg/mL]	CV%_intra_	CV%_inter_
SEA	SEA388 + SEA2353 *	SEA165	<1	5	75	145 ± 8	300	5.4	12.3
SEB	S1851	S419	3.2	15	40	240 ± 16	600	7.5	12.8
SEC1	SEC371	SEC290	<1	5	6	144 ± 3	400	5.1	9.1
SED	SED1280	SED9	4.1	5	200	420 ± 38	900	8.3	10.9
SEE	SEE1524	SEE33	<1	5	40	130 ± 10	250	6.1	14.6
rSEG	SEG5	SEG158	<1	5	110	241 ± 24	650	15.8	12.4
rSEH	SEH449	SEH1236	<1	5	15	162 ± 10	420	6.8	12.7
rSEI	SEI242	SEI92	<1	5	170	280 ± 14	950	4.9	11.9

* Both anti-SEA mAbs were coated onto one bead region simultaneously. ^a^ LoD_th_ = theoretically calculated detection limit. ^b^ LoD_exp_ = experimentally confirmed detection limit applying the “multi-toxin detection mode”. Underscored antibodies differed in the multiplex SIA from the mAb combination used in the corresponding sandwich ELISA. The data are derived from *n* = 5 experiments, and the standard curves are depicted in Figure 1.

## Data Availability

The original contributions presented in this study are included in the article and Supplementary Materials. Further inquiries can be directed to the corresponding author.

## Supplementary Materials

The following supporting information can be downloaded at https://www.mdpi.com/article/10.3390/toxins17060265/s1. Figure S1: Purity of staphylococcal enterotoxins used as antigens; Figure S2: Identity and amino acid sequence coverage of purified native and recombinant *Staphylococcus aureus* enterotoxins used as antigens for immunisation and/or hybridoma screening according to tandem mass spectrometry analysis; Figure S3: Specificity of novel monoclonal antibodies (mAbs) used for setting up sandwich ELISAs and multiplex suspension immunoassay (SIA); Figure S4: Binding kinetics of mAbs produced and applied in this study as assessed by surface plasmon resonance spectroscopy (SPR); Figure S5: Determination of the detection limits for the eight sandwich ELISAs specific for SEA to SEI; Figure S6: Set up of ELISA and multiplex SIA detecting one or multiple SEs; Figure S7: Determination of the detection limit of SEA to SEI in the multiplex SIA; Table S1: Overview of whole-genome sequenced bacterial strains and the results of SE analysis via multiplex SIA in their liquid culture supernatants; Figure S8: Correlation matrix for simultaneous enterotoxin gene encoding; Table S2: Protein alignment of all SEA-SEI incl. variants in presented strains; Figure S9: Sensitivity and specificity of the multiplex SIA detecting SEA to SEI; Figure S10: Overview of the estimated toxin concentration per SE type and variant in all supernatants derived from *sea*- to *sei*-genic strains measured by multiplex SIA; Figure S11: Sensitivity and specificity of the individual sandwich ELISAs detecting SEA to SEI; Table S3: Overview of whole-genome sequenced bacterial strains and the results of SE analysis via eight sandwich ELISAs in their liquid culture supernatants; Figure S12: Performance parameters of conventional sandwich ELISAs for the detection of native toxins and variants in bacterial culture supernatants; Figure S13: Amino acid sequence coverage of different SEs from bacterial culture supernatants after immunoaffinity enrichment, tryptic digest, and tandem mass spectrometry analysis (LC-MS/MS); Figure S14: Estimation of matrix effects in the multiplex SIA; Table S4: Overview of recoveries ± SD in % of toxins spiked to food extracts for multiplex SIA.
