# Supplementary material for: Highly Sensitive Suspension Immunoassay for Multiplex Detection, Differentiation, and Quantification of Eight Staphylococcus aureus Enterotoxins (SEA to SEI)
Source: Toxins (Basel). 2025 May 24;17(6):265. doi: 10.3390/toxins17060265 (PMC12197356; doi:10.3390/toxins17060265)
Supplement: Supplementary file 1 [file toxins-17-00265-s001.zip › toxins-3580151-supplementary.pdf]

# Supplementary Materials: Highly Sensitive Suspension Immunoassay for Multiplex Detection, Differentiation, and Quantification of Eight *Staphylococcus aureus* Enterotoxins (SEA to SEI)

**Paulin Dettmann, Martin Skiba, Daniel Stern, Jasmin Weisemann, Hans Werner Mages, Nadja Krez, Martin B. Dorner, Sara Schaarschmidt, Marc A. Avondet, Marcus Fulde, Andreas Rummel, Birgit Strommenger, Sven Maurischat, and Brigitte G. Dorner**

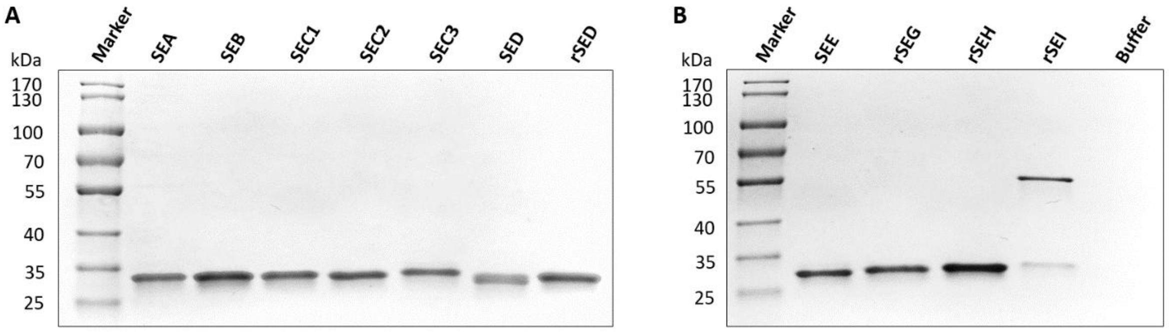


**Figure S1.** Purity of staphylococcal enterotoxins used as antigens. A) Native SEA, SEB, SEC1, SEC2, SEC3, SED, and recombinantly generated SED and B) native SEE and recombinantly generated SEG, SEH, and SEI were separated by SDS-PAGE to evaluate their purity. PBS was used as negative control. 1 µg per antigen was mixed with 3 × Laemmli loading buffer and heated. Samples were separated by 10% sodium dodecyl sulphate polyacrylamide gel electrophoresis (SDS-PAGE) according to standard procedures and stained with Quick Coomassie stain (Protein Ark, Rotherham, UK). Gels were documented using a ChemiDoc imaging system (Bio-Rad Laboratories, Hercules, CA, USA).

**SEA**
UniProt P0A0L1, Enterotoxin type A
Protein sequence coverage: 100%

1 MKKTAFTLLL FIALTLTTSP LVNGSEKSEE INEKDLRKKS ELQGTALGNL
51 KQIYYYNEKA KTENKESHDQ FLQHTILFKG FFTDHSWYND LLVDFDSKDI
101 VDKYKGKKVD LYGAYYGYQC AGGTPNKTAC MYGGVTLHDN NRLTEEKKVP
151 INLWLDGKQN TVPLETVKTN KKNVTVQELD LQARRYLQEK YNLYNSDVFD
201 GKVQRGLIVF HTSTEPSVNY DLFGAQGQYS NTLLRIYRDN KTINSENMHI
251 DIYLYTS

**SEB**
UniProt P01552, Enterotoxin type B
Protein sequence coverage: 98%

1 MYKRLFISHV ILIFALILVI STPNVLAESQ PDPKPDELHK SSKFTGLMEN
51 MKVLYDDNHV SAINVKSIDQ FLYFDLIYSI KDTKLGNYDN VRVEFKNKDL
101 ADKYKDKYVD VFGANYYYQC YFSKKTNDIN SHQTDKRKTC MYGGVTEHNG
151 NQLDKYRSIT VRVFEDGKNL LSFDVQTNKK KVTAQELDYL TRHYLVKNKK
201 LYEFNNSPYE TGYIKFIENE NSFWYDMMPA PGDKFDQSKY LMMYNDNKMV
251 DSKDVKIEVY LTTKKK

**SEC1**
UniProt P01553, Enterotoxin type C-1
Protein sequence coverage: 100%

1 MNKSRFISCV ILIFALILVL FTPNVLAESQ PDPTPDELHK ASKFTGLMEN
51 MKVLYDDHYV SATKVKSVDK FLAHDLIYNI SDKKLKNYDK VKTELLNEGL
101 AKKYKDEVVD VYGSNYYVNC YFSSKDNVGK VTGGKTCMYG GITKHEGNHF
151 DNGNLQNVLI RVYENKRNTI SFEVQTDKKS VTAQELDIKA RNFLINKKNL
201 YEFNSSPYET GYIKFIENNG NTFWYDMMPA PGDKFDQSKY LMMYNDNKTV
251 DSKSVKIEVH LTTKNG

**SEC2**
UniProt P34071, Enterotoxin type C-2
Protein sequence coverage: 100%

1 MNKSRFISCV ILIFALILVL FTPNVLAESQ PDPTPDELHK SSEFTGTMGN
51 MKYLYDDHYV SATKVMSVDK FLAHDLIYNI SDKKLKNYDK VKTELLNEDL
101 AKKYKDEVVD VYGSNYYVNC YFSSKDNVGK VTGGKTCMYG GITKHEGNHF
151 DNGNLQNVLI RVYENKRNTI SFEVQTDKKS VTAQELDIKA RNFLINKKNL
201 YEFNSSPYET GYIKFIENNG NTFWYDMMPA PGDKFDQSKY LMMYNDNKTV
251 DSKSVKIEVH LTTKNG

**SEC3**
UniProt P0A0L3, Enterotoxin type C-3
Protein sequence coverage: 98%

1 MYKRLFISRV ILIFALILVI STPNVLAESQ PDPMPDDLHK SSEFTGTMGN
51 MKYLYDDHYV SATKVKSVDK FLAHDLIYNI SDKKLKNYDK VKTELLNEDL
101 AKKYKDEVVD VYGSNYYVNC YFSSKDNVGK VTGGKTCMYG GITKHEGNHF
151 DNGNLQNVLV RVYENKRNTI SFEVQTDKKS VTAQELDIKA RNFLINKKNL
201 YEFNSSPYET GYIKFIENNG NTFWYDMMPA PGDKFDQSKY LMMYNDNKTV
251 DSKSVKIEVH LTTKNG

**SED**
UniProt P20723, Enterotoxin type D
Protein sequence coverage: 87%

```
  1 MKKFNILIAL LFFTSLVISP LNVKANENID SVKEKELHKK SELSSTALNN
 51 MKHSYADKNP IIGENKSTGD QFLENTLLYK KFFTDLINFE DLLINFNSKE
101 MAQHFKSKNV DVYPIRYSIN CYGGEIDRTA CTYGGVTPHE GNKLKERKKI
151 PINLWINGVQ KEVSLDKVQT DKKNVTVQEL DAQARRYLQK DLKLYNNDTL
201 GGKIQRGKIE FDSSDGSKVS YDLFDVKGDF PEKQLRIYSD NKTLSTEHLH
251 IDIYLYEK
```

**rSED**
Recombinant enterotoxin type D, Protein-ID: ANJ16444
Protein sequence coverage: 97%

```
 26 NENIDSVKEK ELHKKSELSS TALNNMKHSY ADKNPIIGEN KSTGDQFLEN
 76 TLLYKKFFTD LINFEDLLIN FNSKEMAQHF KSKNVDVYAI RYSINCYGGE
126 IDKTACTYGG VTPHEGNKLK ERKKIPINLW INGVQKEVSL DKVQTDKKNV
176 TVQELDAQAR RYLQKDLKLY NNDTLGGKIQ RGKIEFDSSD GSKVSYDLFD
226 VKGDFPEKQL RIYSDNKTLS TEHLHIDIYL YEKGDLVPR
```

**SEE**
UniProt P12993, Enterotoxin type E
Protein sequence coverage: 96%

```
  1 MKKTAFILLL FIALTLTTSP LVNGSEKSEE INEKDLRKKS ELQRNALSNL
 51 RQIYYYNEKA ITENKESDDQ FLENTLLFKG FFTGHPWYND LLVDLGSKDA
101 TNKYKGKKVD LYGAYYGYQC AGGTPNKTAC MYGGVTLHDN NRLTEEKKVP
151 INLWIDGKQT TVPIDKVKTS KKEVTVQELD LQARHYLHGK FGLYNSDSFG
201 GKVQRGLIVF HSSEGSTVSY DLFDAQGQYP DTLLRIYRDN KTINSENLHI
251 DLYLYTT
```

**rSEG**
Recombinant enterotoxin type G, Protein-ID: AAC26660
Protein sequence coverage: 100%

```
 26 QPDPKLDELN KVSDYKNNKG TMGNVMNLYT SPPVEGRGVI NSRQFLSHDL
 71 IFPIEYKSYN EVKTELENTE LANNYKDKKV DIFGVPYFYT CIIPKSEPDI
126 NQNFGGCCMY GGLTFNSSEN ERDKLITVQV TIDNRQSLGF TITTNKNMVT
176 IQELDYKARH WLTKEKKLYE FDGSAFESGY IKFTEKNNTS FWFDLFPKKE
226 LVPFVPYKFL NIYGDNKVVD SKSIKMEVFL NTHGDLVPR
```

**rSEH**
Recombinant enterotoxin type H, Protein-ID: WP_154289210
Protein sequence coverage: 100%

```
 25 EDLHDKSELT DLALANAYGQ YNHPFIKENI KSDEISGEKD LIFRNQGDSG
 75 NDLRVKFATA DLAQKFKNKN VDIYGASFYY KCEKISENIS ECLYGGTTLN
125 SEKLAQERAI GANVWVDGIQ KETELIRTNK KNVTLQELDI KIRKILSDKY
175 KIYYKDSEIS KGLIEFDMKT PRDYSFDIYD LKGENDYEID KIYEDNKTLK
225 SDDISHIDVN LYTKKKVGDL VPR
```

**rSEI**

Recombinant enterotoxin type I, Protein-ID: BAB42914

Protein sequence coverage: 86%

```
 25 QGDIGVGNLR NFYTKHDYID LKGVTDKNLP IANQLEFSTG TNDLISESNN
 75 WDEISKFKGK KLDIFGIDYN GPCKSKYMYG GATLSGQYLN SARKIPINLW
125 VNGKHKTIST DKIATNKKLV TAQEIDVKLR RYLQEEYNIY GHNNTGKGKE
175 YGYKSKFYSG FNNGKVLFHL NNEKSFSYDL FYTGDGLPVS FLKIYEDNKI
225 IESEKFHLDV EISYVDSNGD LVPR
```

**Figure S2.** Identity and amino acid sequence coverage of purified native and recombinant *Staphylococcus aureus* enterotoxins used as antigens for immunisation and/or hybridoma screening according to tandem mass spectrometry analysis. Amino acid sequence coverage of different native and recombinant SEs after reduction, alkylation, tryptic digest, and tandem mass spectrometry analysis (LC-MS/MS with data-dependent acquisition mode using parallel accumulation-serial fragmentation). Identified sequences are shown in red and bold; signal peptides that are not present in the mature proteins and therefore cannot be identified by MS are marked in yellow, artificially introduced amino acids (GDLVPR) at the C-terminus of recombinant enterotoxins (rSED, rSEG, rSEH and rSEI) are underlined. Protein sequence coverage is calculated without signal peptide.

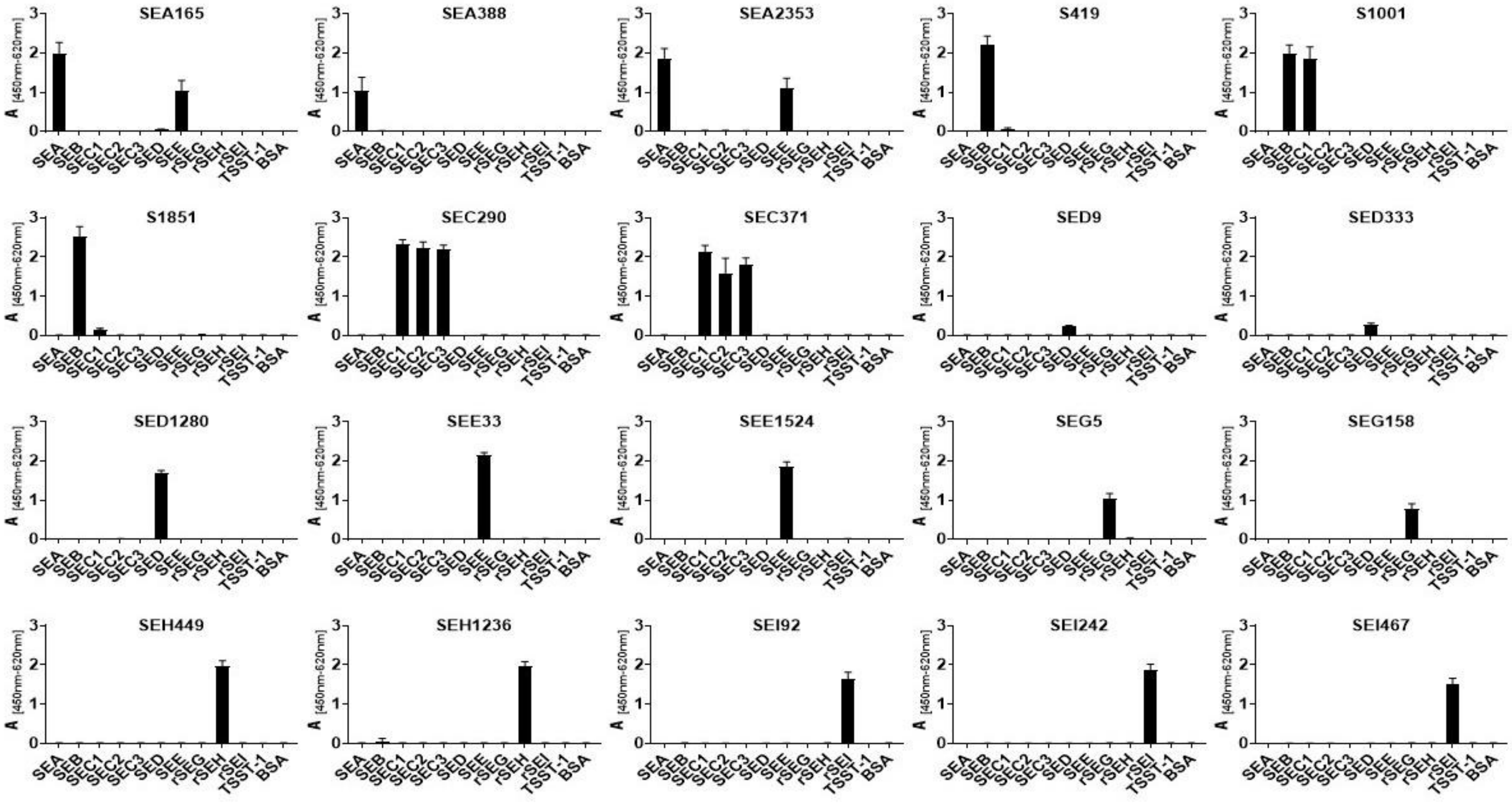


**Figure S3.** Specificity of novel monoclonal antibodies (mAbs) used for setting up sandwich ELISAs and multiplex suspension immunoassay (SIA). All mAbs were obtained after immunising mice with the respective antigens. The mAbs were tested by indirect ELISA on different enterotoxin types (A, B, C1, C2, C3, D, E, G, H, and I), TSST-1, or BSA. Shown are the results of an indirect ELISA where the immobilised antigens (500 µg/mL) on a microtiter plate were tested for binding against mAbs SEA165, SEA388, and SEA2353 generated against SEA; S419 [60], S1001 [60], and S1851 [61] against SEB; SEC290 and SEC371 against SEC; SED9, SED333, and SED1280 against SED; SEE33 and SEE1524 against SEE; SEG5 and SEG158 against SEG; SEH449 and SEH1236 against SEH; as well as SEI92, SEI242, and SEI467 against SEI. Anti-mouse peroxidase-labelled detection Ab was used for the detection of the signals.

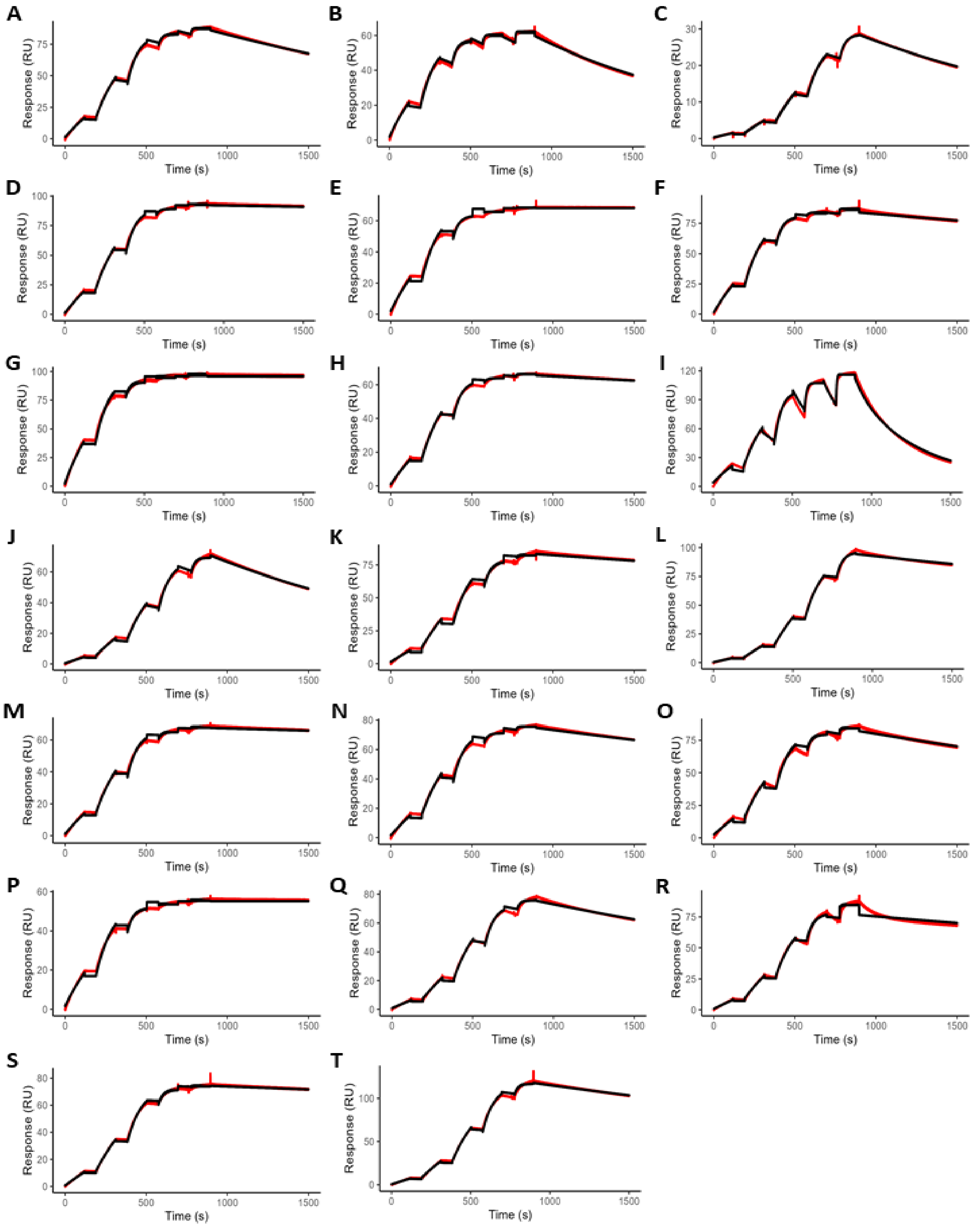


**Figure S4.** Binding kinetics of mAbs produced and applied in this study as assessed by surface plasmon resonance spectroscopy (SPR). The different mAbs were immobilised on CM5 sensor chips: A) SEA165, B) SEA388, C) SEA2353, D) S419, E) S1001, F) S1851, G) SEC290, H) SEC371, I) SED9, J) SED333, K) SED1280, L) SEE33, M) SEE1524, N) SEG5, O) SEG158, P) SEH449, Q) SEH1236, R) SEI92, S) SEI242, or T) SEI467. The corresponding specific analytes were injected in 1:3 dilutions up to 375 nM: A) to C) SEA; D) to F) SEB; G) to H) SEC1; I) to K) SED; L) to M) SEE; N) to O) rSEG; P) to Q) rSEH; and R) to T) rSEI. Binding kinetics of the mAbs were determined by SPR. Double referenced measured binding curves are shown in red, and fitted to a 1:1 Langmuir model curves are shown in black.

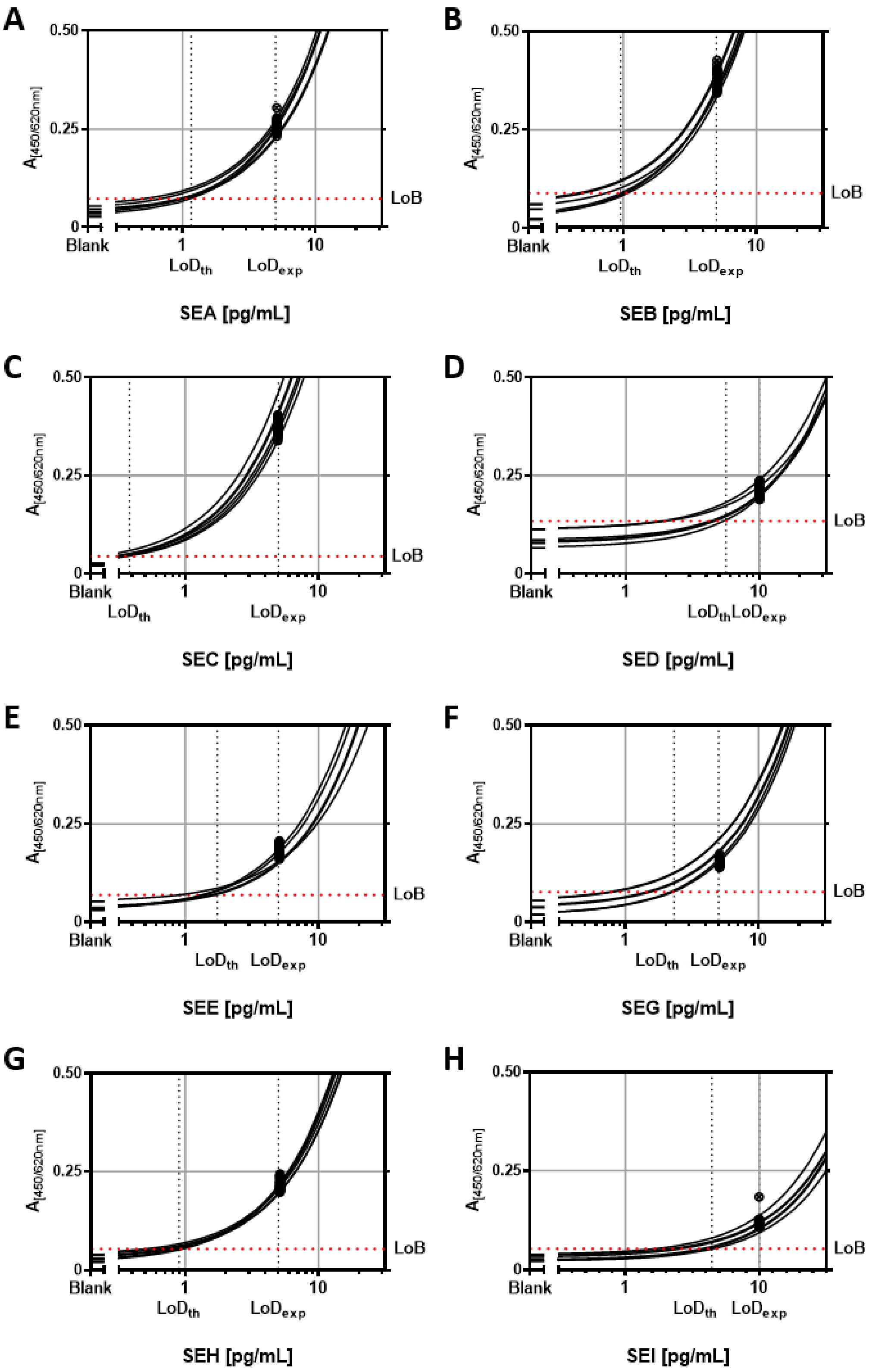


**Figure S5.** Determination of the detection limits for the eight sandwich ELISAs specific for SEA to SEI. The theoretical detection limit (LoD$_{th}$), experimentally confirmed detection limit (LoD$_{exp}$) and limit of blank (LoB) are depicted by zooming into the individual ELISA curves of the eight sandwich ELISAs. The capture mAbs were A) SEA388 + SEA2353, B) S1001, C) SEC371, D) SED1280, E) SEE33, F) SEG5, G) SEH1236, and H) SEI467, which were

immobilised onto 96-well microtiter plates. The respective target SE was incubated in serial dilutions as well as in the according concentration obtained in the $LoD_{exp}$ validation procedure. Detection in the ELISA setup involved using individual biotinylated mAbs: A) SEA165, B) S419, C) SEC290, D) SED333, E) SEE1524, F) SEG158, G) SEH449, and H) SEI92, listed in corresponding order, which was followed by streptavidin conjugated to horseradish peroxidase. The LoB was defined as the 95% confidence interval of the mean of all 56 measured blank values per sandwich ELISA. The "theoretical LoD" $LoD_{th}$ was defined as the mean of 56 blank measurements plus three times their SD of each sandwich ELISA in four experiments. The according $LoD_{exp}$ was determined experimentally and rounded to the nearest multiple of five for evaluation purposes. To determine this, ten samples at the $LoD_{exp}$ level were analysed, each in quadruplicate, shown as black dots. $LoD_{th}$ and $LoD_{exp}$ are indicated as vertical black dotted lines, while LoB is indicated as a red horizontal dotted line.

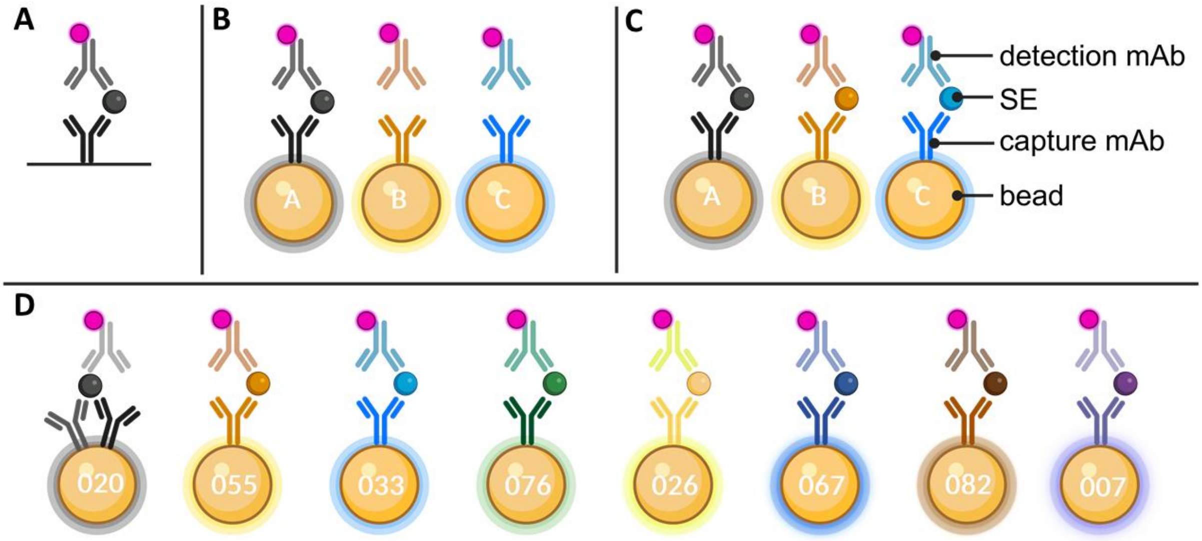


**Figure S6.** Set up of ELISA and multiplex SIA detecting one or multiple SE. A) The sandwich ELISA consists of a capture monoclonal antibody (mAb) coated onto the well of a titre plate and the detection mAb, here depicted as black and grey, respectively, targeting one SE (here SEA in black) per assay. B) The multiplex SIA principally consists of different beads coated with different capture mAbs (black, yellow, and blue beads coated with mAbs targeting individual SEs, e.g. SEA, SEB, and SEC, respectively); after capturing their target SE (here SEA in black), a mixture of biotinylated detection mAbs (here shown in grey, orange, and light blue for mAbs targeting SEA, SEB, and SEC, respectively) is added. This describes the "single-toxin detection mode" using the multiplex SIA. C) As described for B) with the difference that multiple SEs (SEA, SEB, and SEC in black, orange, and blue, respectively) are present in the sample. This principle set up describes the "multi-toxin detection mode" using the multiplex SIA. Note that in B) and C) additional potential cross-reactivities have to be considered and excluded when setting up the multiplex approach which are not relevant for A), namely the cross-reactivities of all antigens with all antibodies present in the mixture and the cross-reactivities among all antibodies in the approach. D) The fully developed multiplex SIA developed in this work consists of eight bead regions (020, 055, 033, 076, 026, 067, 082, and 007 from left to right) which were coated with mAbs SEA388 + SEA2353 (for SEA), S1851 (for SEB), SEC371 (for SEC), SED1280 (for SED), SEE1524 (for SEE), SEG5 (for SEG), SEH449 (for SEH), and SEI242 (for SEI; from left to right), respectively. The mixture of biotinylated detection mAbs consists of SEA165, S419, SEC290, SED9, SEE33, SEG158, SEH1236, and SEI92 (left to right on top, for SEA to SEI), allowing for simultaneous detection of multiple SE targets. The SE antigens SEA (black), SEB (orange), SEC1 (light blue), SED (green), SEE (yellow), SEG (dark blue), SEH (brown), and SEI (purple) can be detected simultaneously in one well. Created in BioRender. Dettmann, P. (2025) https://BioRender.com/331cysv

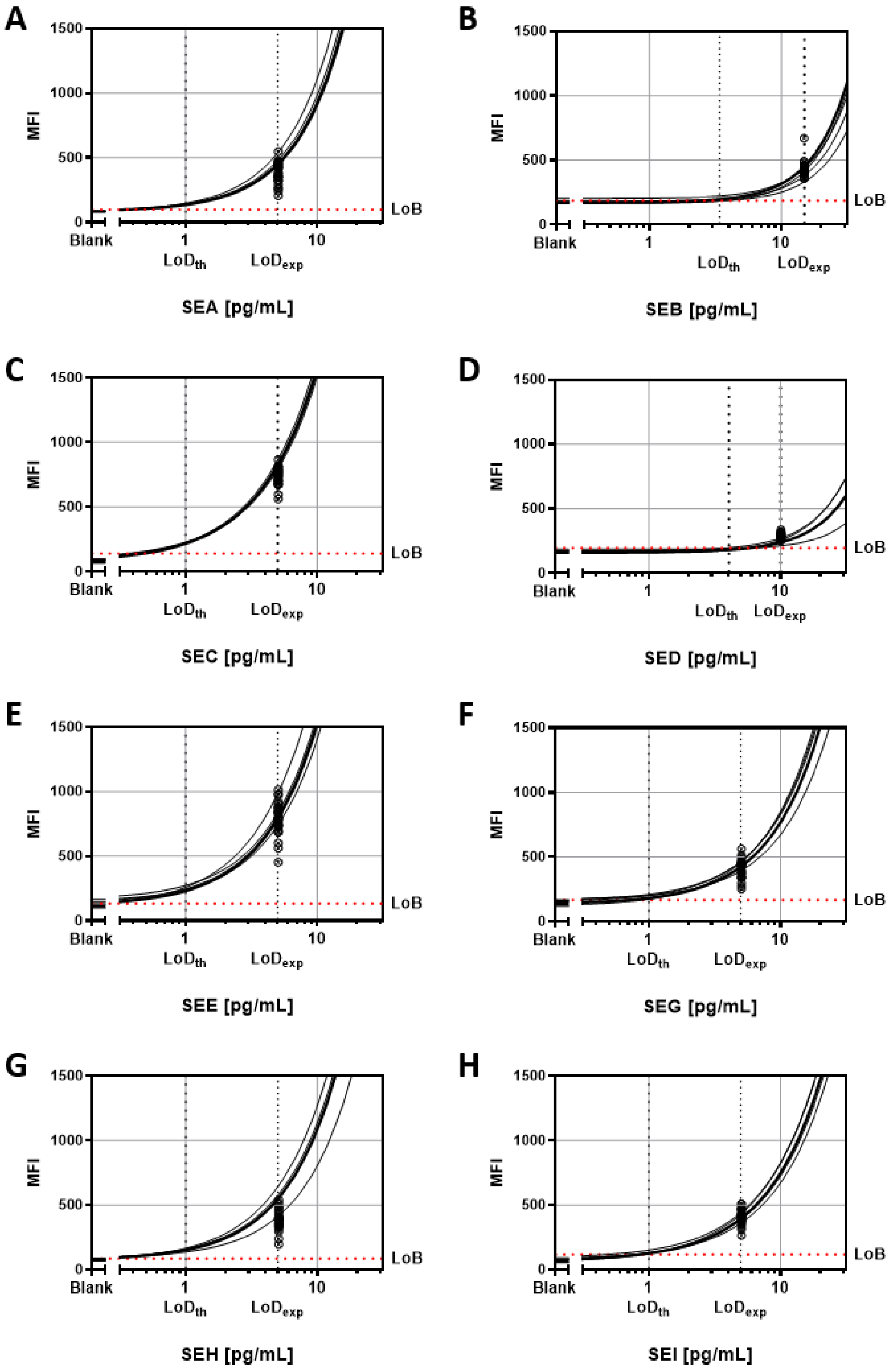


**Figure S7.** Determination of the detection limit of SEA to SEI in the multiplex SIA. The theoretical detection limit ($LoD_{th}$), the experimentally confirmed detection limit ($LoD_{exp}$), and limit of blank (LoB) are depicted by zooming into the individual ELISA curves of the multiplex SIA. The capture mAbs A) SEA388 + SEA2353, B) S1851 for SEB, C) SEC371, D) SED1280, E) SEE1524, F) SEG5, G) SEH449, and H) SEI242 were immobilised onto paramagnetic microspheres. The mixture of SEA to SEI were incubated in serial dilutions as well as in the according concentration

obtained in the $LoD_{exp}$ validation procedure, either in single toxin or toxin mixtures. Detection involved using individual biotinylated mAbs: A) SEA165, B) S419 for SEB, C) SEC290, D) SED9, E) SEE33, F) SEG158, G) SEH1236, and H) SEI92, listed in corresponding order, followed by the application of streptavidin-phycoerythrin. The LoB was defined as the 95% confidence interval of the mean of all 80 measured blank values. The $LoD_{th}$ was defined as the mean of 80 blank measurements plus three times their SD in four experiments. The according $LoD_{exp}$ was rounded to the nearest multiple of five for evaluation purposes. To determine this, ten samples at the $LoD_{exp}$ level were analysed, single toxin $LOD_{exp}$ in duplicate and toxin mixture $LOD_{exp}$ in triplicate shown as black dots. $LoD_{th}$ and $LoD_{exp}$ are indicated as vertical black dotted lines, while LoB is indicated as a red horizontal dotted line.

**Table S1.** Overview of whole genome sequenced bacterial strains and the results of SE analysis via multiplex SIA in their liquid-culture supernatants.

| **Isolates (species/ source)** | | ***sea* / SEA** | | ***seb* / SEB** | | | | ***sec* / SEC** | | | | ***sed* / SED** | ***see* / SEE** | ***seg* / SEG** | | | ***seh* / SEH** | | ***sei* / SEI** | | | **Other *se*** |
|---|---|---|---|---|---|---|---|---|---|---|---|---|---|---|---|---|---|---|---|---|---|---|
| | | **v1** | **v2** | **v1** | **v2** | **v3** | **v4** | **v1** | **v2** | **v3** | **v4** | | | **v1** | **v2** | **v3** | **v1** | **v2** | **v1** | **v2** | **v3** | |
| 08S00573 | *se* | - | - | - | - | - | - | - | - | - | + | + | - | + | - | - | - | - | + | - | - | *selj, sel, sem, sen, seo, ser, selx* |
| (*S. aureus*/ BfR) | SE | - | | - | | | | + | | | | + | - | + | | | - | | + | | | |
| 08S00574 | *se* | - | - | - | - | - | - | - | + | - | - | - | - | - | - | + | - | + | - | - | + | *sel, sem, sen, seo, selu, selx* |
| (*S. aureus*/ BfR) | SE | - | | - | | | | + | | | | - | - | - | | | + | | + | | | |
| 08S00575 | *se* | - | - | - | - | - | - | - | - | - | - | - | + | - | - | - | - | - | - | - | - | *seq* |
| (*S. aureus*/ BfR) | SE | - | | - | | | | - | | | | + | + | + | | | - | | - | | | |
| 08S01548 | *se* | - | - | - | - | - | - | + | - | - | - | - | - | + | - | - | - | - | - | - | - | *sel, sem, sen, seo, selu, selv, tst* |
| (*S. aureus*/ BfR) | SE | - | | - | | | | + | | | | - | - | + | | | - | | - | | | |
| 09-00925 | *se* | - | - | - | - | - | - | - | - | - | - | - | - | + | - | - | - | - | + | - | - | *sem, sen, seo, selx* |
| (*S. aureus*/ RKI) | SE | - | | - | | | | - | | | | - | - | + | | | - | | + | | | |
| 09S00575 | *se* | - | - | - | - | - | - | - | - | - | - | - | + | - | - | - | - | - | - | - | - | *seq* |
| (*S. aureus*/ BfR) | SE | - | | - | | | | - | | | | - | + | - | | | - | | - | | | |
| 11S00617 | *se* | - | - | - | - | - | - | - | - | - | - | - | - | - | - | - | - | - | - | - | - | *sep* |
| (*S. aureus*/ BfR) | SE | - | | - | | | | - | | | | - | - | - | | | - | | - | | | |
| 12-01857 | *se* | - | - | - | - | - | - | - | - | - | - | - | - | + | - | - | - | - | + | - | - | *sem, sen, seo, selx* |
| (*S. aureus*/ RKI) | SE | - | | - | | | | - | | | | - | - | + | | | - | | + | | | |
| 12-03280 | *se* | - | + | - | - | - | - | - | - | - | - | - | - | - | + | - | - | - | - | - | + | *sem, sen, seo, selu, tst* |
| (*S. aureus*/ RKI) | SE | + | | - | | | | - | | | | - | - | + | | | - | | + | | | |
| 12S00034 | *se* | - | - | - | - | - | - | - | - | - | - | - | - | + | - | - | - | - | + | - | - | *sem, sen, seo, selx* |
| (*S. aureus*/ BfR) | SE | - | | - | | | | - | | | | - | - | + | | | - | | + | | | |
| 13-01702 | *se* | - | + | - | - | - | - | + | - | - | - | - | - | - | - | - | - | - | - | - | - | *sek, sel, seq, tst* |
| (*S. aureus*/ RKI) | SE | + | | - | | | | + | | | | - | - | - | | | - | | - | | | |

| Isolates (species/ source) | | *sea* / SEA | | *seb* / SEB | | | | *sec* / SEC | | | | *sed* / SED | *see* / SEE | *seg* / SEG | | | *seh* / SEH | | *sei* / SEI | | | Other *se* |
|---|---|---|---|---|---|---|---|---|---|---|---|---|---|---|---|---|---|---|---|---|---|---|
| | | v1 | v2 | v1 | v2 | v3 | v4 | v1 | v2 | v3 | v4 | | | v1 | v2 | v3 | v1 | v2 | v1 | v2 | v3 | |
| 13-01965 | *se* | - | - | - | - | - | - | - | - | - | - | + | - | + | - | - | - | - | + | - | - | *selj, sem, sen, seo, sep, ser, selx* |
| (*S. aureus*/ RKI) | SE | - | | - | | | | - | | | | + | - | + | | | - | | + | | | |
| 13-03539 | *se* | - | - | - | - | - | - | - | + | - | - | - | - | + | - | - | - | - | + | - | - | *sel, sem, sen, seo, selx* |
| (*S. aureus*/ RKI) | SE | - | | - | | | | + | | | | - | - | + | | | - | | + | | | |
| 13-ST00233 | *se* | - | - | - | - | - | - | - | - | - | - | - | - | - | - | - | + | - | - | - | - | - |
| (*S. aureus*/ BfR) | SE | - | | - | | | | - | | | | - | - | - | | | + | | - | | | |
| 13-ST00641 | *se* | - | + | - | - | - | - | - | - | - | - | - | - | - | + | - | - | + | - | - | + | *sem, sen, seo, selu, selx, tst* |
| (*S. aureus*/ BfR) | SE | + | | - | | | | - | | | | - | - | - | | | + | | - | | | |
| 14-00366 | *se* | - | - | - | - | - | - | - | + | - | - | - | - | + | - | - | - | - | + | - | - | *sel, sem, sen, seo, selx* |
| (*S. aureus*/ RKI) | SE | - | | - | | | | + | | | | - | - | + | | | - | | + | | | |
| 14-00392 | *se* | - | - | - | - | - | - | - | + | - | - | - | - | + | - | - | - | - | + | - | - | *sel, sem, sen, seo, selx* |
| (*S. aureus*/ RKI) | SE | - | | - | | | | + | | | | - | - | + | | | - | | + | | | |
| 14-00471 | *se* | - | - | - | - | - | - | + | - | - | - | - | - | + | - | - | - | - | + | - | - | *sel, sem, sen, seo, selx, tst* |
| (*S. aureus*/ RKI) | SE | - | | - | | | | + | | | | - | - | - | | | - | | + | | | |
| 14-00475 | *se* | - | - | - | - | - | - | - | + | - | - | - | - | + | - | - | - | - | + | - | - | *sel, sem, sen, seo* |
| (*S. aureus*/ RKI) | SE | - | | - | | | | + | | | | - | - | + | | | - | | + | | | |
| 14-00507 | *se* | + | - | - | - | - | - | + | - | - | - | - | - | + | - | - | - | - | + | - | - | *sel, sem, sen, seo, selx, tst* |
| (*S. aureus*/ RKI) | SE | + | | - | | | | + | | | | - | - | + | | | - | | + | | | |
| 14-00511 | *se* | + | - | - | - | - | - | + | | - | - | - | - | + | - | - | - | - | + | - | - | *sel, sem, sen, seo, selx, tst* |
| (*S. aureus*/ RKI) | SE | + | | - | | | | + | | | | - | - | + | | | - | | + | | | |
| 14-00515 | *se* | - | - | - | - | - | - | - | - | - | - | - | - | + | - | - | - | - | + | - | - | *sem, sen, seo, selx* |
| (*S. aureus*/ RKI) | SE | - | | - | | | | - | | | | - | - | - | | | - | | + | | | |

| Isolates | | *sea* / SEA | | *seb* / SEB | | | | *sec* / SEC | | | | *sed* / | *see* / | *seg* / SEG | | | *seh* / SEH | | *sei* / SEI | | | Other *se* |
|---|---|---|---|---|---|---|---|---|---|---|---|---|---|---|---|---|---|---|---|---|---|---|
| (species/ source) | | v1 | v2 | v1 | v2 | v3 | v4 | v1 | v2 | v3 | v4 | SED | SEE | v1 | v2 | v3 | v1 | v2 | v1 | v2 | v3 | |
| 14-00552 | *se* | - | - | - | - | - | - | - | - | - | - | + | - | + | - | - | - | - | + | - | - | *sem, sen, seo, sep, sep, ser, selx* |
| (*S. aureus*/ RKI) | SE | - | | - | | | | - | | | | + | - | + | | | - | | + | | | |
| 14-00594 | *se* | - | - | - | - | - | - | - | - | - | - | - | - | - | - | +** | - | - | - | + | - | *sem, sen, seo, selu, selx, sely* |
| (*S. aureus*/ RKI) | SE | - | | - | | | | - | | | | - | - | + | | | - | | + | | | |
| 14-02531 | *se* | - | - | - | - | - | - | - | - | - | - | - | - | + | - | - | - | - | + | - | - | *sem, sen, seo, selx, tst* |
| (*S. aureus*/ RKI) | SE | - | | - | | | | - | | | | - | - | + | | | - | | + | | | |
| 14-02652 | *se* | - | - | - | - | - | - | - | - | - | - | - | - | + | - | - | - | - | + | - | - | *sem, seo* |
| (*S. aureus*/ RKI) | SE | - | | - | | | | - | | | | + | - | + | | | - | | + | | | |
| 14-ST00756 | *se* | - | - | - | - | - | - | - | - | - | - | - | - | + | - | - | - | - | + | - | - | - |
| (*S. aureus*/ BfR) | SE | - | | - | | | | - | | | | - | - | + | | | - | | + | | | |
| 14-ST00757 | *se* | - | + | - | - | - | - | - | - | + | - | - | - | - | - | - | - | - | - | - | - | *sel, tst* |
| (*S. aureus*/ BfR) | SE | + | | - | | | | + | | | | - | - | - | | | - | | - | | | |
| 15-02754 | *se* | - | - | - | + | - | - | - | - | - | - | - | - | - | + | - | - | - | - | + | - | *sem, sen, seo, selu, selx, sely* |
| (*S. aureus*/ RKI) | SE | - | | + | | | | - | | | | - | - | + | | | - | | + | | | |
| 15-02790 | *se* | - | - | - | - | - | - | - | - | - | - | - | - | + | | - | - | - | + | | | *sem, sen, seo, selx, tst* |
| (*S. aureus*/ RKI) | SE | - | | - | | | | - | | | | - | - | + | | | - | | + | | | |
| 15-ST00110 | *se* | - | - | - | - | - | - | - | - | - | - | - | - | - | - | - | - | - | - | - | + | *sem, sen, seo, selu, selx, sely* |
| (*S. aureus*/ BfR) | SE | - | | - | | | | - | | | | + | - | - | | | - | | + | | | |
| 15-ST00179 | *se* | - | - | - | - | - | - | - | - | - | - | - | - | - | - | - | - | - | - | - | - | - |
| (*S. aureus*/ BfR) | SE | - | | - | | | | - | | | | - | - | - | | | - | | - | | | |
| 15-ST00398 | *se* | - | - | - | - | - | - | - | - | - | - | - | - | - | - | - | - | - | - | - | - | *sep* |
| (*S. aureus*/ BfR) | SE | - | | - | | | | - | | | | - | - | + | | | - | | - | | | |

| Isolates (species/ source) | | *sea* / SEA v1 | v2 | *seb* / SEB v1 | v2 | v3 | v4 | *sec* / SEC v1 | v2 | v3 | v4 | *sed* / SED | *see* / SEE | *seg* / SEG v1 | v2 | v3 | *seh* / SEH v1 | v2 | *sei* / SEI v1 | v2 | v3 | Other *se* |
|---|---|---|---|---|---|---|---|---|---|---|---|---|---|---|---|---|---|---|---|---|---|---|
| 15-ST00399 | *se* | - | - | - | - | - | - | - | - | - | - | - | - | + | - | - | - | - | + | - | - | *sem, sen, seo, selx* |
| (*S. aureus*/ BfR) | SE | - | | - | | | | - | | | | - | - | + | | | - | | + | | | |
| 15-ST00430 | *se* | - | - | - | - | + | - | - | - | - | - | - | - | - | - | - | - | - | - | - | - | - |
| (*S. aureus*/ BfR) | SE | - | | + | | | | - | | | | - | - | - | | | - | | - | | | |
| 15-ST00439 | *se* | - | + | - | - | - | - | - | - | - | - | - | - | - | - | - | - | - | - | - | - | - |
| (*S. aureus*/ BfR) | SE | + | | - | | | | - | | | | - | - | - | | | - | | - | | | |
| 15-ST00440 | *se* | - | - | - | - | - | - | - | - | + | - | - | - | - | - | - | - | - | - | - | - | *sel, tst* |
| (*S. aureus*/ BfR) | SE | - | | - | | | | + | | | | - | - | - | | | - | | - | | | |
| 15-ST00441 | *se* | - | - | - | - | - | - | - | - | + | - | - | - | - | - | - | - | - | - | - | - | *sel, tst* |
| (*S. aureus*/ BfR) | SE | - | | - | | | | + | | | | - | - | - | | | - | | - | | | |
| 15-ST00521 | *se* | - | - | - | - | - | - | - | - | + | - | - | - | - | - | - | - | - | - | - | - | *sel, tst* |
| (*S. aureus*/ BfR) | SE | - | | - | | | | + | | | | - | - | - | | | - | | + | | | |
| 15-ST00539 | *se* | - | - | + | - | - | - | - | - | - | - | - | - | - | - | - | - | - | - | - | - | *sep* |
| (*S. aureus*/ BfR) | SE | - | | + | | | | - | | | | - | - | - | | | - | | - | | | |
| 15-ST00574 | *se* | - | - | - | - | - | - | - | - | - | - | - | - | - | + | - | - | - | - | - | + | *sem, sen, seo, selu, selx* |
| (*S. aureus*/ BfR) | SE | - | | - | | | | - | | | | - | - | - | | | - | | + | | | |
| 15-ST00670 | *se* | - | - | - | - | - | - | - | - | - | - | - | - | + | - | - | - | - | + | - | - | *sem, sen, seo, selx* |
| (*S. aureus*/ BfR) | SE | - | | - | | | | - | | | | + | - | + | | | - | | + | | | |
| 16-00175 | *se* | - | - | - | - | - | - | - | - | - | - | +* | - | + | - | - | - | - | + | - | - | *sem, sen, seo, selx* |
| (*S. aureus*/ RKI) | SE | - | | - | | | | - | | | | - | - | + | | | - | | + | | | |
| 16-00532 | *se* | - | - | - | - | - | - | - | - | - | - | +* | - | + | - | - | - | - | + | - | - | *sem, sen, seo, selx* |
| (*S. aureus*/ RKI) | SE | - | | - | | | | - | | | | - | - | + | | | - | | + | | | |

| Isolates (species/ source) | | *sea* / SEA | | *seb* / SEB | | | | *sec* / SEC | | | | *sed* / SED | *see* / SEE | *seg* / SEG | | | *seh* / SEH | | *sei* / SEI | | | Other *se* |
|---|---|---|---|---|---|---|---|---|---|---|---|---|---|---|---|---|---|---|---|---|---|---|
| | | v1 | v2 | v1 | v2 | v3 | v4 | v1 | v2 | v3 | v4 | | | v1 | v2 | v3 | v1 | v2 | v1 | v2 | v3 | |
| 16-01004 | *se* | - | - | - | - | - | - | - | - | - | - | - | - | - | - | - | + | - | - | - | - | *selx* |
| (*S. aureus*/ RKI) | SE | - | | - | | | | - | | | | - | - | + | | | + | | - | | | |
| 16-01130 | *se* | - | - | - | - | - | - | - | - | - | - | - | - | + | - | - | - | - | + | - | - | *sem, sen, seo, selx* |
| (*S. aureus*/ RKI) | SE | - | | - | | | | - | | | | - | - | + | | | - | | + | | | |
| 16-01169 | *se* | - | - | - | - | - | - | - | + | - | - | - | - | + | - | - | - | - | + | - | - | *sel, sem, sen, seo, selx* |
| (*S. aureus*/ RKI) | SE | - | | - | | | | + | | | | - | - | + | | | - | | + | | | |
| 16-01327 | *se* | - | - | - | - | - | - | - | - | - | - | - | - | - | + | - | - | - | - | + | - | *sem, sen, seo, selu* |
| (*S. aureus*/ RKI) | SE | - | | - | | | | - | | | | - | - | + | | | - | | + | | | |
| 16-01370 | *se* | - | - | - | - | - | - | - | + | - | - | - | - | + | | - | - | - | + | - | - | *sel, sem, sen, selx* |
| (*S. aureus*/ RKI) | SE | - | | - | | | | + | | | | - | - | + | | | - | | + | | | |
| 16-01940 | *se* | - | - | - | - | - | - | - | - | - | - | - | - | - | + | - | - | - | - | - | + | *sem, sen, seo, selu, tst* |
| (*S. aureus*/ RKI) | SE | - | | - | | | | - | | | | - | - | + | | | - | | + | | | |
| 16-02151 | *se* | - | - | - | - | - | - | - | - | - | + | - | - | + | - | - | - | - | + | - | - | *selj, sel, sem, sen, seo* |
| (*S. aureus*/ RKI) | SE | - | | - | | | | + | | | | - | - | + | | | - | | + | | | |
| 16-02200 | *se* | - | - | - | - | - | + | - | - | - | - | - | - | + | - | - | - | - | + | - | - | *sem, sen, seo, selx, sely* |
| (*S. aureus*/ RKI) | SE | - | | + | | | | - | | | | - | - | + | | | - | | + | | | |
| 16-03400 | *se* | - | - | - | - | - | - | - | - | - | - | - | - | - | + | - | - | - | - | - | + | *sem, sen, seo, selu, tst* |
| (*S. aureus*/ RKI) | SE | - | | - | | | | - | | | | + | - | + | | | - | | + | | | |
| 16-ST00051 | *se* | + | - | - | - | - | - | - | - | - | - | - | - | - | - | - | - | - | - | - | - | - |
| (*S. aureus*/ BfR) | SE | + | | - | | | | - | | | | - | - | - | | | - | | - | | | |
| 16-ST00052 | *se* | - | - | - | + | - | - | - | - | - | - | - | - | + | - | - | - | - | + | - | - | *sem, sen, seo, selx* |
| (*S. aureus*/ BfR) | SE | - | | + | | | | - | | | | - | - | + | | | - | | + | | | |

| Isolates (species/ source) | | *sea* / SEA | | *seb* / SEB | | | | *sec* / SEC | | | | *sed* / SED | *see* / SEE | *seg* / SEG | | | *seh* / SEH | | *sei* / SEI | | | Other *se* |
|---|---|---|---|---|---|---|---|---|---|---|---|---|---|---|---|---|---|---|---|---|---|---|
| | | v1 | v2 | v1 | v2 | v3 | v4 | v1 | v2 | v3 | v4 | | | v1 | v2 | v3 | v1 | v2 | v1 | v2 | v3 | |
| 16-ST00082 | *se* | - | - | - | - | - | - | - | - | - | - | - | - | - | - | - | - | - | - | - | - | - |
| (*S. aureus*/ BfR) | SE | - | | - | | | | - | | | | - | - | - | | | - | | - | | | |
| 16-ST00085 | *se* | - | - | - | - | - | - | - | - | - | - | + | - | + | - | - | - | - | + | - | - | *selj, sem, sen, seo, ser, selx* |
| (*S. aureus*/ BfR) | SE | - | | - | | | | - | | | | + | - | + | | | - | | + | | | |
| 16-ST00091 | *se* | - | - | - | - | - | - | - | + | - | - | - | - | + | - | - | - | - | + | - | - | *sel, sem, sen, seo, selx* |
| (*S. aureus*/ BfR) | SE | - | | - | | | | + | | | | - | - | + | | | - | | + | | | |
| 16-ST00139 | *se* | - | - | - | - | - | - | - | - | - | - | - | - | - | - | - | - | - | - | - | - | - |
| (*S. aureus*/ BfR) | SE | - | | - | | | | - | | | | - | - | - | | | - | | - | | | |
| 16-ST00147 | *se* | + | - | - | - | - | - | - | - | - | - | - | - | - | - | - | - | - | - | - | - | - |
| (*S. aureus*/ BfR) | SE | + | | - | | | | - | | | | - | - | - | | | - | | - | | | |
| 16-ST00159 | *se* | - | - | - | + | - | - | - | - | - | - | - | - | - | - | - | - | - | - | - | - | - |
| (*S. aureus*/ BfR) | SE | - | | + | | | | - | | | | - | - | - | | | - | | - | | | |
| 16-ST00336 | *se* | - | - | - | - | - | - | - | - | - | - | - | - | + | - | - | - | - | + | - | - | *sem, sen, seo, selx, sely* |
| (*S. aureus*/ BfR) | SE | - | | - | | | | - | | | | - | - | - | | | - | | + | | | |
| 16-ST00351 | *se* | - | + | - | - | - | - | - | - | + | - | - | - | - | - | - | - | - | - | - | - | *sel, tst* |
| (*S. aureus*/ BfR) | SE | + | | - | | | | + | | | | - | - | - | | | - | | - | | | |
| 16-ST00352 | *se* | - | - | - | - | - | - | - | - | + | - | - | - | - | - | - | - | - | - | - | - | *sel, tst* |
| (*S. aureus*/ BfR) | SE | - | | - | | | | + | | | | - | - | - | | | - | | - | | | |
| 16-ST00416-1 | *se* | - | - | - | - | - | - | - | - | - | - | - | - | - | - | - | - | - | - | - | - | *sep* |
| (*S. aureus*/ BfR) | | - | | - | | | | - | | | | - | - | - | | | - | | - | | | |
| 16-ST00419 | *se* | - | - | + | - | - | - | - | - | - | - | - | - | - | - | - | - | - | - | - | - | *sel, selx* |
| (*S. aureus*/ BfR) | SE | - | | + | | | | - | | | | - | - | - | | | - | | - | | | |

| Isolates (species/ source) | | *sea* / SEA v1 | *sea* / SEA v2 | *seb* / SEB v1 | *seb* / SEB v2 | *seb* / SEB v3 | *seb* / SEB v4 | *sec* / SEC v1 | *sec* / SEC v2 | *sec* / SEC v3 | *sec* / SEC v4 | *sed* / SED | *see* / SEE | *seg* / SEG v1 | *seg* / SEG v2 | *seg* / SEG v3 | *seh* / SEH v1 | *seh* / SEH v2 | *sei* / SEI v1 | *sei* / SEI v2 | *sei* / SEI v3 | Other *se* |
|---|---|---|---|---|---|---|---|---|---|---|---|---|---|---|---|---|---|---|---|---|---|---|
| 16-ST00419-1 | *se* | - | - | - | - | - | - | - | - | - | - | - | - | - | - | - | - | - | - | - | - | *tst* |
| (*S. aureus*/ BfR) | SE | - | | - | | | | - | | | | - | - | - | | | - | | - | | | |
| 16-ST00423 | *se* | - | - | - | + | - | - | - | - | - | + | - | - | + | - | - | - | - | + | - | - | *sel, sem, sen, seo, selx* |
| (*S. aureus*/ BfR) | SE | - | | + | | | | + | | | | + | - | + | | | - | | + | | | |
| 16-ST00436 | *se* | - | - | - | - | - | - | - | - | - | - | - | - | + | - | - | - | - | + | - | - | *sem, sen, seo, selx, sely* |
| (*S. aureus*/ BfR) | SE | - | | - | | | | - | | | | - | - | - | | | - | | + | | | |
| 16-ST00436-2 | *se* | - | - | - | - | - | - | - | - | - | - | - | - | + | - | - | - | - | + | - | - | *sem, sen, seo, selx, sely* |
| (*S. aureus*/ BfR) | SE | - | | - | | | | - | | | | - | - | + | | | - | | + | | | |
| 16-ST00438-2 | *se* | - | - | - | - | - | - | - | - | - | - | + | - | + | - | - | - | - | + | - | - | *selj, sem, sen, seo, sep, ser, selx* |
| (*S. aureus*/ BfR) | SE | - | | - | | | | - | | | | + | - | + | | | - | | + | | | |
| 16-ST00582 | *se* | + | - | - | - | - | - | - | - | - | - | - | - | - | - | - | + | - | - | - | - | - |
| (*S. aureus*/ BfR) | SE | + | | - | | | | - | | | | - | - | - | | | + | | - | | | |
| 16-ST00732 | *se* | - | - | - | - | - | - | - | - | - | + | - | - | - | - | - | - | - | - | - | - | *sel* |
| (*S. aureus*/ BfR) | SE | - | | - | | | | + | | | | - | - | - | | | - | | - | | | |
| 17-01004 | *se* | - | - | - | - | - | - | - | - | - | - | - | - | - | - | - | + | | - | - | - | *selx* |
| (*S. aureus*/ RKI) | SE | - | | - | | | | - | | | | - | - | - | | | + | | - | | | |
| 17-ST00010 | *se* | - | + | - | - | - | - | - | - | - | - | - | - | - | - | - | - | - | - | - | - | - |
| (*S. aureus*/ BfR) | SE | + | | - | | | | - | | | | + | - | + | | | - | | + | | | |
| 17-ST00011 | *se* | - | - | - | - | - | - | + | - | - | - | - | - | + | - | - | - | - | + | - | - | *sel, sem, sen, seo, sep, selx, tst* |
| (*S. aureus*/ BfR) | SE | - | | - | | | | + | | | | - | - | + | | | - | | + | | | |
| 17-ST00026 | *se* | - | - | - | - | - | - | - | - | - | - | + | - | - | - | - | - | - | - | - | - | *selj, ser* |
| (*S. aureus*/ BfR) | SE | - | | - | | | | - | | | | + | - | - | | | - | | - | | | |

| **Isolates** | | ***sea* / SEA** | | ***seb* / SEB** | | | | ***sec* / SEC** | | | | ***sed* /** | ***see* /** | ***seg* / SEG** | | | ***seh* / SEH** | | ***sei* / SEI** | | | **Other *se*** |
|---|---|---|---|---|---|---|---|---|---|---|---|---|---|---|---|---|---|---|---|---|---|---|
| **(species/ source)** | | **v1** | **v2** | **v1** | **v2** | **v3** | **v4** | **v1** | **v2** | **v3** | **v4** | **SED** | **SEE** | **v1** | **v2** | **v3** | **v1** | **v2** | **v1** | **v2** | **v3** | |
| 17-ST00128 | *se* | + | - | - | - | - | - | - | - | - | - | + | - | - | - | - | - | - | - | - | - | *selj, sek, seq, ser* |
| (*S. aureus*/ BfR) | SE | + | | - | | | | - | | | | + | - | - | | | - | | - | | | |
| 17-ST00292 | *se* | - | - | - | - | - | - | + | - | - | - | - | - | + | - | - | - | - | + | - | - | *sel, sem, sen, seo, selx, tst* |
| (*S. aureus*/ BfR) | SE | - | | - | | | | + | | | | - | - | + | | | - | | + | | | |
| 17-ST00351 | *se* | + | - | - | - | - | - | - | - | - | - | + | - | - | - | - | - | - | - | - | - | *selj, ser* |
| (*S. aureus*/ BfR) | SE | + | | - | | | | - | | | | + | - | - | | | - | | - | | | |
| 17-ST00452 | *se* | - | - | - | - | - | - | - | - | - | - | - | - | - | - | - | - | - | - | - | - | - |
| (*S. aureus*/ BfR) | SE | - | | - | | | | - | | | | - | - | - | | | - | | - | | | |
| 18-01509 | *se* | - | - | + | - | - | - | - | - | - | - | - | - | - | - | - | - | - | - | - | - | *sek, seq, selx, sely* |
| (*S. aureus*/ RKI) | SE | - | | + | | | | - | | | | - | - | - | | | - | | - | | | |
| 18-01582 | *se* | - | - | - | - | - | - | - | - | - | - | - | - | + | - | - | - | - | + | - | - | *selj, sem, sen, seo, selx, tst* |
| (*S. aureus*/ RKI) | SE | - | | - | | | | - | | | | - | - | - | | | - | | + | | | |
| 18-02513 | *se* | - | - | - | - | - | - | - | - | - | - | - | - | + | - | - | - | - | + | - | - | *sem, sen, seo, selx, tst* |
| (*S. aureus*/ RKI) | SE | - | | - | | | | - | | | | - | - | + | | | - | | + | | | |
| 18-02689 | *se* | - | - | - | - | - | - | - | + | - | - | - | - | - | - | + | - | - | - | + | - | *sel, sem, sen, seo, selu, selx, tst* |
| (*S. aureus*/ RKI) | SE | - | | - | | | | + | | | | - | - | + | | | - | | + | | | |
| 18-02690 | *se* | - | + | - | - | - | - | + | - | - | - | - | + | - | - | - | - | - | - | - | - | *sek, sel, seq* |
| (*S. aureus*/ RKI) | SE | + | | - | | | | + | | | | - | + | - | | | - | | - | | | |
| 18-ST00090 | *se* | - | - | - | - | - | - | - | - | - | - | - | - | - | - | +** | - | + | - | - | + | *sem, sen, seo, selu, selx, sely* |
| (*S. aureus*/ BfR) | SE | - | | - | | | | - | | | | - | - | - | | | + | | + | | | |
| 18-ST00093 | *se* | - | + | - | - | - | - | - | - | - | - | - | - | - | + | - | - | - | - | - | + | *sem, sen, seo, selu, selx, tst* |
| (*S. aureus*/ BfR) | SE | + | | - | | | | - | | | | - | - | + | | | - | | + | | | |

| Isolates (species/ source) | | *sea* / SEA | | *seb* / SEB | | | | *sec* / SEC | | | | *sed* / | *see* / | *seg* / SEG | | | *seh* / SEH | | *sei* / SEI | | | Other *se* |
|---|---|---|---|---|---|---|---|---|---|---|---|---|---|---|---|---|---|---|---|---|---|---|
| | | v1 | v2 | v1 | v2 | v3 | v4 | v1 | v2 | v3 | v4 | SED | SEE | v1 | v2 | v3 | v1 | v2 | v1 | v2 | v3 | |
| 18-ST00095 | *se* | + | - | - | - | - | - | - | - | - | - | +** | - | - | - | - | - | - | - | - | - | *selj, ser* |
| (*S. aureus*/ BfR) | SE | + | | - | | | | - | | | | - | - | - | | | - | | - | | | |
| 18-ST00096 | *se* | + | - | - | - | - | - | - | - | - | - | | - | - | - | - | - | - | - | - | - | *sek, seq* |
| (*S. aureus*/ BfR) | SE | + | | - | | | | - | | | | - | - | - | | | - | | - | | | |
| 18-ST00169 | *se* | + | - | - | - | + | - | - | - | - | - | - | - | - | - | - | - | - | - | - | - | *selx* |
| (*S. aureus*/ BfR) | SE | + | | + | | | | - | | | | - | - | - | | | - | | - | | | |
| 18-ST00293 | *se* | - | - | - | - | - | - | - | - | - | - | - | - | - | - | - | + | - | - | - | - | - |
| (*S. aureus*/ BfR) | SE | - | | - | | | | - | | | | - | - | - | | | + | | - | | | |
| 18-ST00474 | *se* | - | - | - | - | - | - | - | - | - | - | - | - | - | + | - | - | - | - | + | - | *sem, sen, seo, selu, selx* |
| (*S. aureus*/ BfR) | SE | - | | - | | | | - | | | | - | - | + | | | - | | + | | | |
| 18-ST00526 | *se* | - | - | - | - | - | - | - | - | - | - | - | - | - | - | - | - | - | - | - | - | - |
| (*S. aureus*/ BfR) | SE | - | | - | | | | - | | | | - | - | - | | | - | | - | | | |
| 18-ST00599-2 | *se* | + | - | - | - | - | - | - | - | - | - | - | - | - | - | - | + | - | - | - | - | *sek, seq* |
| (*S. aureus*/ BfR) | SE | + | | - | | | | - | | | | + | - | + | | | + | | - | | | |
| 19-00144 | *se* | + | - | - | - | + | - | - | - | - | - | - | - | - | - | - | + | - | - | - | - | *sek, seq, selx* |
| (*S. aureus*/ RKI) | SE | + | | + | | | | - | | | | + | - | + | | | + | | - | | | |
| 19-00456 | *se* | - | - | - | - | - | - | - | - | - | - | - | - | + | - | - | - | - | + | - | - | *sem, sen, seo, selx, tst* |
| (*S. aureus*/ RKI) | SE | - | | - | | | | - | | | | - | - | - | | | - | | + | | | |
| 19-01991 | *se* | - | - | - | - | - | - | - | - | - | - | - | - | - | + | - | - | - | - | - | + | *sem, sen, seo, selu* |
| (*S. aureus*/ RKI) | SE | - | | - | | | | - | | | | - | - | + | | | - | | + | | | |
| 19-02864 | *se* | - | - | - | - | - | - | - | - | - | - | - | - | - | + | - | - | - | - | - | + | *sem, sen, seo* |
| (*S. aureus*/ RKI) | SE | - | | - | | | | - | | | | - | - | + | | | - | | + | | | |

| Isolates (species/ source) | | *sea* / SEA v1 | v2 | *seb* / SEB v1 | v2 | v3 | v4 | *sec* / SEC v1 | v2 | v3 | v4 | *sed* / SED | *see* / SEE | *seg* / SEG v1 | v2 | v3 | *seh* / SEH v1 | v2 | *sei* / SEI v1 | v2 | v3 | Other *se* |
|---|---|---|---|---|---|---|---|---|---|---|---|---|---|---|---|---|---|---|---|---|---|---|
| 19-02865 | *se* | - | - | - | - | - | - | - | - | - | - | - | - | - | + | - | - | - | - | - | + | *sem, sen* |
| (*S. aureus*/ RKI) | SE | - | | - | | | | - | | | | - | - | + | | | - | | + | | | |
| 19-02901 | *se* | - | - | - | - | - | - | - | - | - | - | - | - | - | + | - | - | - | - | - | + | *sem, sen, seo* |
| (*S. aureus*/ RKI) | SE | - | | - | | | | - | | | | - | - | + | | | - | | + | | | |
| 19-02911 | *se* | - | - | - | - | - | - | - | - | - | - | - | - | - | + | - | - | - | - | - | + | *sem, seo, selu* |
| (*S. aureus*/ RKI) | SE | - | | - | | | | - | | | | - | - | + | | | - | | + | | | |
| 19-03223 | *se* | - | - | + | - | - | - | - | - | - | - | - | - | - | - | - | - | - | - | - | - | *sep, selx* |
| (*S. aureus*/ RKI) | SE | - | | + | | | | - | | | | - | - | - | | | - | | - | | | |
| 19-03502 | *se* | - | - | - | - | - | - | - | - | - | - | - | - | - | + | - | - | - | - | - | + | *sem, sen, seo, selu* |
| (*S. aureus*/ RKI) | SE | - | | - | | | | - | | | | + | - | + | | | - | | + | | | |
| 19-ST00108 | *se* | - | - | - | - | - | - | - | - | - | - | - | - | - | - | +** | - | + | - | - | + | *sem, sen, seo, selu, selx, sely* |
| (*S. aureus*/ BfR) | SE | - | | - | | | | - | | | | - | - | - | | | + | | + | | | |
| 19-ST00670 | *se* | - | - | - | - | - | - | - | - | - | - | - | - | - | - | - | - | - | - | - | - | - |
| (*S. aureus*/ BfR) | SE | - | | - | | | | - | | | | - | - | - | | | - | | - | | | |
| 19-ST00949 | *se* | - | - | - | - | - | - | - | - | - | - | - | - | - | - | - | - | - | - | - | - | - |
| (*S. aureus*/ BfR) | SE | - | | - | | | | - | | | | - | - | - | | | - | | - | | | |
| 19-ST00963 | *se* | - | + | - | - | - | - | - | - | - | - | - | - | + | - | - | - | - | + | - | - | *sem, sen, seo, selx* |
| (*S. aureus*/ BfR) | SE | + | | - | | | | - | | | | - | - | + | | | - | | + | | | |
| 20-00159 | *se* | - | - | - | - | - | - | - | - | - | - | - | - | + | - | - | - | - | + | - | - | *sem, sen, seo, selx, sely* |
| (*S. aureus*/ RKI) | SE | - | | - | | | | - | | | | - | - | + | | | - | | + | | | |
| 20-00891 | *se* | - | - | - | - | - | - | - | - | - | - | - | - | + | - | - | - | - | + | - | - | *sem, sen, seo, sep, selx* |
| (*S. aureus*/ RKI) | SE | - | | - | | | | - | | | | - | - | + | | | - | | + | | | |

| Isolates (species/ source) | | *sea* / SEA | | *seb* / SEB | | | | *sec* / SEC | | | | *sed* / SED | *see* / SEE | *seg* / SEG | | | *seh* / SEH | | *sei* / SEI | | | Other *se* |
|---|---|---|---|---|---|---|---|---|---|---|---|---|---|---|---|---|---|---|---|---|---|---|
| | | v1 | v2 | v1 | v2 | v3 | v4 | v1 | v2 | v3 | v4 | | | v1 | v2 | v3 | v1 | v2 | v1 | v2 | v3 | |
| 20-00967 | *se* | - | - | - | - | + | - | - | - | - | - | - | - | - | - | - | - | - | - | + | - | *sek, sem, sen, seo, seq, selu, selx* |
| (*S. aureus*/ RKI) | SE | - | | + | | | | - | | | | - | - | - | | | - | | + | | | |
| 20-01064 | *se* | - | - | - | - | - | - | - | + | - | - | - | - | + | - | - | - | - | + | - | - | *sel, sem, sen, seo, selx* |
| (*S. aureus*/ RKI) | SE | - | | - | | | | - | | | | - | - | + | | | - | | + | | | |
| 20-01310 | *se* | - | - | - | - | - | - | - | - | - | - | - | - | + | - | - | - | - | + | - | - | *sem, sen, seo, selx, sely* |
| (*S. aureus*/ RKI) | SE | - | | - | | | | - | | | | - | - | + | | | - | | + | | | |
| 20-01793 | *se* | - | - | - | - | - | - | - | - | - | - | - | - | + | - | - | - | - | + | - | - | *sem, sen, seo, selx* |
| (*S. aureus*/ RKI) | SE | - | | - | | | | - | | | | - | - | + | | | - | | + | | | |
| 20-02215 | *se* | - | - | - | - | - | - | - | + | - | - | - | - | + | - | - | - | - | + | - | - | *sel, sem, sen, seo, selx* |
| (*S. aureus*/ RKI) | SE | - | | - | | | | + | | | | - | - | + | | | - | | + | | | |
| 20-02276 | *se* | - | - | - | - | - | - | - | + | - | - | - | - | + | - | - | - | - | - | - | - | *sel, sem, sen, seo, selx* |
| (*S. aureus*/ RKI) | SE | - | | - | | | | + | | | | - | - | + | | | - | | - | | | |
| 20-ST00005 | *se* | - | - | - | - | - | - | - | - | - | - | - | - | - | - | - | - | - | - | - | - | - |
| (*S. aureus*/ BfR) | SE | - | | - | | | | - | | | | - | - | - | | | - | | - | | | |
| 20-ST00335 | *se* | - | - | - | - | - | - | - | - | - | - | - | - | + | - | - | - | - | + | - | - | *selj, sem, sen, seo, sep, ser, ses, set, selx* |
| (*S. aureus*/ BfR) | SE | - | | - | | | | - | | | | - | - | + | | | - | | + | | | |
| 20-ST00363 | *se* | - | - | - | - | - | - | - | - | - | - | - | - | - | - | - | - | - | - | - | - | - |
| (*S. aureus*/ BfR) | SE | - | | - | | | | - | | | | - | - | - | | | - | | - | | | |
| 20-ST00418 | *se* | + | - | - | - | - | - | - | - | - | - | - | - | - | - | - | + | - | - | - | - | *sek, seq* |
| (*S. aureus*/ BfR) | SE | + | | - | | | | - | | | | - | - | - | | | + | | - | | | |
| 20-ST00441 | *se* | - | - | - | - | - | - | - | - | - | - | - | - | + | - | - | - | - | + | - | - | *sem, sen, seo, selx* |
| (*S. aureus*/ BfR) | SE | - | | - | | | | - | | | | - | - | + | | | - | | + | | | |

| Isolates (species/ source) | | *sea* / SEA | | *seb* / SEB | | | | *sec* / SEC | | | | *sed* / | *see* / | *seg* / SEG | | | *seh* / SEH | | *sei* / SEI | | | Other *se* |
|---|---|---|---|---|---|---|---|---|---|---|---|---|---|---|---|---|---|---|---|---|---|---|
| | | v1 | v2 | v1 | v2 | v3 | v4 | v1 | v2 | v3 | v4 | SED | SEE | v1 | v2 | v3 | v1 | v2 | v1 | v2 | v3 | |
| 20-ST00482 | *se* | - | - | - | - | - | - | - | - | - | - | - | - | - | - | - | - | + | - | - | - | - |
| (*S. aureus*/ BfR) | SE | - | | - | | | | - | | | | - | - | + | | | + | | - | | | |
| 20-ST00486 | *se* | - | - | - | - | - | - | - | - | - | - | - | - | - | - | - | - | - | - | - | - | - |
| (*S. aureus*/ BfR) | SE | - | | - | | | | - | | | | - | - | - | | | - | | - | | | |
| 21-00412 | *se* | - | - | - | - | - | - | - | - | - | - | - | - | + | - | - | - | - | + | - | - | *sem, sen, seo, selx* |
| (*S. aureus*/ RKI) | SE | - | | - | | | | - | | | | + | - | + | | | - | | - | | | |
| 21-00492 | *se* | - | - | - | - | - | - | - | - | - | + | - | - | + | - | - | - | - | + | - | - | *sel, sem, sen, seo, selx, sely* |
| (*S. aureus*/ RKI) | SE | - | | - | | | | + | | | | - | - | + | | | - | | + | | | |
| 21-01535 | *se* | - | - | - | - | - | - | - | - | - | - | +** | - | + | - | - | - | - | + | - | - | *selj, sem, sen, seo, sep, ser, selx* |
| (*S. aureus*/ RKI) | SE | - | | - | | | | - | | | | + | - | + | | | - | | + | | | |
| 21-01536 | *se* | - | - | - | - | - | - | - | - | - | - | - | - | + | - | - | - | - | + | - | - | *sem, sen, seo, selx* |
| (*S. aureus*/ RKI) | SE | - | | - | | | | - | | | | - | - | + | | | - | | + | | | |
| 21-01671 | *se* | - | - | - | - | - | - | - | - | - | - | - | - | - | - | - | + | - | - | - | - | *selx* |
| (*S. aureus*/ RKI) | SE | - | | - | | | | - | | | | - | - | + | | | + | | - | | | |
| 21-01745 | *se* | - | - | - | - | - | - | - | - | - | - | + | - | + | - | - | - | - | + | - | - | *selj, sem, sen, seo, sep, ser, selx* |
| (*S. aureus*/ RKI) | SE | - | | - | | | | - | | | | + | - | + | | | - | | + | | | |
| 21-02529 | *se* | - | - | - | - | - | - | + | - | - | - | - | - | + | - | - | - | - | + | - | - | *sel, sem, sen, seo, selx, tst* |
| (*S. aureus*/ RKI) | SE | - | | - | | | | + | | | | - | - | + | | | - | | + | | | |
| 21-02538-2 | *se* | - | - | - | - | - | - | - | - | - | - | - | - | + | - | - | - | - | + | - | - | *sem, sen, seo, selx* |
| (*S. aureus*/ RKI) | SE | - | | - | | | | - | | | | + | - | + | | | - | | + | | | |
| 21-ST00003 | *se* | - | - | - | - | - | - | - | - | - | - | - | - | - | - | - | - | - | - | - | - | - |
| (*S. aureus*/ BfR) | SE | - | | - | | | | - | | | | - | - | - | | | - | | - | | | |

| Isolates | | *sea* / SEA | | *seb* / SEB | | | | *sec* / SEC | | | | *sed* / | *see* / | *seg* / SEG | | | *seh* / SEH | | *sei* / SEI | | | Other *se* |
|---|---|---|---|---|---|---|---|---|---|---|---|---|---|---|---|---|---|---|---|---|---|---|
| (species/ source) | | v1 | v2 | v1 | v2 | v3 | v4 | v1 | v2 | v3 | v4 | SED | SEE | v1 | v2 | v3 | v1 | v2 | v1 | v2 | v3 | |
| 21-ST00243-2 | *se* | + | - | - | - | - | - | - | - | - | - | + | - | - | - | - | - | - | - | - | - | *selj, ser* |
| (*S. aureus*/ BfR) | SE | + | | - | | | | - | | | | + | - | - | | | - | | - | | | |
| 21-ST00357 | *se* | - | - | - | - | - | - | - | - | - | - | - | - | - | - | - | - | - | - | - | - | - |
| (*S. aureus*/ BfR) | SE | - | | - | | | | - | | | | - | - | - | | | - | | - | | | |
| 21-ST00358 | *se* | - | - | - | - | - | - | - | - | - | - | - | - | - | - | - | - | - | - | - | - | - |
| (*S. aureus*/ BfR) | SE | - | | - | | | | - | | | | - | - | - | | | - | | - | | | |
| 22-00027 | *se* | - | - | - | - | - | - | - | - | - | - | - | - | + | - | - | - | - | + | - | - | *sem, sen, seo, selx* |
| (*S. aureus*/ RKI) | SE | - | | - | | | | - | | | | - | - | + | | | - | | + | | | |
| 18-ST00708 | *se* | - | - | - | - | - | - | - | - | - | - | - | - | - | - | - | - | - | - | - | - | - |
| (*S. epidermidis*/ BfR) | SE | - | | - | | | | - | | | | - | - | - | | | - | | - | | | |
| 19-ST00512 | *se* | - | - | - | - | - | - | - | - | - | - | - | - | - | - | - | - | - | - | - | - | - |
| (*S. haemolyticus*/ BfR) | SE | - | | - | | | | - | | | | - | - | - | | | - | | - | | | |
| 19-ST00518 | *se* | - | - | - | - | - | - | - | - | - | - | - | - | - | - | - | - | - | - | - | - | - |
| (*S. epidermidis*/ BfR) | SE | - | | - | | | | - | | | | - | - | - | | | - | | - | | | |
| 19-ST00686 | *se* | - | - | - | - | - | - | - | - | - | - | - | - | - | - | - | - | - | - | - | - | - |
| (*S. epidermidis*/ BfR) | SE | - | | - | | | | - | | | | - | - | - | | | - | | - | | | |
| 20-ST00131 | *se* | - | - | - | - | - | - | - | - | - | - | - | - | - | - | - | - | - | - | - | - | - |
| (*Enterococcus faecalis*/ BfR) | SE | - | | - | | | | - | | | | - | - | - | | | - | | - | | | |
| 20-ST00485 | *se* | - | - | - | - | - | - | - | - | - | - | - | - | - | - | - | - | - | - | - | - | - |
| (*S. epidermidis*/ BfR) | SE | - | | - | | | | - | | | | - | - | - | | | - | | - | | | |

| Isolates (species/ source) | | *sea* / SEA v1 | v2 | *seb* / SEB v1 | v2 | v3 | v4 | *sec* / SEC v1 | v2 | v3 | v4 | *sed* / SED | *see* / SEE | *seg* / SEG v1 | v2 | v3 | *seh* / SEH v1 | v2 | *sei* / SEI v1 | v2 | v3 | Other *se* |
|---|---|---|---|---|---|---|---|---|---|---|---|---|---|---|---|---|---|---|---|---|---|---|
| 21-ST00072 (*S. epidermidis*/ BfR) | *se* | - | - | - | - | - | - | - | - | - | - | - | - | - | - | - | - | - | - | - | - | - |
| | SE | - | | - | | | | - | | | | - | - | - | | | - | | - | | | |
| 21-ST00078 (*S. epidermidis*/ BfR) | *se* | - | - | - | - | - | - | - | - | - | - | - | - | - | - | - | - | - | - | - | - | - |
| | SE | - | | - | | | | - | | | | - | - | - | | | - | | - | | | |
| 21-ST00300 (*S. epidermidis*/ BfR) | *se* | - | - | - | - | - | - | - | - | - | - | - | - | - | - | - | - | - | - | - | - | - |
| | SE | - | | - | | | | - | | | | - | - | - | | | - | | - | | | |
| **Summarised results for SE detection** | | | | | | | | | | | | | | | | | | | | | | |
| True positive (*n*) | | 24 | | 13 | | | | 31 | | | | 12 | 3 | 68 | | | 14 | | 78 | | | n.a. |
| False positive (*n*) | | 0 | | 0 | | | | 0 | | | | 12 | 0 | 8 | | | 0 | | 2 | | | n.a. |
| True negative (*n*) | | 121 | | 132 | | | | 113 | | | | 119 | 142 | 58 | | | 131 | | 63 | | | n.a. |
| False negative (*n*) | | 0 | | 0 | | | | 1 | | | | 2 | 0 | 11 | | | 0 | | 2 | | | n.a. |
| **Median performance parameters** | | | | | | | | | | | | | | | | | | | | | | |
| Specificity (%) | | 100 | | 100 | | | | 100 | | | | 90.8 | 100 | 87.9 | | | 100 | | 98.5 | | | n.a. |
| Sensitivity (%) | | 100 | | 100 | | | | 96.9 | | | | 85.7 | 100 | 86.1 | | | 100 | | 97.5 | | | n.a. |
| Accuracy (%) | | 100 | | 100 | | | | 99.3 | | | | 90.3 | 100 | 86.9 | | | 100 | | 97.9 | | | n.a. |
| **Threshold** | | | | | | | | | | | | | | | | | | | | | | |
| Threshold (MFI) | | 6,030 | | 16,979 | | | | 8,854 | | | | 2,121 | 16,886 | 1,290 | | | 15,680 | | 794 | | | n.a. |
| Threshold (pg/mL) ± SD | | 54.9 ± 3 | | 268.3 ± 20 | | | | 66.7 ± 2 | | | | 87.5 ± 17 | 147.1 ± 12 | 19.7 ± 2 | | | 177.6 ± 20 | | 12.4 ± 1 | | | n.a. |

Reference sequences of the *se* gene variants of the VirulenceFinder database considered in this study can be accessed with the following accession IDs (the numbering of the gene variants can differ from the numbering in the database): *sea.v1* (AP009324.1), *sea.v2* (CP010526.1), *seb.v1* (CP007539.1), *seb.v2* (AB716349.1), *seb.v3* (AB716351.1), *seb.v4* (AB716352.1), *sec.v1* (AB084256.1), *sec.v2* (KF386012.1), *sec.v3* (KF729631.1), *sec.v4* (M28364.1), *sed* (M28521.1), *see* (M21319.1), *seg.v1* (CP001844.2), *seg.v2* (CP002388.1), *seg.v3* (AJ938182.1), *seh.v1* (BX571857.1), *seh.v2* (AY345144.1), *sei.v1* (BA000018.3), *sei.v2* (AJ938182.1), and *sei.v3* (CP002388.1). In regard to the subtypes of SEC, SEC1 is encoded by *sec.v2* and *sec.v3*, SEC2 by *sec.v4*, and SEC3 by *sec.v1*. SE = staphylococcal enterotoxin (protein detection), + = *se* / SE present, - = *se* genes / SE not present, grey = *se* present, green = true positive, yellow = false positive, blue = true negative, orange = false negative, * no promotors, ** truncated gene, SD = standard deviation, n.a. = not applicable. Threshold was calculated by receiver operating characteristic curve analysis and the results were extrapolated to the toxin dilution curve, see Figure S9.

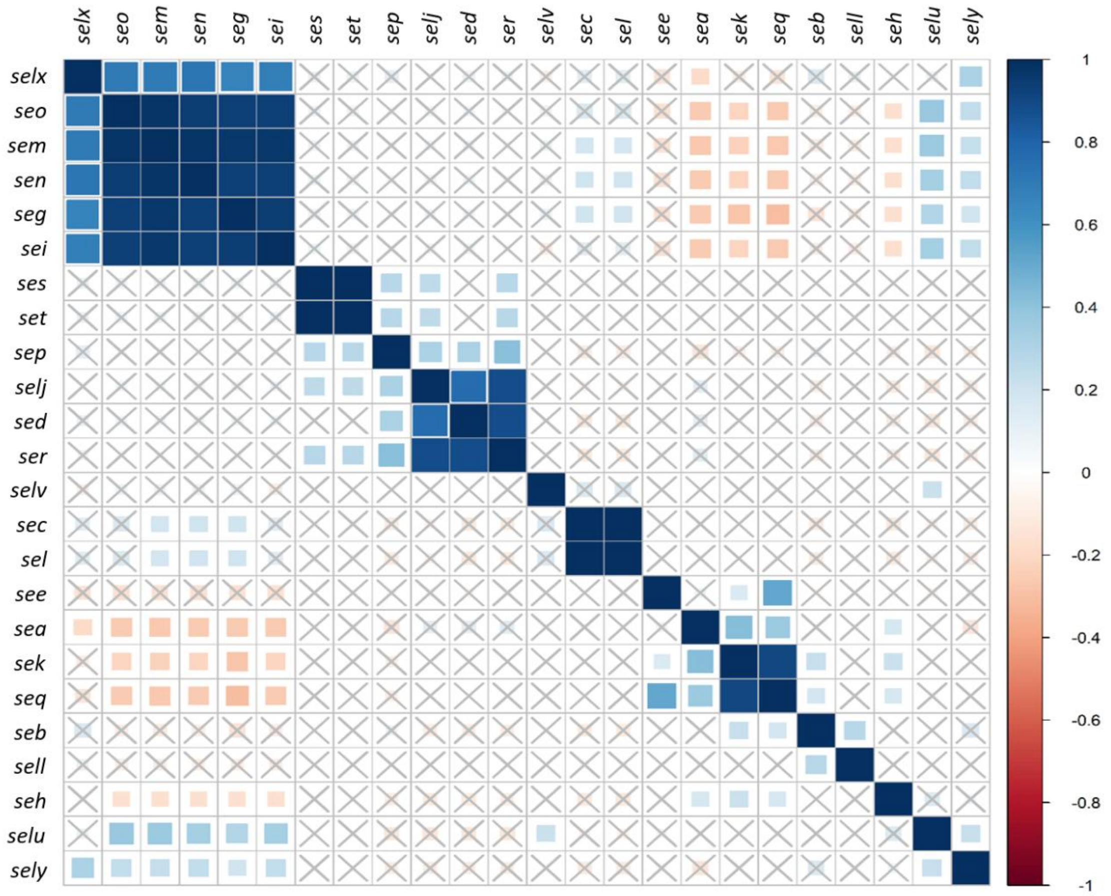


**Figure S8.** Correlation matrix for simultaneous harbouring of enterotoxin genes. All 145 whole genome sequenced bacterial strains were analysed for the correlation of simultaneous encoding for different enterotoxins on the same strain by applying matrix correlation analysis (rstatix package, version 0.7.2) with R (version 4.1.3). As illustrated in the colour gradient scale on the right, the correlation coefficient value between two genes is represented by a colour ranging from dark blue (strong positive correlation, correlation coefficient value = 1, $p$-value < 0.05) to red (strong negative correlation, correlation coefficient value = -1, $p$-value < 0.05). Crosses indicate insignificant correlation coefficients ($p$-value > 0.05). The $p$-value is additionally visualised by the size of the coloured squares.

**Table S2.** Protein alignment of all SEA-SEI incl. variants in presented strains.

| *n* | 14 | 10 | 5 | 4 | 3 | 1 | 9 | 12 | 6 | 5 | 14 | 3 | 57 | 16 | 5 | 10 | 4 | 58 | 6 | 15 |
|---|---|---|---|---|---|---|---|---|---|---|---|---|---|---|---|---|---|---|---|---|
| | **SEA.v1** | **SEA.v2** | **SEB.v1** | **SEB.v2** | **SEB.v3** | **SEB.v4** | **SEC.v1** | **SEC.v2** | **SEC.v3** | **SEC.v4** | **SED** | **SEE** | **SEG.v1** | **SEG.v2** | **SEG.v3** | **SEH.v1** | **SEH.v2** | **SEI.v1** | **SEI.v2** | **SEI.v3** |
| **SEA.v1** | 100 | 98.1 | 31.9 | 31.9 | 31.5 | 31.5 | 29.4 | 27.9 | 27.9 | 29 | 50.6 | 83.3 | 24.7 | 25.1 | 25.1 | 34.4 | 34.4 | 28.7 | 28.3 | 29.4 |
| **SEA.v2** | | 100 | 31.9 | 31.9 | 31.1 | 31.1 | 29.4 | 27.9 | 27.9 | 29 | 49.4 | 82.9 | 24.7 | 25.1 | 25.1 | 34.7 | 34.7 | 28.7 | 28.3 | 29.4 |
| **SEB.v1** | | | 100 | 98.5 | 97 | 96.3 | 68.9 | 67.8 | 67.4 | 66.7 | 34.1 | 31.5 | 43 | 43.4 | 42.6 | 28.4 | 28 | 22.6 | 21.5 | 23.3 |
| **SEB.v2** | | | | 100 | 97.8 | 97 | 67.8 | 67.4 | 67.4 | 65.6 | 34.4 | 31.5 | 43 | 43.4 | 42.6 | 28 | 27.7 | 22.2 | 21.2 | 22.9 |
| **SEB.v3** | | | | | 100 | 99.3 | 67.8 | 67.4 | 67 | 65.6 | 33.7 | 30.8 | 42.6 | 43 | 42.3 | 28.4 | 28 | 21.9 | 21.2 | 22.6 |
| **SEB.v4** | | | | | | 100 | 67 | 66.7 | 66.3 | 64.8 | 33.7 | 30.8 | 41.9 | 42.3 | 41.5 | 28 | 27.7 | 22.2 | 21.5 | 22.9 |
| **SEC.v1** | | | | | | | 100 | 94.4 | 93.3 | 96.3 | 31.3 | 29.4 | 41.2 | 41.5 | 40.8 | 25.2 | 25.2 | 21.6 | 20.6 | 21.6 |
| **SEC.v2** | | | | | | | | 100 | 98.1 | 97.4 | 30.9 | 28.3 | 40.4 | 40.8 | 40.1 | 24.1 | 24.1 | 20.9 | 20.2 | 20.9 |
| **SEC.v3** | | | | | | | | | 100 | 96.3 | 30.5 | 28.3 | 40.4 | 40.8 | 40.1 | 23.7 | 23.7 | 21.3 | 20.6 | 21.3 |
| **SEC.v4** | | | | | | | | | | 100 | 31.3 | 29 | 41.2 | 41.5 | 40.8 | 24.4 | 24.4 | 21.6 | 20.9 | 21.6 |
| **SED** | | | | | | | | | | | 100 | 52.9 | 25.8 | 26.2 | 26.2 | 33.1 | 32.7 | 27.5 | 27.1 | 28.2 |
| **SEE** | | | | | | | | | | | | 100 | 25.5 | 25.8 | 25.8 | 35.1 | 35.1 | 28.3 | 28.3 | 29 |
| **SEG.v1** | | | | | | | | | | | | | 100 | 97.3 | 96.9 | 25.5 | 25.5 | 23.5 | 23.5 | 23.5 |
| **SEG.v2** | | | | | | | | | | | | | | 100 | 98.8 | 25.5 | 25.5 | 23.8 | 23.8 | 23.8 |
| **SEG.v3** | | | | | | | | | | | | | | | 100 | 25.5 | 25.5 | 23.8 | 23.8 | 23.8 |
| **SEH.v1** | | | | | | | | | | | | | | | | 100 | 98.8 | 24.7 | 25.1 | 25.1 |
| **SEH.v2** | | | | | | | | | | | | | | | | | 100 | 24.7 | 25.1 | 25.1 |
| **SEI.v1** | | | | | | | | | | | | | | | | | | 100 | 93 | 96.7 |
| **SEI.v2** | | | | | | | | | | | | | | | | | | | 100 | 94.7 |
| **SEI.v3** | | | | | | | | | | | | | | | | | | | | 100 |

Reference sequences of the *se* gene variants of the VirulenceFinder database considered in this study can be accessed with the following accession IDs (the numbering of the gene variants can differ from the numbering in the database): *sea.v1* (AP009324.1), *sea.v2* (CP010526.1), *seb.v1* (CP007539.1), *seb.v2* (AB716349.1), *seb.v3* (AB716351.1), *seb.v4* (AB716352.1), *sec.v1* (AB084256.1), *sec.v2* (KF386012.1), *sec.v3* (KF729631.1), *sec.v4* (M28364.1), *sed* (M28521.1), *see* (M21319.1), *seg.v1* (CP001844.2), *seg.v2* (CP002388.1), *seg.v3* (AJ938182.1), *seh.v1* (BX571857.1), *seh.v2* (AY345144.1), *sei.v1* (BA000018.3), *sei.v2* (AJ938182.1), and *sei.v3* (CP002388.1). In regard to the subtypes of SEC, SEC1 is encoded by *sec.v2* and *sec.v3*, SEC2 by *sec.v4*, and SEC3 by *sec.v1*. Geneious version 2021.2 created by Biomatters. Available from http://www.geneious.com.

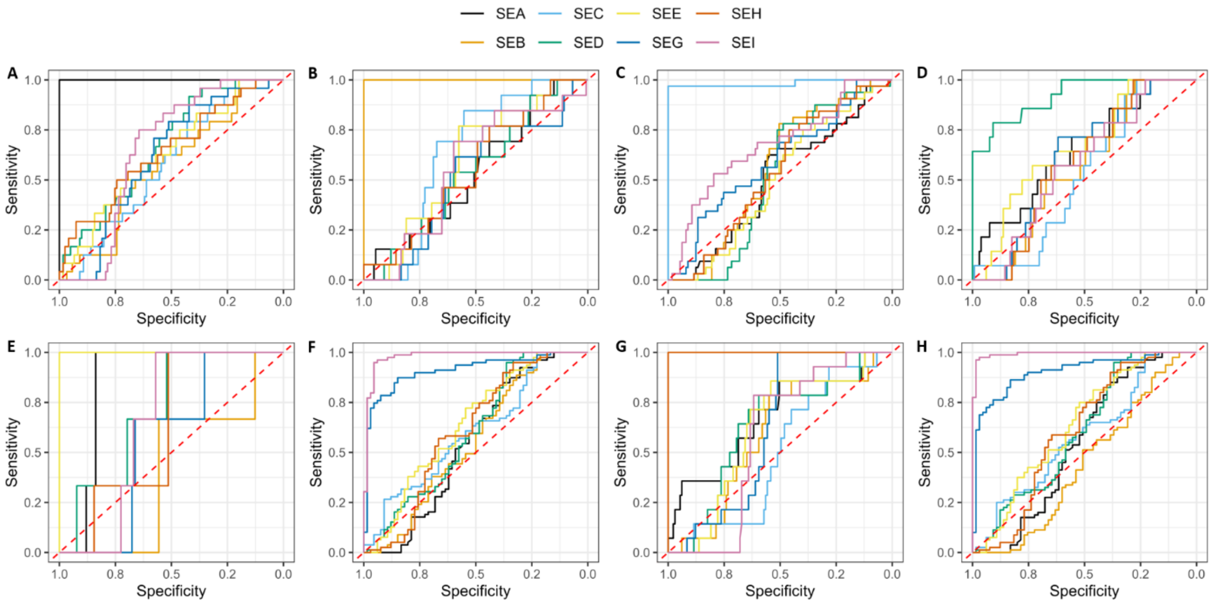


**Figure S9.** Sensitivity and specificity of the multiplex SIA detecting SEA to SEI. The multiplex SIA was validated using 145 culture supernatants, diluted 1:10 in 0.1% BSA/PBS, pH 7. Results were analysed with R (version 4.1.3). Sensitivity and specificity for each analyte A) SEA, B) SEB, C) SEC, D) SED, E) SEE, F) SEG, G) SEH, and H) SEI were evaluated through receiver operating characteristic curve analysis (pROC package, version 1.18.0), using WGS data as the gold standard which was compared to the multiplex SIA results.

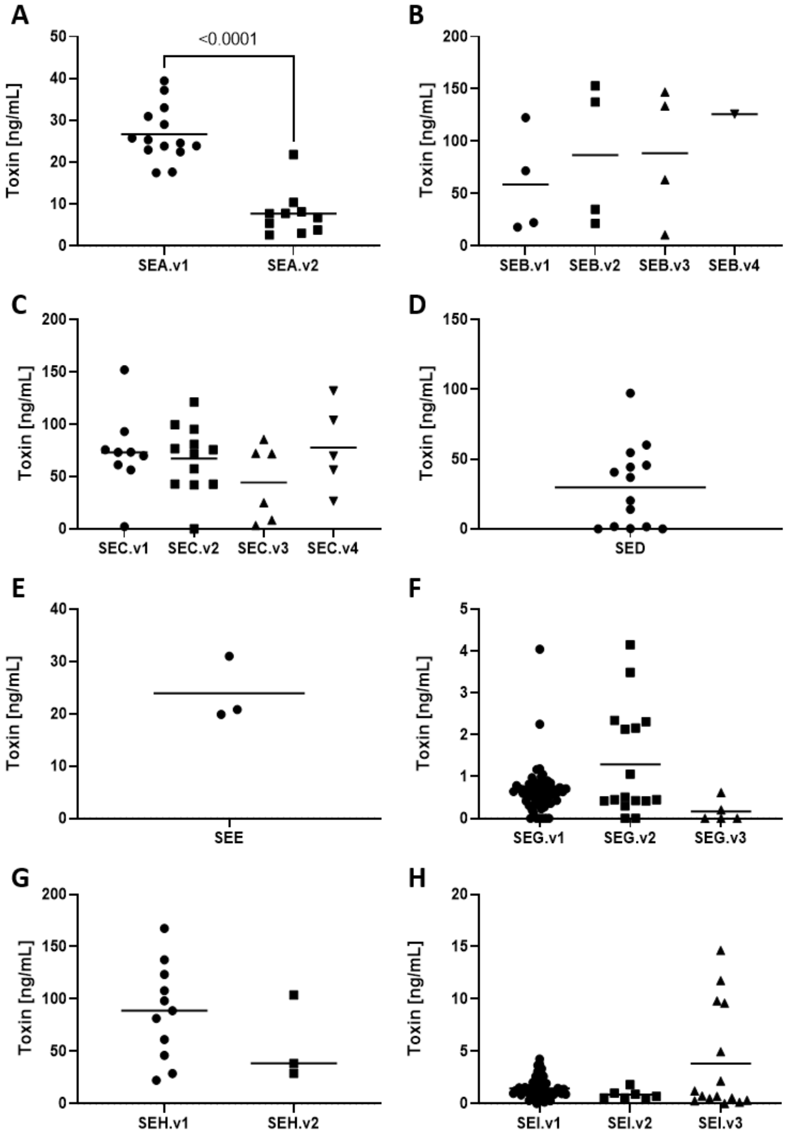


**Figure S10.** Overview of the estimated toxin concentration per SE type and variant in all culture supernatants derived from *sea*- to *sei*-genic strains measured by multiplex SIA. Capture mAbs were immobilised on beads and incubated with the supernatants, followed by addition of biotinylated mAb mixture and streptavidin-phycoerythrin. In this experiment ($n$ = 2), the results were extrapolated to the toxin dilution curve and are shown for A) SEA, B) SEB, C) SEC, D) SED, E) SEE, F) SEG, G) SEH, and H) SEI. Each concentration per variant and per sample is shown as a black dot for variant 1 (v1), black square for variant 2 (v2), upward-pointing triangle for variant 3 (v3), and downward-pointing triangle for variant 4 (v4). Only true positives and false negatives are shown. The mean concentration values across all target *se*-genic strains and variants are depicted as a line. The normal and lognormal distribution of the concentrations per SE variant was tested by Shapiro-Wilk test. If the difference of the mean values per SE type variant was statistically significant ($p$-value < 0.05) tested by $t$-test or one-way ANOVA, it is indicated by the connecting lines and the $p$-value given above.

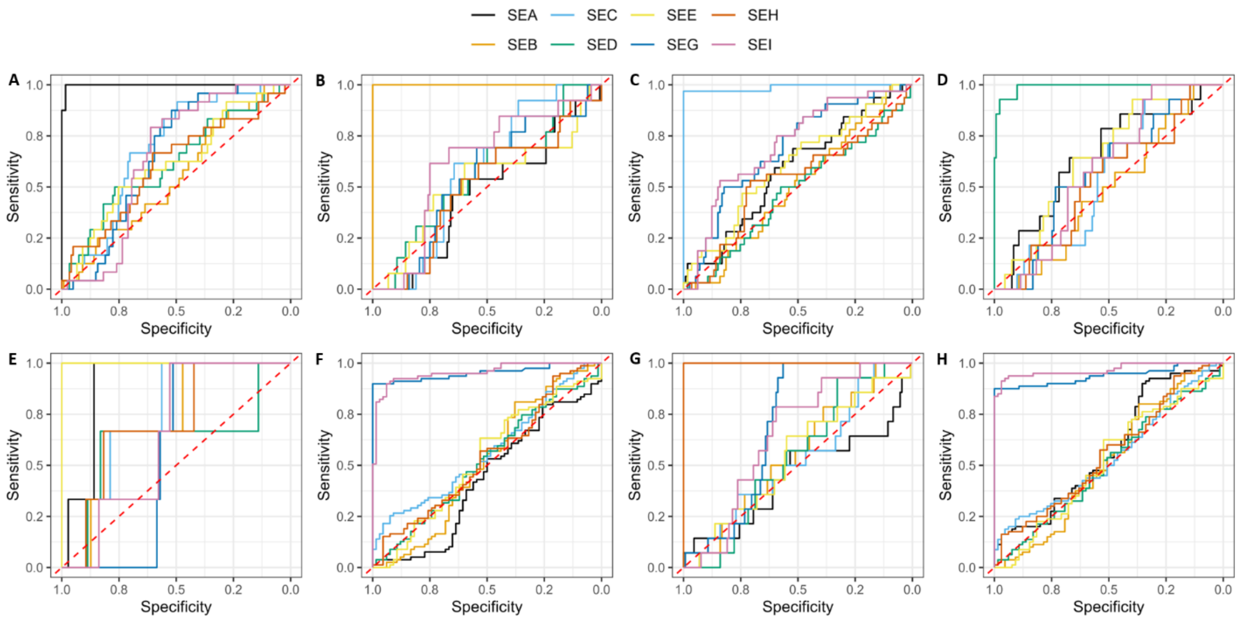


**Figure S11.** Sensitivity and specificity of the individual sandwich ELISAs detecting SEA to SEI. All eight sandwich ELISAs were validated using 145 culture supernatants, diluted 1:10 in 0.1% BSA/PBS, pH 7. Results were analysed with R (version 4.1.3). Sensitivity and specificity for each analyte A) SEA, B) SEB, C) SEC, D) SED, E) SEE, F) SEG, G) SEH, and H) SEI were evaluated through receiver operating curve analysis (pROC package, version 1.18.0), using WGS data as the gold standard.

**Table S3.** Overview of whole genome sequenced bacterial strains and the results of SE analysis via eight sandwich ELISAs in their liquid-culture supernatants.

| **Isolates** | | ***sea* / SEA** | | ***seb* / SEB** | | | | ***sec* / SEC** | | | | ***sed* /** | ***see* /** | ***seg* / SEG** | | | ***seh* / SEH** | | ***sei* / SEI** | | | **Other *se*** |
|---|---|---|---|---|---|---|---|---|---|---|---|---|---|---|---|---|---|---|---|---|---|---|
| **(species/ source)** | | **v1** | **v2** | **v1** | **v2** | **v3** | **v4** | **v1** | **v2** | **v3** | **v4** | **SED** | **SEE** | **v1** | **v2** | **v3** | **v1** | **v2** | **v1** | **v2** | **v3** | |
| 08S00573 | *se* | - | - | - | - | - | - | - | - | - | + | + | - | + | - | - | - | - | + | - | - | *selj, sel, sem, sen,* |
| (*S. aureus*/ BfR) | SE | - | | - | | | | + | | | | + | - | + | | | - | | + | | | *seo, ser, selx* |
| 08S00574 | *se* | - | - | - | - | - | - | - | + | - | - | - | - | - | - | + | - | + | - | - | + | *sel, sem, sen, seo,* |
| (*S. aureus*/ BfR) | SE | - | | - | | | | + | | | | - | - | - | | | + | | + | | | *selu, selx* |
| 08S00575 | *se* | - | - | - | - | - | - | - | - | - | - | - | + | - | - | - | - | - | - | - | - | *seq* |
| (*S. aureus*/ BfR) | SE | + | | - | | | | - | | | | - | + | - | | | - | | + | | | |
| 08S01548 | *se* | - | - | - | - | - | - | + | - | - | - | - | - | + | - | - | - | - | - | - | - | *sel, sem, sen, seo,* |
| (*S. aureus*/ BfR) | SE | - | | - | | | | + | | | | - | - | + | | | - | | - | | | *selu, selv, tst* |
| 09-00925 | *se* | - | - | - | - | - | - | - | - | - | - | - | - | + | - | - | - | - | + | - | - | *sem, sen, seo, selx* |
| (*S. aureus*/ RKI) | SE | - | | - | | | | - | | | | - | - | + | | | - | | + | | | |
| 09S00575 | *se* | - | - | - | - | - | - | - | - | - | - | - | + | - | - | - | - | - | - | - | - | *seq* |
| (*S. aureus*/ BfR) | SE | + | | - | | | | - | | | | - | + | - | | | - | | - | | | |
| 11S00617 | *se* | - | - | - | - | - | - | - | - | - | - | - | - | - | - | - | - | - | - | - | - | *sep* |
| (*S. aureus*/ BfR) | SE | - | | - | | | | - | | | | - | - | - | | | - | | - | | | |
| 12-01857 | *se* | - | - | - | - | - | - | - | - | - | - | - | - | + | - | - | - | - | + | - | - | *sem, sen, seo, selx* |
| (*S. aureus*/ RKI) | SE | - | | - | | | | - | | | | - | - | + | | | - | | + | | | |
| 12-03280 | *se* | - | + | - | - | - | - | - | - | - | - | - | - | - | + | - | - | - | - | - | + | *sem, sen, seo, selu,* |
| (*S. aureus*/ RKI) | SE | + | | - | | | | - | | | | - | - | + | | | - | | + | | | *tst* |
| 12S00034 | *se* | - | - | - | - | - | - | - | - | - | - | - | - | + | - | - | - | - | + | - | - | *sem, sen, seo, selx* |
| (*S. aureus*/ BfR) | SE | - | | - | | | | - | | | | - | - | + | | | - | | + | | | |
| 13-01702 | *se* | - | + | - | - | - | - | + | - | - | - | - | - | - | - | - | - | - | - | - | - | *sek, sel, seq, tst* |
| (*S. aureus*/ RKI) | SE | + | | - | | | | + | | | | - | - | - | | | - | | - | | | |

| Isolates (species/ source) | | *sea* / SEA | | *seb* / SEB | | | | *sec* / SEC | | | | *sed* / SED | *see* / SEE | *seg* / SEG | | | *seh* / SEH | | *sei* / SEI | | | Other *se* |
|---|---|---|---|---|---|---|---|---|---|---|---|---|---|---|---|---|---|---|---|---|---|---|
| | | v1 | v2 | v1 | v2 | v3 | v4 | v1 | v2 | v3 | v4 | SED | SEE | v1 | v2 | v3 | v1 | v2 | v1 | v2 | v3 | |
| 13-01965 | *se* | - | - | - | - | - | - | - | - | - | - | + | - | + | - | - | - | - | + | - | - | *selj, sem, sen, seo, sep, ser, selx* |
| (*S. aureus*/ RKI) | SE | - | | - | | | | - | | | | + | - | + | | | - | | + | | | |
| 13-03539 | *se* | - | - | - | - | - | - | - | + | - | - | - | - | + | - | - | - | - | + | - | - | *sel, sem, sen, seo, selx* |
| (*S. aureus*/ RKI) | SE | - | | - | | | | + | | | | - | - | + | | | - | | + | | | |
| 13-ST00233 | *se* | - | - | - | - | - | - | - | - | - | - | - | - | - | - | - | + | - | - | - | - | - |
| (*S. aureus*/ BfR) | SE | - | | - | | | | - | | | | - | - | - | | | + | | - | | | |
| 13-ST00641 | *se* | - | + | - | - | - | - | - | - | - | - | - | - | - | + | - | - | + | - | - | + | *sem, sen, seo, selu, selx, tst* |
| (*S. aureus*/ BfR) | SE | + | | - | | | | - | | | | - | - | - | | | + | | - | | | |
| 14-00366 | *se* | - | - | - | - | - | - | - | + | - | - | - | - | + | - | - | - | - | + | - | - | *sel, sem, sen, seo, selx* |
| (*S. aureus*/ RKI) | SE | - | | - | | | | + | | | | - | - | + | | | - | | + | | | |
| 14-00392 | *se* | - | - | - | - | - | - | - | + | - | - | - | - | + | - | - | - | - | + | - | - | *sel, sem, sen, seo, selx* |
| (*S. aureus*/ RKI) | SE | - | | - | | | | + | | | | - | - | + | | | - | | + | | | |
| 14-00471 | *se* | - | - | - | - | - | - | + | - | - | - | - | - | + | - | - | - | - | + | - | - | *sel, sem, sen, seo, selx, tst* |
| (*S. aureus*/ RKI) | SE | - | | - | | | | + | | | | - | - | + | | | - | | + | | | |
| 14-00475 | *se* | - | - | - | - | - | - | - | + | - | - | - | - | + | - | - | - | - | + | - | - | *sel, sem, sen, seo* |
| (*S. aureus*/ RKI) | SE | - | | - | | | | + | | | | - | - | + | | | - | | + | | | |
| 14-00507 | *se* | + | - | - | - | - | - | + | - | - | - | - | - | + | - | - | - | - | + | - | - | *sel, sem, sen, seo, selx, tst* |
| (*S. aureus*/ RKI) | SE | + | | - | | | | + | | | | - | - | + | | | - | | + | | | |
| 14-00511 | *se* | + | - | - | - | - | - | + | | - | - | - | - | + | - | - | - | - | + | - | - | *sel, sem, sen, seo, selx, tst* |
| (*S. aureus*/ RKI) | SE | + | | - | | | | + | | | | - | - | + | | | - | | + | | | |
| 14-00515 | *se* | - | - | - | - | - | - | - | - | - | - | - | - | + | - | - | - | - | + | - | - | *sem, sen, seo, selx* |
| (*S. aureus*/ RKI) | SE | - | | - | | | | - | | | | - | - | + | | | - | | + | | | |

| Isolates | | *sea* / SEA | | *seb* / SEB | | | | *sec* / SEC | | | | *sed* / | *see* / | *seg* / SEG | | | *seh* / SEH | | *sei* / SEI | | | Other *se* |
|---|---|---|---|---|---|---|---|---|---|---|---|---|---|---|---|---|---|---|---|---|---|---|
| (species/ source) | | v1 | v2 | v1 | v2 | v3 | v4 | v1 | v2 | v3 | v4 | SED | SEE | v1 | v2 | v3 | v1 | v2 | v1 | v2 | v3 | |
| 14-00552 | *se* | - | - | - | - | - | - | - | - | - | - | + | - | + | - | - | - | - | + | - | - | *sem, sen, seo, sep, sep, ser, selx* |
| (*S. aureus*/ RKI) | SE | - | | - | | | | - | | | | + | - | + | | | - | | + | | | |
| 14-00594 | *se* | - | - | - | - | - | - | - | - | - | - | - | - | - | - | +** | - | - | - | + | - | *sem, sen, seo, selu, selx, sely* |
| (*S. aureus*/ RKI) | SE | - | | - | | | | - | | | | - | - | - | | | - | | + | | | |
| 14-02531 | *se* | - | - | - | - | - | - | - | - | - | - | - | - | + | - | - | - | - | + | - | - | *sem, sen, seo, selx, tst* |
| (*S. aureus*/ RKI) | SE | - | | - | | | | - | | | | - | - | + | | | - | | + | | | |
| 14-02652 | *se* | - | - | - | - | - | - | - | - | - | - | - | - | + | - | - | - | - | + | - | - | *sem, seo* |
| (*S. aureus*/ RKI) | SE | - | | - | | | | - | | | | - | - | + | | | - | | + | | | |
| 14-ST00756 | *se* | - | - | - | - | - | - | - | - | - | - | - | - | + | - | - | - | - | + | - | - | - |
| (*S. aureus*/ BfR) | SE | - | | - | | | | - | | | | - | - | + | | | - | | + | | | |
| 14-ST00757 | *se* | - | + | - | - | - | - | - | - | + | - | - | - | - | - | - | - | - | - | - | - | *sel, tst* |
| (*S. aureus*/ BfR) | SE | + | | - | | | | + | | | | - | - | - | | | - | | - | | | |
| 15-02754 | *se* | - | - | - | + | - | - | - | - | - | - | - | - | - | + | - | - | - | - | + | - | *sem, sen, seo, selu, selx, sely* |
| (*S. aureus*/ RKI) | SE | - | | + | | | | - | | | | - | - | + | | | - | | + | | | |
| 15-02790 | *se* | - | - | - | - | - | - | - | - | - | - | - | - | + | | - | - | - | + | | | *sem, sen, seo, selx, tst* |
| (*S. aureus*/ RKI) | SE | - | | - | | | | - | | | | + | - | + | | | - | | + | | | |
| 15-ST00110 | *se* | - | - | - | - | - | - | - | - | - | - | - | - | - | - | - | - | - | - | - | + | *sem, sen, seo, selu, selx, sely* |
| (*S. aureus*/ BfR) | SE | - | | - | | | | - | | | | - | - | - | | | - | | + | | | |
| 15-ST00179 | *se* | - | - | - | - | - | - | - | - | - | - | - | - | - | - | - | - | - | - | - | - | - |
| (*S. aureus*/ BfR) | SE | - | | - | | | | - | | | | - | - | - | | | - | | - | | | |
| 15-ST00398 | *se* | - | - | - | - | - | - | - | - | - | - | - | - | - | - | - | - | - | - | - | - | *sep* |
| (*S. aureus*/ BfR) | SE | - | | - | | | | - | | | | - | - | - | | | - | | - | | | |

| Isolates (species/ source) | | *sea* / SEA | | *seb* / SEB | | | | *sec* / SEC | | | | *sed* / | *see* / | *seg* / SEG | | | *seh* / SEH | | *sei* / SEI | | | Other *se* |
|---|---|---|---|---|---|---|---|---|---|---|---|---|---|---|---|---|---|---|---|---|---|---|
| | | v1 | v2 | v1 | v2 | v3 | v4 | v1 | v2 | v3 | v4 | SED | SEE | v1 | v2 | v3 | v1 | v2 | v1 | v2 | v3 | |
| 15-ST00399 | *se* | - | - | - | - | - | - | - | - | - | - | - | - | + | - | - | - | - | + | - | - | *sem, sen, seo, selx* |
| (*S. aureus*/ BfR) | SE | - | | - | | | | - | | | | - | - | + | | | - | | + | | | |
| 15-ST00430 | *se* | - | - | - | - | + | - | - | - | - | - | - | - | - | - | - | - | - | - | - | - | - |
| (*S. aureus*/ BfR) | SE | - | | + | | | | - | | | | - | - | - | | | - | | - | | | |
| 15-ST00439 | *se* | - | + | - | - | - | - | - | - | - | - | - | - | - | - | - | - | - | - | - | - | - |
| (*S. aureus*/ BfR) | SE | + | | - | | | | - | | | | - | - | - | | | - | | - | | | |
| 15-ST00440 | *se* | - | - | - | - | - | - | - | - | + | - | - | - | - | - | - | - | - | - | - | - | *sel, tst* |
| (*S. aureus*/ BfR) | SE | - | | - | | | | + | | | | - | - | - | | | - | | - | | | |
| 15-ST00441 | *se* | - | - | - | - | - | - | - | - | + | - | - | - | - | - | - | - | - | - | - | - | *sel, tst* |
| (*S. aureus*/ BfR) | SE | - | | - | | | | + | | | | - | - | - | | | - | | - | | | |
| 15-ST00521 | *se* | - | - | - | - | - | - | - | - | + | - | - | - | - | - | - | - | - | - | - | - | *sel, tst* |
| (*S. aureus*/ BfR) | SE | - | | - | | | | + | | | | + | - | - | | | - | | + | | | |
| 15-ST00539 | *se* | - | - | + | - | - | - | - | - | - | - | - | - | - | - | - | - | - | - | - | - | *sep* |
| (*S. aureus*/ BfR) | SE | - | | + | | | | - | | | | - | - | - | | | - | | - | | | |
| 15-ST00574 | *se* | - | - | - | - | - | - | - | - | - | - | - | - | - | + | - | - | - | - | - | + | *sem, sen, seo, selu, selx* |
| (*S. aureus*/ BfR) | SE | - | | - | | | | - | | | | - | - | + | | | - | | + | | | |
| 15-ST00670 | *se* | - | - | - | - | - | - | - | - | - | - | - | - | + | - | - | - | - | + | - | - | *sem, sen, seo, selx* |
| (*S. aureus*/ BfR) | SE | - | | - | | | | - | | | | - | - | + | | | - | | + | | | |
| 16-00175 | *se* | - | - | - | - | - | - | - | - | - | - | +* | - | + | - | - | - | - | + | - | - | *sem, sen, seo, selx* |
| (*S. aureus*/ RKI) | SE | - | | - | | | | - | | | | - | - | + | | | - | | + | | | |
| 16-00532 | *se* | - | - | - | - | - | - | - | - | - | - | +* | - | + | - | - | - | - | + | - | - | *sem, sen, seo, selx* |
| (*S. aureus*/ RKI) | SE | - | | - | | | | - | | | | - | - | + | | | - | | + | | | |

| Isolates (species/ source) | | *sea* / SEA v1 | v2 | *seb* / SEB v1 | v2 | v3 | v4 | *sec* / SEC v1 | v2 | v3 | v4 | *sed* / SED | *see* / SEE | *seg* / SEG v1 | v2 | v3 | *seh* / SEH v1 | v2 | *sei* / SEI v1 | v2 | v3 | Other *se* |
|---|---|---|---|---|---|---|---|---|---|---|---|---|---|---|---|---|---|---|---|---|---|---|
| 16-01004 | *se* | - | - | - | - | - | - | - | - | - | - | - | - | - | - | - | + | - | - | - | - | *selx* |
| (*S. aureus*/ RKI) | SE | - | | - | | | | - | | | | - | - | - | | | + | | - | | | |
| 16-01130 | *se* | - | - | - | - | - | - | - | - | - | - | - | - | + | - | - | - | - | + | - | - | *sem, sen, seo, selx* |
| (*S. aureus*/ RKI) | SE | - | | - | | | | - | | | | - | - | + | | | - | | + | | | |
| 16-01169 | *se* | - | - | - | - | - | - | - | + | - | - | - | - | + | - | - | - | - | + | - | - | *sel, sem, sen, seo, selx* |
| (*S. aureus*/ RKI) | SE | - | | - | | | | + | | | | - | - | + | | | - | | + | | | |
| 16-01327 | *se* | - | - | - | - | - | - | - | - | - | - | - | - | - | + | - | - | - | - | + | - | *sem, sen, seo, selu* |
| (*S. aureus*/ RKI) | SE | - | | - | | | | - | | | | - | - | + | | | - | | + | | | |
| 16-01370 | *se* | - | - | - | - | - | - | - | + | - | - | - | - | + | | - | - | - | + | - | - | *sel, sem, sen, selx* |
| (*S. aureus*/ RKI) | SE | - | | - | | | | + | | | | - | - | + | | | - | | + | | | |
| 16-01940 | *se* | - | - | - | - | - | - | - | - | - | - | - | - | - | + | - | - | - | - | - | + | *sem, sen, seo, selu, tst* |
| (*S. aureus*/ RKI) | SE | - | | - | | | | - | | | | - | - | + | | | - | | + | | | |
| 16-02151 | *se* | - | - | - | - | - | - | - | - | - | + | - | - | + | - | - | - | - | + | - | - | *selj, sel, sem, sen, seo* |
| (*S. aureus*/ RKI) | SE | - | | - | | | | + | | | | - | - | + | | | - | | + | | | |
| 16-02200 | *se* | - | - | - | - | - | + | - | - | - | - | - | - | + | - | - | - | - | + | - | - | *sem, sen, seo, selx, sely* |
| (*S. aureus*/ RKI) | SE | - | | + | | | | - | | | | - | - | + | | | - | | - | | | |
| 16-03400 | *se* | - | - | - | - | - | - | - | - | - | - | - | - | - | + | - | - | - | - | - | + | *sem, sen, seo, selu, tst* |
| (*S. aureus*/ RKI) | SE | - | | - | | | | - | | | | - | - | + | | | - | | + | | | |
| 16-ST00051 | *se* | + | - | - | - | - | - | - | - | - | - | - | - | - | - | - | - | - | - | - | - | - |
| (*S. aureus*/ BfR) | SE | + | | - | | | | - | | | | - | - | - | | | - | | - | | | |
| 16-ST00052 | *se* | - | - | - | + | - | - | - | - | - | - | - | - | + | - | - | - | - | + | - | - | *sem, sen, seo, selx* |
| (*S. aureus*/ BfR) | SE | - | | + | | | | - | | | | - | - | + | | | - | | + | | | |

| Isolates (species/ source) | | *sea* / SEA | | *seb* / SEB | | | | *sec* / SEC | | | | *sed* / SED | *see* / SEE | *seg* / SEG | | | *seh* / SEH | | *sei* / SEI | | | Other *se* |
|---|---|---|---|---|---|---|---|---|---|---|---|---|---|---|---|---|---|---|---|---|---|---|
| | | v1 | v2 | v1 | v2 | v3 | v4 | v1 | v2 | v3 | v4 | | | v1 | v2 | v3 | v1 | v2 | v1 | v2 | v3 | |
| 16-ST00082 | *se* | - | - | - | - | - | - | - | - | - | - | - | - | - | - | - | - | - | - | - | - | - |
| (*S. aureus*/ BfR) | SE | - | | - | | | | - | | | | - | - | - | | | - | | - | | | |
| 16-ST00085 | *se* | - | - | - | - | - | - | - | - | - | - | + | - | + | - | - | - | - | + | - | - | *selj, sem, sen, seo, ser, selx* |
| (*S. aureus*/ BfR) | SE | - | | - | | | | - | | | | + | - | + | | | - | | + | | | |
| 16-ST00091 | *se* | - | - | - | - | - | - | - | + | - | - | - | - | + | - | - | - | - | + | - | - | *sel, sem, sen, seo, selx* |
| (*S. aureus*/ BfR) | SE | - | | - | | | | + | | | | - | - | + | | | - | | + | | | |
| 16-ST00139 | *se* | - | - | - | - | - | - | - | - | - | - | - | - | - | - | - | - | - | - | - | - | - |
| (*S. aureus*/ BfR) | SE | - | | - | | | | - | | | | - | - | - | | | - | | - | | | |
| 16-ST00147 | *se* | + | - | - | - | - | - | - | - | - | - | - | - | - | - | - | - | - | - | - | - | - |
| (*S. aureus*/ BfR) | SE | + | | - | | | | - | | | | - | - | - | | | - | | - | | | |
| 16-ST00159 | *se* | - | - | - | + | - | - | - | - | - | - | - | - | - | - | - | - | - | - | - | - | - |
| (*S. aureus*/ BfR) | SE | - | | + | | | | - | | | | - | - | - | | | - | | - | | | |
| 16-ST00336 | *se* | - | - | - | - | - | - | - | - | - | - | - | - | + | - | - | - | - | + | - | - | *sem, sen, seo, selx, sely* |
| (*S. aureus*/ BfR) | SE | - | | - | | | | - | | | | - | - | + | | | - | | + | | | |
| 16-ST00351 | *se* | - | + | - | - | - | - | - | - | + | - | - | - | - | - | - | - | - | - | - | - | *sel, tst* |
| (*S. aureus*/ BfR) | SE | + | | - | | | | + | | | | - | - | - | | | - | | - | | | |
| 16-ST00352 | *se* | - | - | - | - | - | - | - | - | + | - | - | - | - | - | - | - | - | - | - | - | *sel, tst* |
| (*S. aureus*/ BfR) | SE | - | | - | | | | + | | | | - | - | - | | | - | | - | | | |
| 16-ST00416-1 | *se* | - | - | - | - | - | - | - | - | - | - | - | - | - | - | - | - | - | - | - | - | *sep* |
| (*S. aureus*/ BfR) | SE | - | | - | | | | - | | | | - | - | - | | | - | | - | | | |
| 16-ST00419-1 | *se* | - | - | - | - | - | - | - | - | - | - | - | - | - | - | - | - | - | - | - | - | *tst* |
| (*S. aureus*/ BfR) | SE | - | | - | | | | - | | | | - | - | - | | | - | | - | | | |

| **Isolates** | | ***sea* / SEA** | | ***seb* / SEB** | | | | ***sec* / SEC** | | | | ***sed* /** | ***see* /** | ***seg* / SEG** | | | ***seh* / SEH** | | ***sei* / SEI** | | | **Other *se*** |
|---|---|---|---|---|---|---|---|---|---|---|---|---|---|---|---|---|---|---|---|---|---|---|
| **(species/ source)** | | **v1** | **v2** | **v1** | **v2** | **v3** | **v4** | **v1** | **v2** | **v3** | **v4** | **SED** | **SEE** | **v1** | **v2** | **v3** | **v1** | **v2** | **v1** | **v2** | **v3** | |
| 16-ST00419 | *se* | - | - | + | - | - | - | - | - | - | - | - | - | - | - | - | - | - | - | - | - | *sel, selx* |
| (*S. aureus*/ BfR) | SE | - | | + | | | | - | | | | - | - | - | | | - | | - | | | |
| 16-ST00423 | *se* | - | - | - | + | - | - | - | - | - | + | - | - | + | - | - | - | - | + | - | - | *sel, sem, sen, seo, selx* |
| (*S. aureus*/ BfR) | SE | - | | + | | | | + | | | | - | - | + | | | - | | + | | | |
| 16-ST00436 | *se* | - | - | - | - | - | - | - | - | - | - | - | - | + | - | - | - | - | + | - | - | *sem, sen, seo, selx, sely* |
| (*S. aureus*/ BfR) | SE | - | | - | | | | - | | | | - | - | + | | | - | | + | | | |
| 16-ST00436-2 | *se* | - | - | - | - | - | - | - | - | - | - | - | - | + | - | - | - | - | + | - | - | *sem, sen, seo, selx, sely* |
| (*S. aureus*/ BfR) | SE | - | | - | | | | - | | | | - | - | + | | | - | | + | | | |
| 16-ST00438-2 | *se* | - | - | - | - | - | - | - | - | - | - | + | - | + | - | - | - | - | + | - | - | *selj, sem, sen, seo, sep, ser, selx* |
| (*S. aureus*/ BfR) | SE | - | | - | | | | - | | | | + | - | + | | | - | | + | | | |
| 16-ST00582 | *se* | + | - | - | - | - | - | - | - | - | - | - | - | - | - | - | + | - | - | - | - | - |
| (*S. aureus*/ BfR) | SE | + | | - | | | | - | | | | - | - | - | | | + | | - | | | |
| 16-ST00732 | *se* | - | - | - | - | - | - | - | - | - | + | - | - | - | - | - | - | - | - | - | - | *sel* |
| (*S. aureus*/ BfR) | SE | - | | - | | | | + | | | | - | - | - | | | - | | + | | | |
| 17-01004 | *se* | - | - | - | - | - | - | - | - | - | - | - | - | - | - | - | + | | - | - | - | *selx* |
| (*S. aureus*/ RKI) | SE | - | | - | | | | - | | | | - | - | - | | | + | | - | | | |
| 17-ST00010 | *se* | - | + | - | - | - | - | - | - | - | - | - | - | - | - | - | - | - | - | - | - | - |
| (*S. aureus*/ BfR) | SE | + | | - | | | | - | | | | - | - | - | | | - | | - | | | |
| 17-ST00011 | *se* | - | - | - | - | - | - | + | - | - | - | - | - | + | - | - | - | - | + | - | - | *sel, sem, sen, seo, sep, selx, tst* |
| (*S. aureus*/ BfR) | SE | - | | - | | | | + | | | | - | - | + | | | - | | + | | | |
| 17-ST00026 | *se* | - | - | - | - | - | - | - | - | - | - | + | - | - | - | - | - | - | - | - | - | *selj, ser* |
| (*S. aureus*/ BfR) | SE | - | | - | | | | - | | | | + | - | - | | | - | | - | | | |

| Isolates (species/ source) | | *sea* / SEA | | *seb* / SEB | | | | *sec* / SEC | | | | *sed* / SED | *see* / SEE | *seg* / SEG | | | *seh* / SEH | | *sei* / SEI | | | Other *se* |
|---|---|---|---|---|---|---|---|---|---|---|---|---|---|---|---|---|---|---|---|---|---|---|
| | | v1 | v2 | v1 | v2 | v3 | v4 | v1 | v2 | v3 | v4 | | | v1 | v2 | v3 | v1 | v2 | v1 | v2 | v3 | |
| 17-ST00128 | *se* | + | - | - | - | - | - | - | - | - | - | + | - | - | - | - | - | - | - | - | - | *selj, sek, seq, ser* |
| (*S. aureus*/ BfR) | SE | + | | - | | | | - | | | | + | - | - | | | - | | - | | | |
| 17-ST00292 | *se* | - | - | - | - | - | - | + | - | - | - | - | - | + | - | - | - | - | + | - | - | *sel, sem, sen, seo, selx, tst* |
| (*S. aureus*/ BfR) | SE | - | | - | | | | + | | | | - | - | + | | | - | | + | | | |
| 17-ST00351 | *se* | + | - | - | - | - | - | - | - | - | - | + | - | - | - | - | - | - | - | - | - | *selj, ser* |
| (*S. aureus*/ BfR) | SE | + | | - | | | | - | | | | + | - | - | | | - | | - | | | |
| 17-ST00452 | *se* | - | - | - | - | - | - | - | - | - | - | - | - | - | - | - | - | - | - | - | - | - |
| (*S. aureus*/ BfR) | SE | - | | - | | | | - | | | | - | - | - | | | - | | - | | | |
| 18-01509 | *se* | - | - | + | - | - | - | - | - | - | - | - | - | - | - | - | - | - | - | - | - | *sek, seq, selx, sely* |
| (*S. aureus*/ RKI) | SE | - | | + | | | | - | | | | - | - | - | | | - | | - | | | |
| 18-01582 | *se* | - | - | - | - | - | - | - | - | - | - | - | - | + | - | - | - | - | + | - | - | *selj, sem, sen, seo, selx, tst* |
| (*S. aureus*/ RKI) | SE | - | | - | | | | - | | | | - | - | + | | | - | | + | | | |
| 18-02513 | *se* | - | - | - | - | - | - | - | - | - | - | - | - | + | - | - | - | - | + | - | - | *sem, sen, seo, selx, tst* |
| (*S. aureus*/ RKI) | SE | - | | - | | | | - | | | | - | - | - | | | - | | + | | | |
| 18-02689 | *se* | - | - | - | - | - | - | - | + | - | - | - | - | - | - | + | - | - | - | + | - | *sel, sem, sen, seo, selu, selx, tst* |
| (*S. aureus*/ RKI) | SE | - | | - | | | | + | | | | - | - | + | | | - | | + | | | |
| 18-02690 | *se* | - | + | - | - | - | - | + | - | - | - | - | + | - | - | - | - | - | - | - | - | *sek, sel, seq* |
| (*S. aureus*/ RKI) | SE | + | | - | | | | + | | | | - | + | - | | | - | | - | | | |
| 18-ST00090 | *se* | - | - | - | - | - | - | - | - | - | - | - | - | - | - | +** | - | + | - | - | + | *sem, sen, seo, selu, selx, sely* |
| (*S. aureus*/ BfR) | SE | - | | - | | | | - | | | | - | - | - | | | + | | + | | | |
| 18-ST00093 | *se* | - | + | - | - | - | - | - | - | - | - | - | - | - | + | - | - | - | - | - | + | *sem, sen, seo, selu, selx, tst* |
| (*S. aureus*/ BfR) | SE | + | | - | | | | - | | | | - | - | + | | | - | | + | | | |

| Isolates | | *sea* / SEA | | *seb* / SEB | | | | *sec* / SEC | | | | *sed* / | *see* / | *seg* / SEG | | | *seh* / SEH | | *sei* / SEI | | | Other *se* |
|---|---|---|---|---|---|---|---|---|---|---|---|---|---|---|---|---|---|---|---|---|---|---|
| (species/ source) | | v1 | v2 | v1 | v2 | v3 | v4 | v1 | v2 | v3 | v4 | SED | SEE | v1 | v2 | v3 | v1 | v2 | v1 | v2 | v3 | |
| 18-ST00095 | *se* | + | - | - | - | - | - | - | - | - | - | +** | - | - | - | - | - | - | - | - | - | *selj, ser* |
| (*S. aureus*/ BfR) | SE | + | | - | | | | - | | | | - | - | - | | | - | | - | | | |
| 18-ST00096 | *se* | + | - | - | - | - | - | - | - | - | - | | - | - | - | - | - | - | - | - | - | *sek, seq* |
| (*S. aureus*/ BfR) | SE | + | | - | | | | - | | | | - | - | - | | | - | | - | | | |
| 18-ST00169 | *se* | + | - | - | - | + | - | - | - | - | - | - | - | - | - | - | - | - | - | - | - | *selx* |
| (*S. aureus*/ BfR) | SE | + | | + | | | | - | | | | - | - | - | | | - | | - | | | |
| 18-ST00293 | *se* | - | - | - | - | - | - | - | - | - | - | - | - | - | - | - | + | - | - | - | - | - |
| (*S. aureus*/ BfR) | SE | - | | - | | | | - | | | | - | - | - | | | + | | - | | | |
| 18-ST00474 | *se* | - | - | - | - | - | - | - | - | - | - | - | - | - | + | - | - | - | - | + | - | *sem, sen, seo, selu, selx* |
| (*S. aureus*/ BfR) | SE | - | | - | | | | - | | | | - | - | + | | | - | | + | | | |
| 18-ST00526 | *se* | - | - | - | - | - | - | - | - | - | - | - | - | - | - | - | - | - | - | - | - | - |
| (*S. aureus*/ BfR) | SE | - | | - | | | | - | | | | - | - | - | | | - | | - | | | |
| 18-ST00599-2 | *se* | + | - | - | - | - | - | - | - | - | - | - | - | - | - | - | + | - | - | - | - | *sek, seq* |
| (*S. aureus*/ BfR) | SE | + | | - | | | | - | | | | - | - | - | | | + | | - | | | |
| 19-00144 | *se* | + | - | - | - | + | - | - | - | - | - | - | - | - | - | - | + | - | - | - | - | *sek, seq, selx* |
| (*S. aureus*/ RKI) | SE | + | | + | | | | - | | | | - | - | - | | | + | | - | | | |
| 19-00456 | *se* | - | - | - | - | - | - | - | - | - | - | - | - | + | - | - | - | - | + | - | - | *sem, sen, seo, selx, tst* |
| (*S. aureus*/ RKI) | SE | - | | - | | | | - | | | | - | - | + | | | - | | + | | | |
| 19-01991 | *se* | - | - | - | - | - | - | - | - | - | - | - | - | - | + | - | - | - | - | - | + | *sem, sen, seo, selu* |
| (*S. aureus*/ RKI) | SE | - | | - | | | | - | | | | - | - | + | | | - | | + | | | |
| 19-02864 | *se* | - | - | - | - | - | - | - | - | - | - | - | - | - | + | - | - | - | - | - | + | *sem, sen, seo* |
| (*S. aureus*/ RKI) | SE | - | | - | | | | - | | | | - | - | + | | | - | | + | | | |

| Isolates (species/ source) | | *sea* / SEA | | *seb* / SEB | | | | *sec* / SEC | | | | *sed* / SED | *see* / SEE | *seg* / SEG | | | *seh* / SEH | | *sei* / SEI | | | Other *se* |
|---|---|---|---|---|---|---|---|---|---|---|---|---|---|---|---|---|---|---|---|---|---|---|
| | | **v1** | **v2** | **v1** | **v2** | **v3** | **v4** | **v1** | **v2** | **v3** | **v4** | **SED** | **SEE** | **v1** | **v2** | **v3** | **v1** | **v2** | **v1** | **v2** | **v3** | |
| 19-02865 | *se* | - | - | - | - | - | - | - | - | - | - | - | - | - | + | - | - | - | - | - | + | *sem, sen* |
| (*S. aureus*/ RKI) | SE | - | | - | | | | - | | | | - | - | + | | | - | | + | | | |
| 19-02901 | *se* | - | - | - | - | - | - | - | - | - | - | - | - | - | + | - | - | - | - | - | + | *sem, sen, seo* |
| (*S. aureus*/ RKI) | SE | - | | - | | | | - | | | | - | - | + | | | - | | + | | | |
| 19-02911 | *se* | - | - | - | - | - | - | - | - | - | - | - | - | - | + | - | - | - | - | - | + | *sem, seo, selu* |
| (*S. aureus*/ RKI) | SE | - | | - | | | | - | | | | - | - | + | | | - | | + | | | |
| 19-03223 | *se* | - | - | + | - | - | - | - | - | - | - | - | - | - | - | - | - | - | - | - | - | *sep, selx* |
| (*S. aureus*/ RKI) | SE | - | | + | | | | - | | | | - | - | - | | | - | | - | | | |
| 19-03502 | *se* | - | - | - | - | - | - | - | - | - | - | - | - | - | + | - | - | - | - | - | + | *sem, sen, seo, selu* |
| (*S. aureus*/ RKI) | SE | - | | - | | | | - | | | | - | - | + | | | - | | - | | | |
| 19-ST00108 | *se* | - | - | - | - | - | - | - | - | - | - | - | - | - | - | +** | - | + | - | - | + | *sem, sen, seo, selu, selx, sely* |
| (*S. aureus*/ BfR) | SE | - | | - | | | | - | | | | - | - | - | | | + | | + | | | |
| 19-ST00670 | *se* | - | - | - | - | - | - | - | - | - | - | - | - | - | - | - | - | - | - | - | - | - |
| (*S. aureus*/ BfR) | SE | - | | - | | | | - | | | | - | - | - | | | - | | - | | | |
| 19-ST00949 | *se* | - | - | - | - | - | - | - | - | - | - | - | - | - | - | - | - | - | - | - | - | - |
| (*S. aureus*/ BfR) | SE | - | | - | | | | - | | | | - | - | - | | | - | | - | | | |
| 19-ST00963 | *se* | - | + | - | - | - | - | - | - | - | - | - | - | + | - | - | - | - | + | - | - | *sem, sen, seo, selx* |
| (*S. aureus*/ BfR) | SE | + | | - | | | | - | | | | - | - | + | | | - | | + | | | |
| 20-00159 | *se* | - | - | - | - | - | - | - | - | - | - | - | - | + | - | - | - | - | + | - | - | *sem, sen, seo, selx, sely* |
| (*S. aureus*/ RKI) | SE | - | | - | | | | - | | | | - | - | - | | | - | | + | | | |
| 20-00891 | *se* | - | - | - | - | - | - | - | - | - | - | - | - | + | - | - | - | - | + | - | - | *sem, sen, seo, sep, selx* |
| (*S. aureus*/ RKI) | SE | - | | - | | | | - | | | | - | - | + | | | - | | + | | | |

| Isolates | | *sea* / SEA | | *seb* / SEB | | | | *sec* / SEC | | | | *sed* / | *see* / | *seg* / SEG | | | *seh* / SEH | | *sei* / SEI | | | Other *se* |
|---|---|---|---|---|---|---|---|---|---|---|---|---|---|---|---|---|---|---|---|---|---|---|
| (species/ source) | | v1 | v2 | v1 | v2 | v3 | v4 | v1 | v2 | v3 | v4 | SED | SEE | v1 | v2 | v3 | v1 | v2 | v1 | v2 | v3 | |
| 20-00967 | *se* | - | - | - | - | + | - | - | - | - | - | - | - | - | - | - | - | - | - | + | - | *sek, sem, sen, seo, seq, selu, selx* |
| (*S. aureus*/ RKI) | SE | - | | + | | | | - | | | | - | - | - | | | - | | + | | | |
| 20-01064 | *se* | - | - | - | - | - | - | - | + | - | - | - | - | + | - | - | - | - | + | - | - | *sel, sem, sen, seo, selx* |
| (*S. aureus*/ RKI) | SE | - | | - | | | | - | | | | - | - | + | | | - | | + | | | |
| 20-01310 | *se* | - | - | - | - | - | - | - | - | - | - | - | - | + | - | - | - | - | + | - | - | *sem, sen, seo, selx, sely* |
| (*S. aureus*/ RKI) | SE | - | | - | | | | - | | | | - | - | + | | | - | | + | | | |
| 20-01793 | *se* | - | - | - | - | - | - | - | - | - | - | - | - | + | - | - | - | - | + | - | - | *sem, sen, seo, selx* |
| (*S. aureus*/ RKI) | SE | - | | - | | | | - | | | | - | - | - | | | - | | + | | | |
| 20-02215 | *se* | - | - | - | - | - | - | - | + | - | - | - | - | + | - | - | - | - | + | - | - | *sel, sem, sen, seo, selx* |
| (*S. aureus*/ RKI) | SE | - | | - | | | | + | | | | - | - | + | | | - | | + | | | |
| 20-02276 | *se* | - | - | - | - | - | - | - | + | - | - | - | - | + | - | - | - | - | - | - | - | *sel, sem, sen, seo, selx* |
| (*S. aureus*/ RKI) | SE | - | | - | | | | + | | | | - | - | + | | | - | | - | | | |
| 20-ST00005 | *se* | - | - | - | - | - | - | - | - | - | - | - | - | - | - | - | - | - | - | - | - | - |
| (*S. aureus*/ BfR) | SE | - | | - | | | | - | | | | - | - | - | | | - | | - | | | |
| 20-ST00335 | *se* | - | - | - | - | - | - | - | - | - | - | - | - | + | - | - | - | - | + | - | - | *selj, sem, sen, seo, sep, ser, ses, set, selx* |
| (*S. aureus*/ BfR) | SE | - | | - | | | | - | | | | - | - | + | | | - | | + | | | |
| 20-ST00363 | *se* | - | - | - | - | - | - | - | - | - | - | - | - | - | - | - | - | - | - | - | - | - |
| (*S. aureus*/ BfR) | SE | - | | - | | | | - | | | | - | - | - | | | - | | - | | | |
| 20-ST00418 | *se* | + | - | - | - | - | - | - | - | - | - | - | - | - | - | - | + | - | - | - | - | *sek, seq* |
| (*S. aureus*/ BfR) | SE | + | | - | | | | - | | | | - | - | - | | | + | | - | | | |
| 20-ST00441 | *se* | - | - | - | - | - | - | - | - | - | - | - | - | + | - | - | - | - | + | - | - | *sem, sen, seo, selx* |
| (*S. aureus*/ BfR) | SE | - | | - | | | | - | | | | - | - | + | | | - | | + | | | |

| Isolates (species/ source) | | *sea* / SEA | | *seb* / SEB | | | | *sec* / SEC | | | | *sed* / SED | *see* / SEE | *seg* / SEG | | | *seh* / SEH | | *sei* / SEI | | | Other *se* |
|---|---|---|---|---|---|---|---|---|---|---|---|---|---|---|---|---|---|---|---|---|---|---|
| | | v1 | v2 | v1 | v2 | v3 | v4 | v1 | v2 | v3 | v4 | | | v1 | v2 | v3 | v1 | v2 | v1 | v2 | v3 | |
| 20-ST00482 | *se* | - | - | - | - | - | - | - | - | - | - | - | - | - | - | - | - | + | - | - | - | - |
| (*S. aureus*/ BfR) | SE | - | | - | | | | - | | | | - | - | - | | | + | | - | | | |
| 20-ST00486 | *se* | - | - | - | - | - | - | - | - | - | - | - | - | - | - | - | - | - | - | - | - | - |
| (*S. aureus*/ BfR) | SE | - | | - | | | | - | | | | - | - | - | | | - | | - | | | |
| 21-00412 | *se* | - | - | - | - | - | - | - | - | - | - | - | - | + | - | - | - | - | + | - | - | *sem, sen, seo, selx* |
| (*S. aureus*/ RKI) | SE | - | | - | | | | - | | | | - | - | + | | | - | | - | | | |
| 21-00492 | *se* | - | - | - | - | - | - | - | - | - | + | - | - | + | - | - | - | - | + | - | - | *sel, sem, sen, seo, selx, sely* |
| (*S. aureus*/ RKI) | SE | - | | - | | | | + | | | | - | - | + | | | - | | + | | | |
| 21-01535 | *se* | - | - | - | - | - | - | - | - | - | - | +** | - | + | - | - | - | - | + | - | - | *selj, sem, sen, seo, sep, ser, selx* |
| (*S. aureus*/ RKI) | SE | - | | - | | | | - | | | | + | - | + | | | - | | + | | | |
| 21-01536 | *se* | - | - | - | - | - | - | - | - | - | - | - | - | + | - | - | - | - | + | - | - | *sem, sen, seo, selx* |
| (*S. aureus*/ RKI) | SE | - | | - | | | | - | | | | - | - | + | | | - | | + | | | |
| 21-01671 | *se* | - | - | - | - | - | - | - | - | - | - | - | - | - | - | - | + | - | - | - | - | *selx* |
| (*S. aureus*/ RKI) | SE | - | | - | | | | - | | | | - | - | - | | | + | | - | | | |
| 21-01745 | *se* | - | - | - | - | - | - | - | - | - | - | + | - | + | - | - | - | - | + | - | - | *selj, sem, sen, seo, sep, ser, selx* |
| (*S. aureus*/ RKI) | SE | - | | - | | | | - | | | | + | - | + | | | - | | + | | | |
| 21-02529 | *se* | - | - | - | - | - | - | + | - | - | - | - | - | + | - | - | - | - | + | - | - | *sel, sem, sen, seo, selx, tst* |
| (*S. aureus*/ RKI) | SE | - | | - | | | | + | | | | - | - | + | | | - | | + | | | |
| 21-02538-2 | *se* | - | - | - | - | - | - | - | - | - | - | - | - | + | - | - | - | - | + | - | - | *sem, sen, seo, selx* |
| (*S. aureus*/ RKI) | SE | - | | - | | | | - | | | | - | - | + | | | - | | + | | | |
| 21-ST00003 | *se* | - | - | - | - | - | - | - | - | - | - | - | - | - | - | - | - | - | - | - | - | - |
| (*S. aureus*/ BfR) | SE | - | | - | | | | - | | | | - | - | - | | | - | | - | | | |

| Isolates (species/ source) | | *sea* / SEA | | *seb* / SEB | | | | *sec* / SEC | | | | *sed* / SED | *see* / SEE | *seg* / SEG | | | *seh* / SEH | | *sei* / SEI | | | Other *se* |
|---|---|---|---|---|---|---|---|---|---|---|---|---|---|---|---|---|---|---|---|---|---|---|
| | | v1 | v2 | v1 | v2 | v3 | v4 | v1 | v2 | v3 | v4 | | | v1 | v2 | v3 | v1 | v2 | v1 | v2 | v3 | |
| 21-ST00243-2 | *se* | + | - | - | - | - | - | - | - | - | - | + | - | - | - | - | - | - | - | - | - | *selj, ser* |
| (*S. aureus*/ BfR) | SE | + | | - | | | | - | | | | + | - | - | | | - | | - | | | |
| 21-ST00357 | *se* | - | - | - | - | - | - | - | - | - | - | - | - | - | - | - | - | - | - | - | - | - |
| (*S. aureus*/ BfR) | SE | - | | - | | | | - | | | | - | - | - | | | - | | - | | | |
| 21-ST00358 | *se* | - | - | - | - | - | - | - | - | - | - | - | - | - | - | - | - | - | - | - | - | - |
| (*S. aureus*/ BfR) | SE | - | | - | | | | - | | | | - | - | - | | | - | | - | | | |
| 22-00027 | *se* | - | - | - | - | - | - | - | - | - | - | - | - | + | - | - | - | - | + | - | - | *sem, sen, seo, selx* |
| (*S. aureus*/ RKI) | SE | - | | - | | | | - | | | | - | - | + | | | - | | - | | | |
| 18-ST00708 | *se* | - | - | - | - | - | - | - | - | - | - | - | - | - | - | - | - | - | - | - | - | - |
| (*S. epidermidis*/ BfR) | SE | - | | - | | | | - | | | | - | - | - | | | - | | - | | | |
| 19-ST00512 | *se* | - | - | - | - | - | - | - | - | - | - | - | - | - | - | - | - | - | - | - | - | - |
| (*S. haemolyticus*/ BfR) | SE | - | | - | | | | - | | | | - | - | - | | | - | | - | | | |
| 19-ST00518 | *se* | - | - | - | - | - | - | - | - | - | - | - | - | - | - | - | - | - | - | - | - | - |
| (*S. epidermidis*/ BfR) | SE | - | | - | | | | - | | | | - | - | - | | | - | | - | | | |
| 19-ST00686 | *se* | - | - | - | - | - | - | - | - | - | - | - | - | - | - | - | - | - | - | - | - | - |
| (*S. epidermidis*/ BfR) | SE | - | | - | | | | - | | | | - | - | - | | | - | | - | | | |
| 20-ST00131 | *se* | - | - | - | - | - | - | - | - | - | - | - | - | - | - | - | - | - | - | - | - | - |
| (*E. faecalis*/ BfR) | SE | - | | - | | | | - | | | | - | - | - | | | - | | - | | | |
| 20-ST00485 | *se* | - | - | - | - | - | - | - | - | - | - | - | - | - | - | - | - | - | - | - | - | - |
| (*S. epidermidis*/ BfR) | SE | - | | - | | | | - | | | | - | - | - | | | - | | - | | | |
| 21-ST00072 | *se* | - | - | - | - | - | - | - | - | - | - | - | - | - | - | - | - | - | - | - | - | - |
| (*S. epidermidis*/ BfR) | SE | - | | - | | | | - | | | | - | - | - | | | - | | - | | | |

| Isolates | | ***sea* / SEA** | | ***seb* / SEB** | | | | ***sec* / SEC** | | | | ***sed* /** | ***see* /** | ***seg* / SEG** | | | ***seh* / SEH** | | ***sei* / SEI** | | | **Other *se*** |
|---|---|---|---|---|---|---|---|---|---|---|---|---|---|---|---|---|---|---|---|---|---|---|
| **(species/ source)** | | **v1** | **v2** | **v1** | **v2** | **v3** | **v4** | **v1** | **v2** | **v3** | **v4** | **SED** | **SEE** | **v1** | **v2** | **v3** | **v1** | **v2** | **v1** | **v2** | **v3** | |
| 21-ST00078 (*S. epidermidis*/ BfR) | *se* | - | - | - | - | - | - | - | - | - | - | - | - | - | - | - | - | - | - | - | - | - |
| | SE | - | | - | | | | - | | | | - | - | - | | | - | | - | | | |
| 21-ST00300 (*S. epidermidis*/ BfR) | *se* | - | - | - | - | - | - | - | - | - | - | - | - | - | - | - | - | - | - | - | - | - |
| | SE | - | | - | | | | - | | | | - | - | - | | | - | | - | | | |
| **Summarised results for SE detection** | | | | | | | | | | | | | | | | | | | | | | |
| True positive (*n*) | | 24 | | 13 | | | | 31 | | | | 12 | 3 | 71 | | | 14 | | 75 | | | n.a. |
| False positive (*n*) | | 2 | | 0 | | | | 0 | | | | 2 | 0 | 0 | | | 0 | | 3 | | | n.a. |
| True negative (*n*) | | 119 | | 132 | | | | 113 | | | | 129 | 142 | 66 | | | 131 | | 62 | | | n.a. |
| False negative (*n*) | | 0 | | 0 | | | | 1 | | | | 2 | 0 | 8 | | | 0 | | 5 | | | n.a. |
| **Median performance parameters** | | | | | | | | | | | | | | | | | | | | | | |
| Specificity (%) | | 98.3 | | 100 | | | | 100 | | | | 98.5 | 100 | 100 | | | 100 | | 95.4 | | | n.a. |
| Sensitivity (%) | | 100 | | 100 | | | | 96.9 | | | | 85.7 | 100 | 89.9 | | | 100 | | 92.5 | | | n.a. |
| Accuracy (%) | | 98.6 | | 100 | | | | 99.3 | | | | 97.2 | 100 | 94.5 | | | 100 | | 93.8 | | | n.a. |
| **Threshold** | | | | | | | | | | | | | | | | | | | | | | |
| Threshold ($A_{450\text{-}620nm}$) | | 0.4 | | 1.0 | | | | 1.1 | | | | 0.4 | 1.1 | 0.4 | | | 0.9 | | 0.4 | | | n.a. |
| Threshold (pg/mL) ± SD | | 8.5 ± 1 | | 18.6 ± 2 | | | | 18.8 ± 2 | | | | 26.0 ± 2 | 53.9 ± 9 | 12.8 ± 1 | | | 29.6 ± 2 | | 45.2 ± 5 | | | n.a. |

Reference sequences of the *se* gene variants of the VirulenceFinder database considered in this study can be accessed with the following accession IDs (the numbering of the gene variants can differ from the numbering in the database): *sea.v1* (AP009324.1), *sea.v2* (CP010526.1), *seb.v1* (CP007539.1), *seb.v2* (AB716349.1), *seb.v3* (AB716351.1), *seb.v4* (AB716352.1), *sec.v1* (AB084256.1), *sec.v2* (KF386012.1), *sec.v3* (KF729631.1), *sec.v4* (M28364.1), *sed* (M28521.1), *see* (M21319.1), *seg.v1* (CP001844.2), *seg.v2* (CP002388.1), *seg.v3* (AJ938182.1), *seh.v1* (BX571857.1), *seh.v2* (AY345144.1), *sei.v1* (BA000018.3), *sei.v2* (AJ938182.1), and *sei.v3* (CP002388.1). In regard to the subtypes of SEC, SEC1 is encoded by *sec.v2* and *sec.v3*, SEC2 by *sec.v4*, and SEC3 by *sec.v1*. SE = staphylococcal enterotoxin (protein detection), + = *se* / SE present, - = *se* genes / SE not present, grey = *se* present, green = true positive, yellow = false positive, blue = true negative, orange = false negative, * no promotors, ** truncated gene, SD = standard deviation, n.a. = not applicable. Threshold was calculated by receiver operating characteristic curve analysis and the results were extrapolated to the toxin dilution curve, see Figure S11.

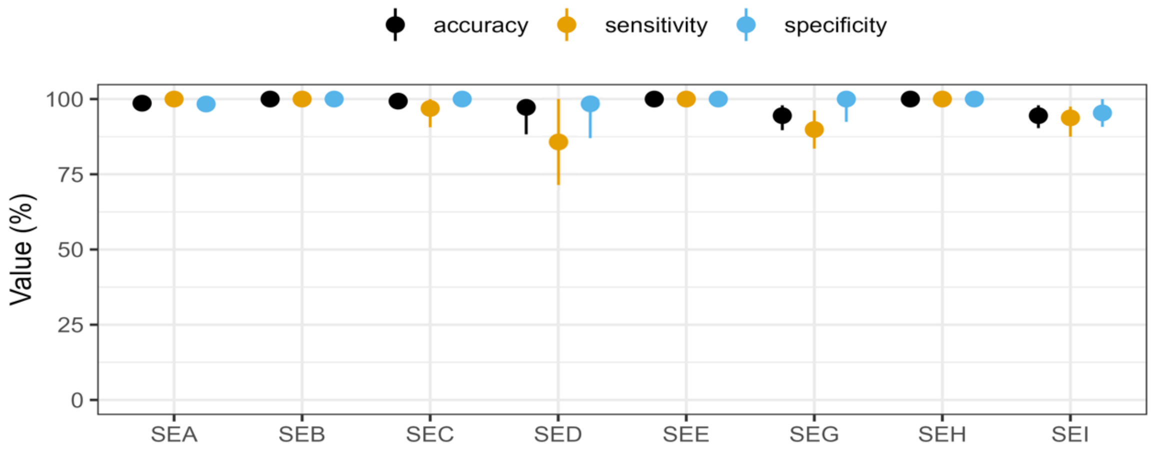


**Figure S12.** Performance parameters of conventional sandwich ELISAs for the detection of native toxins and variants in bacterial culture supernatants. After immobilising capture mAbs (SEA388 + SEA2353 for SEA, S1001 for SEB, SEC371 for SEC, SED1280 for SED, SEE33 for SEE, SEG5 for SEG, SEH1236 for SEH, and SEI467 for SEI) on microtitre plates, a total of 145 culture supernatants were incubated as 1:10 diluted samples and detected by single specific biotinylated antibodies (SEA165 for SEA, S419 for SEB, SEC290 for SEC, SED333 for SED, SEE1524 for SEE, SEG158 for SEG, SEH449 for SEH, and SEI92 for SEI) and streptavidin polyHRP. Results from three independent experiments ($n$ = 3), analysed via ROC analysis (pROC package, version 1.18.0) using R (version 4.1.3), were compared to whole genome sequencing data of the strains and calculated as performance metrics. The data are presented with median values (dots) and confidence intervals (bars) for each SE detected in its specific sandwich ELISA, using three colours to represent performance metrics: accuracy (black), sensitivity (orange), and specificity (blue).

**Enterotoxin type A (SEA)**
Strain: 16-ST00147
Protein sequence coverage: 94%

```
  1 MKKTAFTLLL FIALTLTTSP LVNGSEKSEE INEKDLRKKS ELQGTALGNL
 51 KQIYYYNEKA KTENKESHDQ FLQHTILFKG FFTDHSWYND LLVDFDSKDI
101 VDKYKGKKVD LYGAYYGYQC AGGTPNKTAC MYGGVTLHDN NRLTEEKKVP
151 INLWLDGKQN TVPLETVKTN KKNVTVQELD LQARRYLQEK YNLYNSDVFD
201 GKVQRGLIVF HTSTEPSVNY DLFGAQGQYS NTLLRIYRDN KTINSENMHI
251 DIYLYTS
```

**Enterotoxin type B (SEB)**
Strain: 15-ST00430
Protein sequence coverage: 97%

```
  1 MYNRLFVSRV ILIFALILVI YTPNVLAESQ PDPKPDELHK ASKFTGLMEN
 51 MKVLYDDNHV SAINVKSIDQ FLYFDLIYSI KDTKLGNYDN VRVEFKNKDL
101 ADKYKDKYVD VFGANYYYQC YFSKKTNDIN SHQTDKRKTC MYGGVTEHNG
151 NHLDKYRSIT VRVFEDGKNL LSFDVQTNKK KVTAQELDYL TRHYLVKNKK
201 LYEFNNSPYE TGYIKFIESE NSFWYDMMPA PGDKFDQSKY LMMYNDNKLV
251 DSKDVKIEVY LTTKKK
```

Strain: 16-ST00052
Protein sequence coverage: 98%

```
  1 MYKRLFISHV ILIFVLILVI STPNVLAESQ PDPKPDELHK ASKFTGLMEN
 51 MKVLYDDNHV SAINVKSIDQ FLYFDLIYSI KDTKLGNYDN VRVEFKNKDL
101 ADKYKDKYVD VFGANYYYQC YFSKKTNDIN SHQTDKRKTC MYGGVTEHNG
151 NQLDKYRSIT VRVFEDGKNL LSFDVQTNKK KVTAQELDYL TRHYLVKNKK
201 LYEFNNSPYE TGYIKFIESE NSFWYDMMPA PGDKFDQSKY LMMYNDNKLV
251 DSKDVKIEVY LTTKKK
```

**Enterotoxin type C-1 (SEC-1)**
Strain: 15-ST00440
Protein sequence coverage: 77%

```
  1 MNKSRFISCV ILIFSLILVL FTPNVLAESQ PDPTPDELHK ASKFTGLMEN
 51 MKVLYDDRYV SATKVKSVDK FLAHDLIYNI SDKKLKNYDK VKTELLNEDL
101 AKKYKDEVVD VYGSNYYVNC CFSSKDNVGK VTGGKTCMYG GITKHEGNHF
151 DNGNLQNVLI RVYENKRNTI SFEVQTDKKS VTAQELDIKA RSFLINKKNL
201 YEFNSSPYET GYIKFIENNG NTFWYDMMPA PGDKFDQSKY LMMYNDNKTV
251 DSKSVKIEVH LTTKNG
```

**Enterotoxin type C-3 (SEC-3)**
Strain: 17-ST00292
Protein sequence coverage: 92%

```
  1 MYKRLFISRV ILIFALILVI STPNVLAESQ PDPMPDDLHK SSEFTGTMGN
 51 MKYLYDDHYV SATKVKSVDK FLAHDLIYNI SDKKLKNYDK VKTELLNEDL
101 AKKYKDEVVD VYGSNYYVNC YFSSKDNVGK VTGGKTCMYG GITKHEGNHF
151 DNGNLQNVLV RVYENKRNTI SFEVQTDKKS VTAQELDIKA RNFLINKKNL
201 YEFNSSPYET GYIKFIENNG NTFWYDMMPA PGDKFDQSKY LMMYNDNKTV
```

251 DSKSVKIEVH LTTKNG

**Enterotoxin type D (SED)**
Strain: 17-ST00026
Protein sequence coverage: 96%

1 MKKFNILIAL LFFTSLVISP LNVKANENID SVKEKELHKK SELSSTALNN
51 MKHSYADKNP IIGENKSTGD QFLENTLLYK KFFTDLINFE DLLINFNSKE
101 MAQHFKSKNV DVYAIRYSIN CYGGEIDKTA CTYGGVTPHE GNKLKERKKI
151 PINLWINGVQ KEVSLDKVQT DKKNVTVQEL DAQARRYLQK DLKLYNNDTL
201 GGKIQRGKIE FDSSDESKVS YDLFDVKGDF PEKQLRIYSD NKTLSTEHLH
251 IDIYLYEK

**Enterotoxin type E (SEE)**
Strain: 08S00575
Protein sequence coverage: 87%

1 MKKTAFILLL FIALTLTTSP LVNGSEKSEE INEKDLRKKS ELQRNALSNL
51 RQIYYYNEKA ITENKESDDQ FLENTLLFKG FFTGHPWYND LLVDLGSKDA
101 TNKYKGKKVD LYGAYYGYQC AGGTPNKTAC MYGGVTLHDN NRLTEEKKVP
151 INLWIDGKQT TVPIDKVKTS KKEVTVQELD LQARHYLHGK FGLYNSDSFG
201 GKVQRGLIVF HSSEGSTVSY DLFDAQGQYP DTLLRIYRDN KTINSENLHI
251 DLYLYTT

**Enterotoxin type G (SEG)**
Strain: 16-ST00052
Protein sequence coverage: 73%

1 MKKLSTVIII LILEIVFHNM NYVNAQPDPK LDELNKVSDY KNNKGTMGNV
51 MNLYTSPPVE GRGVINSRQF LSHDLIFPIE YKSYNEVKTE LENTELANNY
101 KDKKVDIFGV PYFYTCIIPK SEPDINQNFG GCCMYGGLTF NSSENERDKL
151 ITVQVTIDNR QSLGFTITTN KNMVTIQELD YKARHWLTKE KKLYEFDGSA
201 FESGYIKFTE KNNTSFWFDL FPKKELVPFV PYKFLNIYGD NKVVDSKSIK
251 MEVFLNTH

Strain: 17-ST00292
Protein sequence coverage: 87%

1 MKKLSTVIII LILEIVFHNM NYVNAQPDPK LDELNKVSDY KNNKGTMGNV
51 MNLYTSPPVE GRGVINSRQF LSHDLIFPIE YKSYNEVKTE LENTELANNY
101 KDKKVDIFGV PYFYTCIIPK SEPDINQNFG DCCMYGGLTF NSSENERDKL
151 ITVQVTIDNR QSLGFTITTN KNMVTIQELD YKARHWLTKE KKLYEFDGSA
201 FESGYIKFTE KNNTSFWFDL FPKKELVPFV PYKFLNIYGD NKVVDSKSIK
251 MEVFLNTH

**Enterotoxin type H (SEH)**
Strain: 20-ST00482
Protein sequence coverage: 95%

1 MINKIKILFS FLALLLSFTS YAKAEDLHDK SELTDLALAN AYGQYNHPFI
51 KENIKSDEIS GEKDLIFRNQ GDSGNDLRVK FATADLAQKF KNKNVDIYGA
101 SFYYKCEKIS ENISECLYGG TTLNSEKLAQ ERVIGANVWV DGIQKETELI

151 **RTNKKNVTLQ ELDIKIRKIL SDKYKIYYKD SEISKGLIEF DMK**TPR**DYSF**

201 **DIYDLKGEND YEIDKIYEDN KTLKSDDISH IDVNLYTKK**K V

**Enterotoxin type I (SEI)**

Strain: 16-ST00052

Protein sequence coverage: 89%

1 MKKFKYSFIL VFILLFNIKD LTYA**QGDIGV GNLRNFYTKH DYIDLK**GVTD

51 K**NLPIANQLE FSTGTNDLIS ESNNWDEISK** FKGK**KLDIFG IDYNGPCK**SK

101 **YMYGGATLSG QYLNSARKIP INLWVNGK**HK **TISTDKIATN KKLVTAQEID**

151 **VK**LR**RYLQEE YNIYGHNNTG K**GKEYGYKSK **FYSGFNNGKV LFHLNNEKSF**

201 **SYDLFYTGDG LPVSFLKIYE DNKIIESEKF HLDVEISYLD SN**

Strain: 17-ST00292

Protein sequence coverage: 84%

1 MKKFKYSFIL VFILLFNIKD LTYAQGDIGV GNLRNFYTK**H DYIDLKGVTD**

51 **KNLPIANQLE FSTGTNDLIS ESNNWDEISK** FKGK**KLDIFG IDYNGPCK**SK

101 **YMYGGATLSG QYLNSARKIP INLWVNGK**HK **TISTDKIATN KKLVTAQEID**

151 **VK**LR**RYLQEE YNIYGHNNTG K**GKEYGYKSK **FYSGFNNGKV LFHLNNEKSF**

201 **SYDLFYTGDG LPVSFLKIYE DNKIIESEKF HLDVEISYVD SN**

**Figure S13.** Amino acid sequence coverage of different SEs from bacterial culture supernatants after immunoaffinity enrichment, tryptic digest, and tandem mass spectrometry analysis (LC-MS/MS). Amino acid sequence coverage of different SEs from representative bacteria liquid cultures after immunoaffinity enrichment by a mix of highly specific anti-SE antibodies (mAb SEA388, S419, SEC371, SED1280, SEE1524, SEG5, SEH449, and SEI242 directed against SEA, SEB, SEC, SED, SEE, SEG, SEH, and SEI, respectively), reduction, alkylation, tryptic digest, and LC-MS/MS (DDA-PASEF). Reference amino acid sequences for MS database search were obtained by whole genome sequencing of the corresponding *S. aureus* strains. Two strains (16-ST00052 and 17-ST00292) encoded for more than one enterotoxin specific gene. For this analysis, no bacterial liquid culture supernatant produced by a strain encoding sec.v4 (SEC2) was analysed. Identified sequences by MS are shown in red and bold; signal peptides that are not present in mature proteins and therefore cannot be identified by MS are marked in yellow. Protein sequence coverage is calculated without signal peptide.

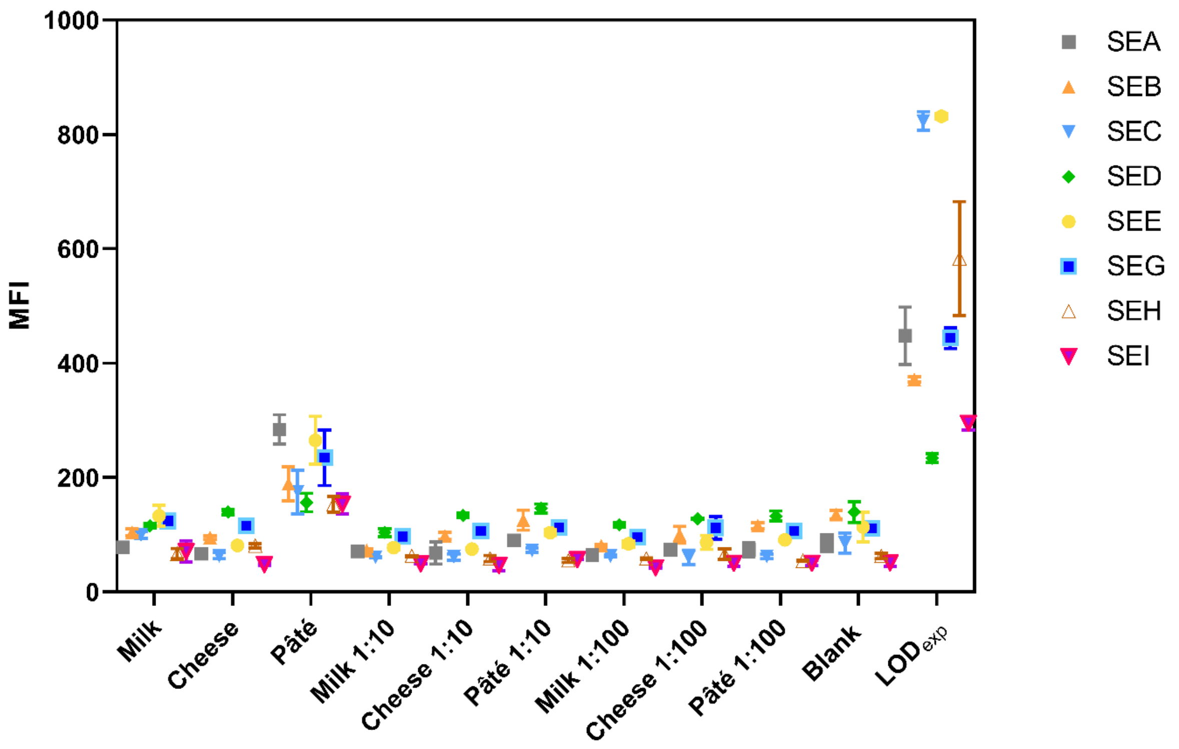


**Figure S14.** Estimation of matrix effects in the multiplex SIA. The mAb coated magnetic beads (SEA388 + SEA2353, S1851, SEC371, SED1280, SEE1524, SEG5, SEH449, and SEI242 targeting SEA, SEB, SEC, SED, SEE, SEG, SEH, and SEI, respectively) were incubated with the food extracts of raw milk, raw milk cheese, and pork pâté in different dilutions (undiluted, 1:10, or 1:100 dilution), and with a mixture containing SEA to SEI spiked to buffer at the concentration of the $LoD_{exp}$. After detection with biotinylated mAb mixture, consisting of mAbs SEA165, S419 for SEB, SEC290, SED9, SEE33, SEG158, SEH1236, and SEI92, addition of streptavidin-phycoerythrin followed. In this experiment measured in duplicates (*n* = 1), the different colours indicate the target of the multiplex SIA. $LOD_{exp}$ represents the experimentally confirmed limit of detection for each target antigen in buffer. Blank consists of 0.1% BSA/PBS buffer, which was used for the dilution of the food extracts and for the measurement of the $LOD_{exp}$.

**Table S4.** Overview of recoveries ± SD in % of toxins spiked to food extracts for multiplex SIA.

| | Spiking concentration | | | | | | |
|---|---|---|---|---|---|---|---|
| | 1 × $EC_{50}$ | | | 10 × $EC_{50}$ | | | |
| | Raw milk | Raw milk cheese | Smoked pork pâté | Raw milk | Raw milk cheese | Smoked pork pâté | Average |
| **SEA** | 35.2 ± 1.4 | 57.3 ± 4.8 | 52.1 ± 4.0 | 79.6 ± 2.3 | 88.7 ± 11.5 | 93.8 ± 13.3 | 67.8 ± 6.2 |
| **SEB** | 43.1 ± 3.7 | 87.1 ± 6.6 | 75.5 ± 10.1 | 72.3 ± 3.8 | 106.9 ± 7.0 | 102.4 ± 4.7 | 81.2 ± 6.0 |
| **SEC** | 79.5 ± 2.9 | 122.7 ± 7.7 | 59.1 ± 4.3 | 96.3 ± 3.0 | 127.3 ± 8.7 | 83.8 ± 5.2 | 94.8 ± 5.3 |
| **SED** | 43.4 ± 1.7 | 58.0 ± 1.5 | 52.0 ± 5.3 | 91.8 ± 1.8 | 105.2 ± 7.9 | 106.4 ± 7.7 | 76.1 ± 4.3 |
| **SEE** | 36.8 ± 1.7 | 53.6 ± 4.1 | 44.9 ± 4.3 | 85.0 ± 1.9 | 103.5 ± 9.8 | 108.8 ± 12.0 | 72.1 ± 5.6 |
| **SEG** | 9.7 ± 0.7 | 53.8 ± 6.1 | 68.3 ± 10.2 | 36.7 ± 2.7 | 85.8 ± 8.0 | 93.8 ± 17.7 | 58.0 ± 7.6 |
| **SEH** | 49.3 ± 4.6 | 76.4 ± 5.3 | 11.0 ± 1.3 | 94.3 ± 1.4 | 87.3 ± 7.9 | 51.4 ± 4.1 | 61.6 ± 4.1 |
| **SEI** | 19.9 ± 0.6 | 32.2 ± 5.1 | 4.5 ± 0.5 | 47.2 ± 1.6 | 65.3 ± 1.6 | 31.7 ± 1.4 | 33.5 ± 1.8 |
| **Average** | 39.6 ± 2.2 | 67.6 ± 5.2 | 45.9 ± 5.0 | 75.4 ± 2.3 | 96.3 ± 7.8 | 84.0 ± 8.3 | |
